# Screening of autoantibodies as biomarkers in the serum of renal cancer patients based on human proteome microarray: Screening of autoantibodies in renal cancer

**DOI:** 10.3724/abbs.2022189

**Published:** 2022-12-23

**Authors:** Yangyang Sun, Chengxi Liu, Huidong Zhong, Chenguang Wang, Haibo Xu, Wei Chen

**Affiliations:** 1 Shenzhen Key Laboratory of Synthetic Genomics Guangdong Provincial Key Laboratory of Synthetic Genomics CAS Key Laboratory of Quantitative Engineering Biology Shenzhen Institute of Synthetic Biology Shenzhen Institutes of Advanced Technology Chinese Academy of Sciences Shenzhen 518055 China; 2 Department of Urology Shenzhen Second People’s Hospital the First Affiliated Hospital of Shenzhen University International Cancer Center Shenzhen University School of Medicine Shenzhen 518039 China; 3 State Key Laboratory of Chemical Biology and Drug Discovery Food Safety and Technology Research Centre and Department of Applied Biology and Chemical Technology The Hong Kong Polytechnic University Hong Kong 999077 China; 4 Department of Medicinal Chemistry Shantou University Medical College Shantou 515041 China

**Keywords:** renal cancer, autoantibody, biomarker, human proteome microarray

## Abstract

The autoantibody in patients’ serum can act as a biomarker for diagnosing cancer, and the differences in autoantibodies are significantly correlated with the changes in their target proteins. In this study, 16 renal cancer (RC) patients were assigned to the disease group, and 16 healthy people were assigned to the healthy control (HC) group. The human proteome microarray consisting of>19,500 proteins was used to examine the differences in IgG and IgM autoantibodies in sera between RC and HC. The comparative analysis of the microarray results shows that 101 types of IgG and 25 types of IgM autoantibodies are significantly higher in RC than in HC. Highly responsive autoantibodies can be candidate biomarkers (e.g., anti-KCNAB2 IgG and anti-RCN1 IgM). Extensive enzyme-linked immunosorbent assay (ELISA) was performed to screen sera in 72 RC patients and 66 healthy volunteers to verify the effectiveness of the new autoantibodies. The AUCs of anti-KCNAB2 IgG and anti-GAPDH IgG were 0.833 and 0.753, respectively. KCNAB2 achieves high protein expression, and its high mRNA level is confirmed to be an unfavorable prognostic marker in clear cell renal cell carcinoma (ccRCC) tissues. This study suggests that the high-throughput human proteome microarray can effectively screen autoantibodies in serum as candidate biomarkers, and their corresponding target proteins can lay a basis for the in-depth investigation into renal cancer.

## Introduction

The estimated new cases of renal cancer have been ranked in the top 10 of all cancer types, whether male or female [1]. The 5-year survival rate of patients suffering from early-stage renal cancer can account for 93%. Eighty percent of renal cancer patients are asymptomatic at the early stage, and 34.8% of patients have cancer metastases when diagnosed. Surgery has been recognized as the most effective treatment, whereas the recurrence rate of renal cancer patients 3 years after surgery reached up to 31.1% [2]. In accordance with Chinese guidelines for the diagnosis and treatment of renal cell carcinoma (2021 Edition), early-stage renal cancer often lacks clinical manifestations. When the classic triad of renal cancer (hematuria, low back pain, and abdominal mass) appears, most of the patients become middle-advanced patients. Most kidney cancer patients are found inadvertently through imaging tests when the tumor turns out to be large. The diagnosis of renal cancer lacks an early and simple serological diagnostic scheme. On that basis, early diagnosis and prognostic monitoring of renal cancer have critical significance. Screening RC-related tumor biomarkers from serum is considered a prospective research field.

Changes in protein expression, structure and function are vital factors for the progression of renal cancer. In existing studies, mass spectrometry methods have been employed to discover biomarkers and signaling pathways significantly correlated with renal cancer [ 3‒ 6] . The discovery of the above biomarkers has deepened the understanding of the diagnosis, prognosis monitoring and drug treatment of renal cancer. Through overexpression, underexpression, degradation or mutation of proteins in tissues, tumor-specific or tumor-related antigens can be produced, which are released from tumor tissues into the blood, so the patients’ immune system will produce autoantibodies. Autoantibodies can act as tumor biomarkers in serum for the diagnosis and postoperative monitoring of tumors [ 7‒ 10] .

There have been highly expressed proteins in renal cancer tissues (e.g., livin and arrestin-1), and in-depth studies reported that the autoantibodies of the two target proteins in cancer patients are significantly higher than those in healthy controls [ 11, 12] . To screen autoantibodies in the sera of renal cancer patients at a large scale, the serological analysis of recombinant cDNA expression libraries (SEREX) method has been proposed. Sixty-five renal cancer-related antigens were reported, and the autoantibodies reacting with them were explored [13]. Moreover, there have been few reports on systemic autoantibody screening for renal cancer. With the development of microarray technology, high-throughput human proteome microarray has contained over 19,500 human recombinant proteins, which leads to its advantages of high sensitivity, high specificity, low sample volume, simple operation, and short time consumption. It has been extensively used to screen autoantibodies against autoimmune diseases and various tumors [ 14‒ 17] .

This study aimed to identify potential autoantibodies in the sera of patients suffering from renal cancer and their corresponding target proteins using a high-throughput human proteome microarray, as well as to present more novel biomarker information for the further diagnosis and even targeted therapy of renal cancer.

## Materials and Methods

### Collection of serum samples

The serum samples of RC and HC originated from the Department of Urology, Shenzhen Second People′s Hospital (Shenzhen, China). This study was approved by the Ethics Committee of the Hospital (#2021011007), and all patients and volunteers signed informed consent forms. Serum samples were obtained prior to any surgical intervention, and serum samples from patients with renal cancers were collected as the experimental samples after the surgical tissue was pathologically confirmed as renal cancers. The sera of 88 renal cancer patients and 82 healthy people were prepared in accordance with standard procedures. In brief, 5 mL of whole blood was collected with blood collection tubes without any anticoagulant, which were then placed at ambient temperature for 30 min and subsequently centrifuged with a refrigerated centrifuge at 4°C for 10 min at 2000 *g*. The upper serum was separated into 0.5 mL aliquots in each tube and then stored at ‒80°C.

### Human proteome microarray

The human proteome microarray was obtained from CDI Laboratories, Inc (HuProt array version 3.1, Baltimore, USA). The microarray contained>19,500 human proteins encoded by 16,152 human genes, accounting for nearly 81% of human proteins. The fusion proteins with N-terminal glutathione S-transferase (GST) tags were expressed using *Saccharomyces cerevisiae* and then purified with glutathione agarose beads. Subsequently, the proteins were printed on the modified glass substrate using the spotting robot. In addition to the recombinant protein, histones H3 and H4 were used as the positive controls, and BSA and biotinylated BSA were used as the negative controls. Two spots were repeated for the respective protein. Furthermore, the human proteome microarray was stored at ‒80°C.

### Protein microarray for serum analysis

The experiments were performed as described in a previous study [18]. The microarray was removed from ‒80°C and restored to ambient temperature, placed in PBST (PBS with 0.5% Tween-20) supplemented with 3% BSA, and then incubated at ambient temperature for 3 h to block the microarray. Serum samples (25 μL) were diluted into 5 mL of PBST supplemented with 1% BSA. Subsequently, the blocked microarray was placed in serum diluent and then incubated with a lateral shaker at 40 rpm and 4°C for 12 h. The microarray was washed with PBST 3 times for 10 min each time. Goat anti-human IgG (Cy3 labelled) and donkey anti-human IgM (Cy5 labelled) were diluted at a dilution ratio of 1:1000 in 5 mL PBST. Next, the microarray was added to the fluorescence-labelled antibody dilution solution and then incubated at ambient temperature in the dark for 1 h. Afterward, the microarray was washed with PBST 3 times for 10 min each time and then cleaned with ddH _2_O for 10 sec. After the microarray was dried with the spin dryer, the signal value of the microarray was read using the microarray scanner. Furthermore, the foreground and background values of the points were analysed using Genepix Pro 6.0. The SNR value was equal to the foreground value (F)/background value (B) of the respective point, and the SNRs of two repeated points were averaged as the final SNR value.

### Data analysis of microarray

The normalization between microarrays was conducted in accordance with the median value of all protein spots to eliminate the systematic errors arising from the experimental samples and the experimental operations. The 32 samples were statistically analyzed to screen out autoantibodies capable of distinguishing RC from HC, which consisted of significantly high response (Up) autoantibodies and significantly low response (Down) autoantibodies. If IgG-SNR≤4 and IgM-SNR≤5 of all samples in RC and HC on a certain protein, it was determined as a negative spot and excluded directly. The ratio of the mean value of RC to HC was the fold change (FC). The positive ratio was obtained as the number of RC or HC positive reactivities to its sum [19]. The optimal cut-off value for the respective candidate biomarker was evaluated based on two criteria, including 1) at least 90% specificity and 2) the highest discriminant ability. The screening criteria of Up autoantibodies and their target proteins included RC positive ratio>30%, HC positive ratio<10%, FC≥1.5 and the *P* value of *t*-test<0.05. Furthermore, the screening criteria of Down autoantibodies included HC positive ratio>30%, RC positive ratio<10%, FC<2/3 and the *P* value of *t*-test<0.05. The screening criteria of unchanged autoantibodies and their target proteins included positive ratios consistent with Up or Down, whereas FC or the *P* value were inconsistent with Up or Down. Key factors of Up autoantibodies were found using the randomForest package.

### Protein expression and purification

*Escherichia* c *oli* expression clones encoding recombinant human KCNAB2, GAPDH, OCM and LIVIN were prepared by General Biosystems, Ltd. (Chuzhou, Anhui). The respective recombinant human protein was overexpressed in *E*. *coli*, in accordance with a previous description [20]. In brief, the cells were harvested through centrifugation and washed with phosphate buffered saline (PBS). Lysis buffer was added, and the supernatant was collected through centrifugation. The recombinant protein was captured by using Ni-NTA beads (QIAGEN, Hilden, Germany) and then incubated at 4°C for 2 h based on vigorous shaking. After several rounds of washing, the proteins were eluted and desalted with a Zeba ^TM^ Spin Desalting Column (Thermo Scientific, Waltham, USA). Furthermore, the protein concentrations were obtained through absorbance determination with a Nanodrop spectrophotometer (Thermo Scientific, Waltham, USA) at 280 nm [21].

### Autoantibody ELISA detection

An indirect enzyme-linked immunosorbent assay (ELISA) was designed to measure the level of autoantibodies in human serum. In brief, microtiter plates were coated with recombinant human proteins, including KCNAB2, GAPDH, OCM and LIVIN (overnight incubation at 4°C, 50 μL/well, 10 μg/mL). The plates were washed with 1 × PBS three times and then blocked with 5% (w/v) BSA (3‒4 h, 37°C). Subsequently, the plates were incubated with human sera diluted in the assay diluent (2 μL/mL, 100 μL/well, at ambient temperature). After the subsequent washing 6 times with PBST, the plates were incubated with peroxidase-labelled goat anti-human IgG (1 h at 37°C, 100 μL/well, 1 in 10,000 dilution), and the antibody was provided by Abcam (ab6858; Cambridge, UK). After the subsequent washing 6 times with PBST, tetramethylbenzidine (T820901; Macklin Inc, Rochelle, USA) was employed as the substrate for the peroxidase reaction (100 μL/well, at ambient temperature), and the enzymatic reaction was stopped after 10‒30 min with addition of 1 N HCl (ambient temperature, 50 μL/well). The optical density (OD) was read at 450 nm with an ELISA plate reader (PerkinElmer, Boston, USA). Human IgG (50 μg/mL) was assigned into a positive control, and bovine serum albumin (50 μg/mL) was assigned into a negative control in respective microtiter plates to obtain relative OD values. The SNR value=(OD value of the mean of the positive control-OD value of the mean of the negative control)/OD value of the mean of the negative control.

### Analyses of TCGA data

The gene expression profiles (FPKM normalized) of the ccRCC patients from the TCGA cohort were downloaded using the TCGAbiolinks package and then transformed into TPM levels. The survival analysis of KCNAB2 was conducted using the survival package based on the beast cut-off value. The lowest cut-off value was found using the ‘surv_cutpoint’ function in the survminer package.

### Western blot analysis

Renal cancer tissues and normal tissues were lysed in RIPA buffer (Cat# 9806s; Cell Signaling Technology, Beverly, USA) with the addition of 1 mM phenylmethanesulfonyl fluoride (PMSF) immediately before use. Twenty-five micrograms of total protein was separated by SDS‒PAGE and then transferred to PVDF membranes. A KCNAB2 polyclonal antibody (17890-1-AP; Proteintech Group, Inc., Rosemont, USA) was employed at a dilution of 1:1000. Finally, the western blots were quantified with ImageJ software.

## Results

### The working principle of the human proteome microarray to detect serum autoantibodies

**Figure 1:**
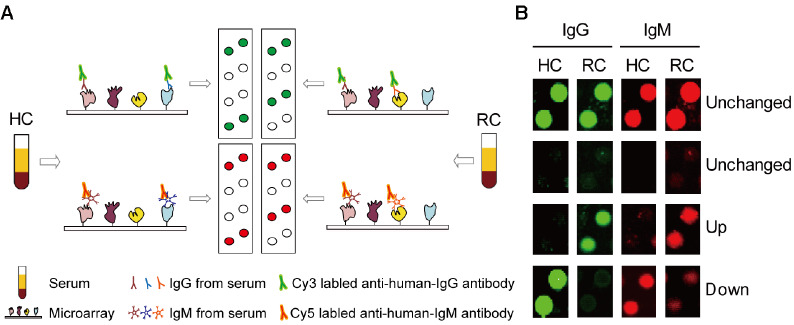
Figure 1 Screening of serum autoantibodies correlated with renal cancer using a human proteome microarray (A) Schematic of the human proteome microarray applied for screening autoantibodies in serum. (B) Examples of the signal of the autoantibodies reacting with target proteins on the microarray. For most target proteins, the serum autoantibodies of HC and RC showed consistent responses (Unchanged), whereas for some target proteins, RC serum autoantibodies showed a higher degree of response (Up) than HC, and some showed the opposite (Down).

Thirty-two human proteome microarrays were used to profile serum samples from 16 healthy volunteers and 16 renal cancer patients ( Table 1), and the workflow is illustrated in Figure 1A. Anti-human antibodies showing different fluorescence types were adopted to mark IgG or IgM in serum, which could bind to the recombinant proteins of the microarray. The goat anti-human IgG (labelled by Cy3) antibody showed green fluorescence, while the donkey anti-human IgM (labelled by Cy5) antibody showed red fluorescence. As indicated by the comparison between RC and HC, for IgG or IgM, there were 3 possible results based on the microarray: 1) Unchanged, autoantibodies of RC and HC did not change significantly for most target proteins; 2) Up, autoantibodies of RC were more responsive than HC for some target proteins; 3) Down, autoantibodies of RC serum were less responsive than those of HC for the other proteins of the microarray. The above three results suggested that the corresponding target proteins were found in the experimental results ( Figure 1B).

**Table:** **Table 1** Gender and age information of RC and HC patients for microarray

Group	Cases	Gender ratio F/M	Age range	Average age± standard deviation
RC	16	4:12	38‒70	55.13±9.37
HC	16	7:9	34‒70	52.94±10.72

### Statistical analysis of microarray results

**Figure 2:**
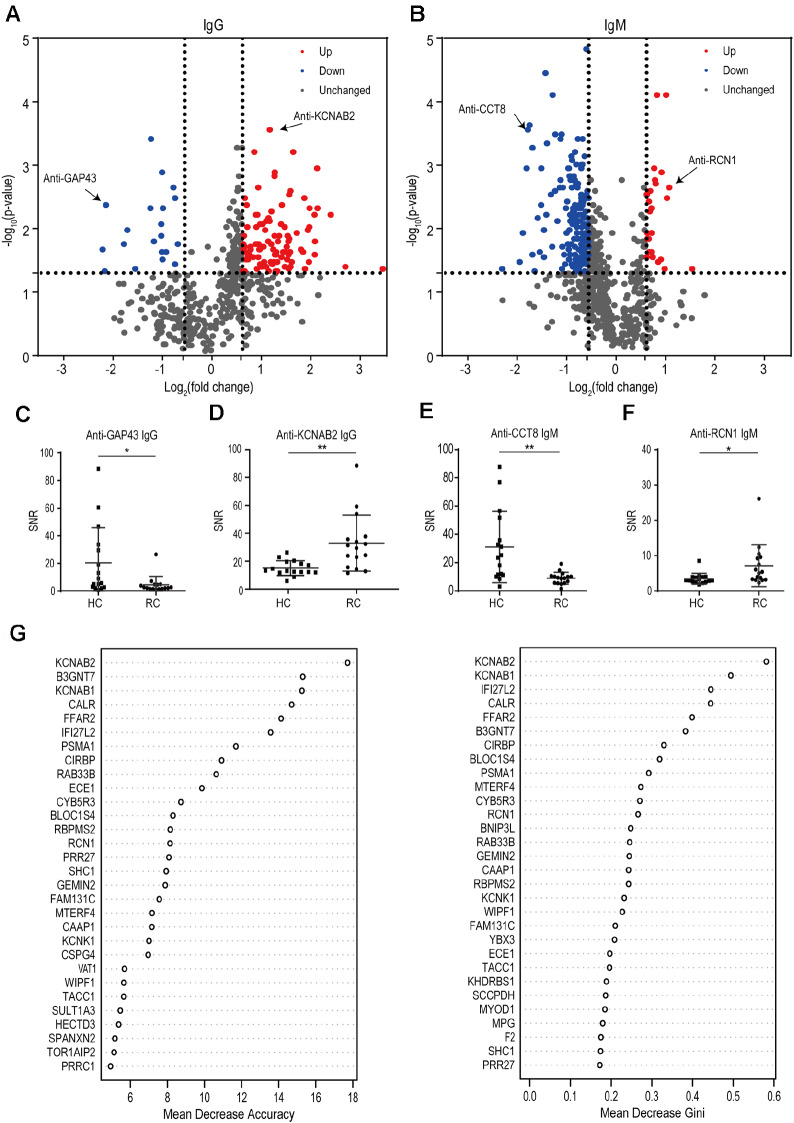
Figure 2 Candidate autoantibodies correlated with renal cancer (A) IgG volcano plot to comparing RC patients with HCs. (B) IgM volcano plot comparing RC patients with HCs. Unchanged autoantibodies are represented by gray color. The red color represents Up autoantibodies, and green represents Down autoantibodies. (C‒F) There were significant differences in the examples of autoantibodies between RC and HC. * P<0.05, ** P<0.01. (G) Random forest analysis of the Up autoantibodies distinguishing RC and HC. Up autoantibodies are ranked by order of importance.

A statistical analysis was conducted for the signal of the respective protein on the microarray, and the criteria were set to screen autoantibodies and their target proteins in the ″Methods″ section. The volcano plot illustrates all Up, Down and Unchanged autoantibodies of IgG and IgM in RC ( Figure 2A,B). Specifically, 316 autoantibodies changed significantly. Supplementary Table S1 lists the cut-off, positive rate, fold change and *P* value of all target proteins. According to the comparison between RC and HC, Up autoantibodies consisted of 101 types of IgG and 25 types of IgM, while Down autoantibodies contained 21 types of IgG and 169 types of IgM. Among IgG autoantibodies, the number of Up autoantibodies was significantly higher than that of Down autoantibodies, whereas in IgM autoantibodies, the statistical results were the opposite. Some Up autoantibodies could be employed as candidate biomarkers. Specifically, anti-KCNAB2 IgG and anti-RCN1 IgM had high positive rates of 56.25% (9/16) and 31.25% (5/16), respectively, thus indicating that KCNAB2 and RCN1 might be target proteins of RC-related autoantibodies ( Figure 2D,F). Figure 2G shows the 30 most important Up autoantibodies as assessed by random forest regression. Anti-KCNAB2 IgG was clearly the most important discriminative variable based on both Mean Decrease Accuracy and Mean Decrease Gini results. Moreover, Down autoantibodies were rarely used as candidate biomarkers, whereas their targeted proteins were significantly correlated with renal cancer. For example, anti-GAP43-IgG and anti-CCT8-IgM in RC were significantly lower than those in HC ( Figure 2C,E). Among the 316 differential autoantibodies screened out in this study, anti-KCNAB2, anti-GAR1, anti-RCN1 and anti-KHDRBS1 autoantibodies were Up in IgG and IgM, while anti-GAP43, anti-IFIT3, anti-SNAP91 and anti-TEX29 autoantibodies were Down in IgG and IgM.

### ELISA validation of the biomarkers

To further validate the screened candidate biomarkers, a more clinically friendly ELISA platform was developed for four proteins. KCNAB2, GAPDH and OCM proteins were selected based on the human proteome microarray results. In addition, LIVIN protein was reported, and its autoantibody level was found to be higher in RC than in HC. Two cohorts, including 66 healthy control sera and 72 renal cancer patient sera, were assembled. The renal cancer patient sera consisted of 46 T1 stage samples and 26 T2 & T3 stage samples ( Table 2).

**Table:** **Table 2** Characteristics of the samples for ELISA

Variable	RC ( *n*=72)	HC ( *n*=66)
NO.	Mean	%	NO.	Mean	%
Age (years)						
Mean		57.13			51.61	
Standard deviation		11.32			13.81	
Sex						
Male	53		73.61	44		66.67
Female	19		26.39	22		33.33
Type						
Clear cell renal cell carcinoma	65		90.28			
Papillary renal cell carcinoma	4		5.55			
Chromophobe renal cell carcinoma	3		4.17			
T stage						
T1	46		63.89			
T2	4		5.55			
T3	22		30.56			

**Figure 3:**
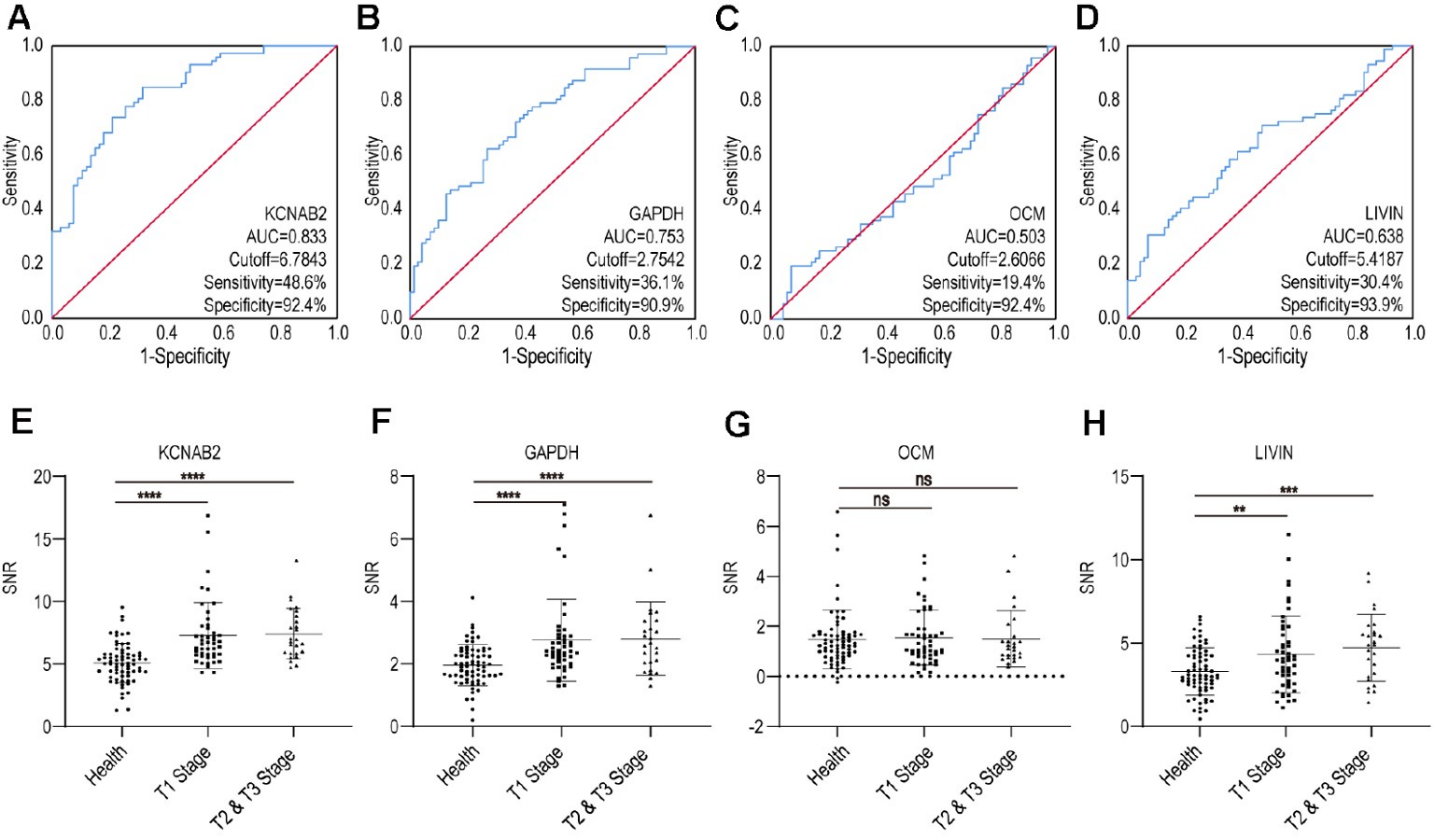
Figure 3 Autoantibody signals found in healthy volunteers and patients with renal cancer using indirect ELISA (A‒D) Receiver operating characteristic curve analysis of the four serum biomarkers for renal cancer diagnosis. KCNAB2, GAPDH, OCM and LIVIN were employed as the capture antigens. (E,F) Scatter plot analysis of ELISA data obtained from samples of healthy controls or patients with different T stages. P values were obtained between the different stage RC and control groups. ** P<0.01, *** P<0.001, **** P<0.0001.

KCNAB2 exhibited 48.6% sensitivity and 92.4% specificity for RC detection (RC *vs* HC, AUC=0.833) ( Figure 3A), GAPDH exhibited 36.1% sensitivity and 90.9% specificity for RC detection (RC *vs* HC, AUC=0.753) ( Figure 3B), OCM exhibited 19.4% sensitivity and 92.4% specificity for RC detection (RC *vs* HC, AUC=0.503) ( Figure 3C), and LIVIN was found to have 30.6% sensitivity and 93.9% specificity for RC detection (RC *vs* HC, AUC=0.638) ( Figure 3D). KCNAB2 and GAPDH of the three candidates from the microarray could act as biomarkers for RC detection, since they had higher AUCs than LIVIN. Although OCM showed no significant difference, KCNAB2, GAPDH and LIVIN showed significant difference between healthy controls and patients suffering from T1 tumors ( Figure 3E‒H). In brief, KCNAB2 and GAPDH could act as candidate biomarkers to identify patients suffering from renal cancer and even early renal cancer. KCNAB2 and GAPDH were combined as a panel, and the AUC of the panel increased slightly to 0.840, while the panel exhibited 48.6% sensitivity and 92.4% specificity for RC detection.

### Differentially expressed tissue KCNAB2

**Figure 4:**
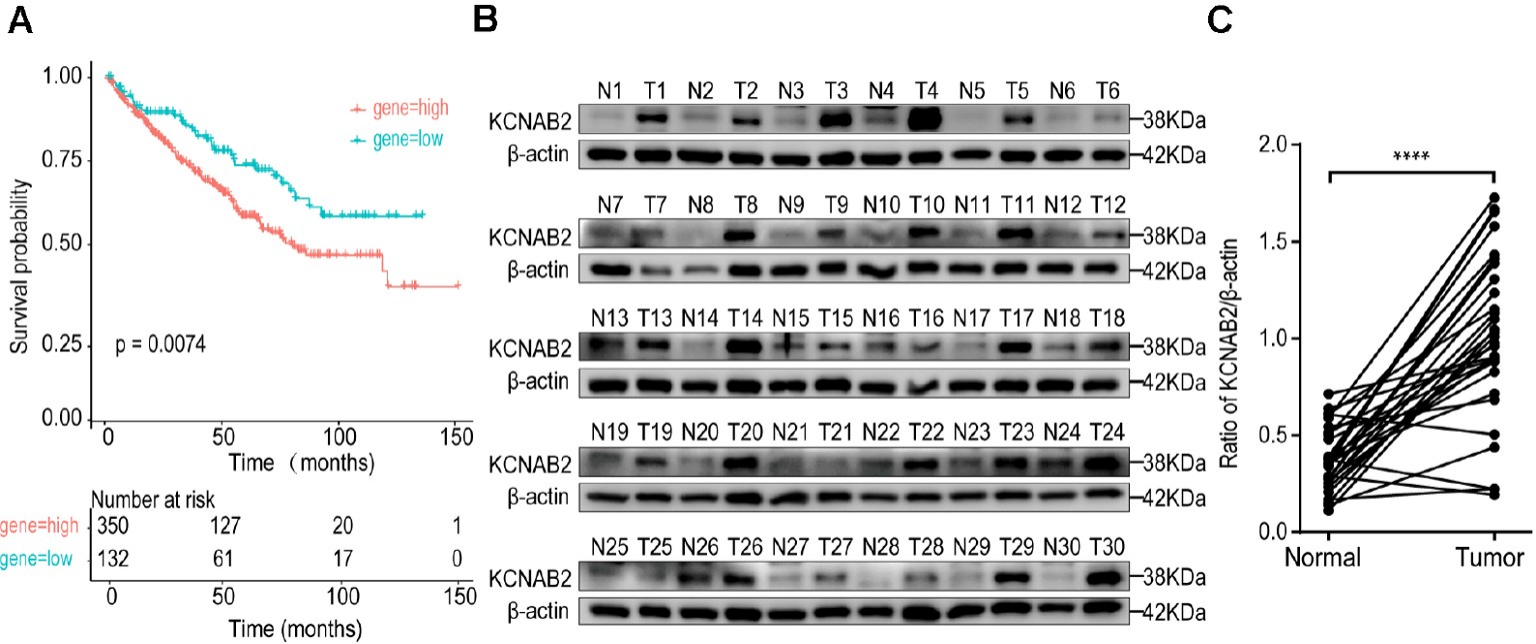
Figure 4 Level of KCNAB2 in RC tissue (A) High KCNAB2 mRNA levels were correlated with unfavorable survival in ccRCC patients. Kaplan‒Meier curves were generated for patients characterized by low and high KCNAB2 levels in the TCGA datasets. Overall survival was expressed as the end point, and the P value was demonstrated. (B) KCNAB2 protein levels in ccRCC tissues and normal tissues measured by western blot analysis. (C) Statistical analysis of KCNAB2 protein levels in tissues.

The correlation between KCNAB2 mRNA levels and the survival of ccRCC patients was assessed using Kaplan‒Meier analysis. Compared to the patients with high KCNAB2 mRNA levels ( *n*=350), the patients with low KCNAB2 mRNA levels ( *n*=132) had longer overall survival times ( *P*=0.0074) in the TCGA data ( Figure 4A). The above results suggested that the expression of KCNAB2 might play a certain role in renal cancer progression. Subsequently, KCNAB2 protein expression in fresh human ccRCC tissues was measured. The expression of KCNAB2 was found at ~38 kDa ( Figure 4B). Furthermore, out of 30 pairs of samples, 27 pairs (90%) had significantly higher levels of KCNAB2 protein in tumor tissues than in matched normal tissues ( Figure 4C).

## Discussion

Although pathological and radiological examinations are still the ″gold standard″ for the clinical diagnosis of cancer, liquid biopsy has been found to have enticing potential for the early detection of RCC. Tremendous efforts have been made for the early diagnostic potential of cell-free DNA methylation in RCC [22]. However, there are no clinical protein biomarkers for the diagnosis of RCC. Existing studies have found a series of protein markers in serum using mass spectrometry. For example, iTRAQ-based quantitative proteomic analysis reported HSC71 as a novel serum biomarker for RCC, and the AUC of HSC71 was 0.86 [23]. Because proteins are unstable, the concentration of proteins secreted into the periphery fluctuates drastically, making their detection results unreliable. Autoantibodies are highly stable in the blood and can be amplified by the immune system. In addition, they are capable of having a long-lasting memory for patients, thus making them ideal biomarkers for diagnosis and prognosis. Therefore, we consider that the protein microarray-based approach may offer a unique advantage. Protein microarray is employed as a preliminary screening tool to discover some candidate biomarkers owing to its high throughput. These selected candidates can be further validated based on ELISA, a common clinical detection method.

Human proteome microarrays have been adopted to screen autoantibodies in the serum of renal cancer patients to discover potential renal cancer biomarkers. In this study, IgG autoantibodies against KCNAB2, GAPDH and OCM were validated by ELISA. Although OCM showed no significant difference between RC and HC, KCNAB2 and GAPDH were nearly consistent with the microarray results. If multiple candidate biomarkers were combined to establish a panel, the diagnostic effect for tumors would be improved [ 14, 15] . We attempted to combine KCNAB2 with GAPDH, and the panel could increase the AUC, instead of improving the specificity and the sensitivity, probably arising from the limited selectable candidates. In the future studies, we will purify more candidates to find more potential RC individual and panel biomarkers. Meanwhile, random forest analysis will provide very important guidance for further verification of candidate biomarkers in future work.

Among the Up autoantibodies, the positive ratios of anti-KCNAB2 IgG, anti-KCNAB1 IgM and anti-KCNAB2 IgM accounted for 56.25%, 56.25% and 50%, respectively. The target proteins KCNAB1 and KCNAB2 were found in potassium channel complexes that could regulate DNA damage by creating reactive oxygen species (ROS). In the Cancer Genome Atlas (TCGA) Kidney Clear Cell Carcinoma Illumina HiSeq data, the KCNAB1 level was found to be significantly correlated with the survival rate of renal cancer patients. The positive ratio of anti-RCN1 IgM accounted for 31.25%, and RCN1 might regulate calcium-dependent activities in the endoplasmic reticulum lumen or post-ER compartment. Western blot analysis, 2D electrophoresis, and mass spectrometry were adopted to confirm that it was highly expressed in renal cancer tissues [24].

Glyceraldehyde-3-phosphate dehydrogenase (GAPDH) has been considered as a reference protein for expression quantification in tumors. In this study, with the use of a proteome microarray, the RC-positive rate of anti-GAPDH IgG was 37.50%, while 36.1% sensitivity and 90.9% specificity were obtained for RC detection by ELISA. Interestingly, it was reported that GAPDH is commonly upregulated in various cancers (e.g., renal cancer) [25].

Anti-CCT8 IgM and anti-CCT3 IgM are Down autoantibodies, and their target proteins CCT8 and CCT3 are components of the chaperonin-containing T-complex (TRiC), a molecular chaperone complex contributing to the folding of proteins upon ATP hydrolysis. The TRiC complex could mediate the folding of WRAP53/TCAB1, thus regulating telomere maintenance [26]. CCT8 has an alternative name of renal carcinoma antigen NY-REN-15, and 65 distinct antigens (NY-REN-1 to NY-REN-65) reactive with autologous IgG were found through SEREX analysis of four renal cancer patients and were characterized according to cDNA sequence, mRNA expression pattern, and reactivity with allogeneic sera [13]. As indicated by this report and our work, CCT8 is a renal cancer antigen, and it was presumed to be a renal cancer-specific antigen in this paper.

Anti-GAB1 IgM, anti-PIK3CA IgM and anti-Crk IgM are Down autoantibodies, and anti-HRAS IgG refers to an Up autoantibody. The bioinformatics analysis suggested that their target proteins were enriched in the MAPK signaling pathway of papillary renal cell carcinoma. GAB1 is prognostic, and high expression of GAB1 is beneficial to the survival of patients with renal cancer (The Human Protein Atlas), thus revealing that the expression of GAB1 is low in renal cancer. PIK3CA was found to have genetic alterations, and amplification or mutations were found in 5% of clear cell renal cell carcinomas [27]. HRAS is a member of the RAS family, and normal or mutated forms of HRAS are overexpressed in various tumors [ 28‒ 31] . QPCT (glutaminyl-peptide cyclotransferase) is bound to HRAS and improves the stability of HRAS by inhibiting its ubiquitination degradation, which can activate the ERK signaling pathway and lead to sunitinib resistance in renal cell cancer [32]. The above results suggested that protein abnormalities in the MAPK signaling pathway might exist in all types of kidney cancer.

This study has some limitations. First, since all proteins on a microarray were homogeneously expressed from normal human protein-coding genes, it is difficult for us to identify autoantibodies to structural changes or abnormal posttranslational modifications of tumor antigens. Second, a few biomarkers were found in this study, and we will further screen and validate more candidate markers in future research with other technology platforms, e.g., mass spectrometry or phage display [33]. Last, the number of serum samples used in the discovery and validation cohorts was relatively small, and the biomarkers found in this study should be validated in larger and diverse cohorts in the future. While we did not evaluate the level of autoantibodies against KCNAB2 and GAPDH in sera of other types of cancer, referring to published results of screening autoantibodies based on proteome microarrays for a variety of tumors, autoantibodies against KCNAB2 and GAPDH seems to be specific in ccRCC. For example, among proteome microarray screening serum biomarkers of HCC, a novel panel containing CIAPIN1, EGFR, MAS1, SLC44A3, ASAH1, UBL7 and ZNF428 was found for effective detection [14]. In lung cancer, p53, ETHE1, CTAG1A, C1QTNF1, TEX264, CLDN2, NSG1 and HRas were found to be the most promising markers [16]. It was therefore revealed that KCNAB2 and GAPDH could be potential RCC-specific biomarkers.

In summary, a systematic method was used to discover serum diagnostic autoantibody biomarkers in RC. A total of 126 candidate autoantibodies (Up) were screened out as a biomarker library of RC. A total of 316 corresponding target proteins of autoantibodies with a significant difference were screened out, including upregulated and downregulated proteins. Anti-KCNAB2-IgG and anti-GAPDH-IgG were confirmed as serum biomarkers for RC diagnosis, and KCNAB2 was confirmed as a biomarker for RC prognosis.

## Supporting information

Supplementary_Table_S1

## Supplementary Data

Supplementary data are available at *Acta Biochimica et Biophysica Sinica* online.

## References

[1] Siegel RL, Miller KD, Fuchs HE, Jemal A (2021). Cancer statistics, 2021. CA Cancer J Clin.

[2] Cancer Stat Facts: Kidney and Renal Pelvis Cancer. vol. 1: National Cancer Institute, 2020.

[3] Perroud B, Lee J, Valkova N, Dhirapong A, Lin PY, Fiehn O, Kültz D (2006). Pathway analysis of kidney cancer using proteomics and metabolic profiling. Mol Cancer.

[4] Castronovo V, Waltregny D, Kischel P, Roesli C, Elia G, Rybak JN, Neri D (2006). A chemical proteomics approach for the identification of accessible antigens expressed in human kidney cancer. Mol Cell Proteomics.

[5] Perroud B, Ishimaru T, Borowsky AD, Weiss RH (2009). Grade-dependent proteomics characterization of kidney cancer. Mol Cell Proteomics.

[6] Atrih A, Mudaliar MAV, Zakikhani P, Lamont DJ, Huang JTJ, Bray SE, Barton G (2014). Quantitative proteomics in resected renal cancer tissue for biomarker discovery and profiling. Br J Cancer.

[7] Yadav S, Kashaninejad N, Masud MK, Yamauchi Y, Nguyen NT, Shiddiky MJA (2019). Autoantibodies as diagnostic and prognostic cancer biomarker: Detection techniques and approaches. Biosens Bioelectron.

[8] Järås K, Anderson K (2011). Autoantibodies in cancer: prognostic biomarkers and immune activation. Expert Rev Proteomics.

[9] Zaenker P, Ziman MR (2013). Serologic autoantibodies as diagnostic cancer biomarkers—a review. Cancer Epidemiol Biomarkers Prevention.

[10] Kaaks R, Fortner RT, Hüsing A, Barrdahl M, Hopper M, Johnson T, Tjønneland A (2018). Tumor-associated autoantibodies as early detection markers for ovarian cancer? A prospective evaluation. Int J Cancer.

[11] Baldin AV, Grishina AN, Korolev DO, Kuznetsova EB, Golovastova MO, Kalpinskiy AS, Alekseev BY (2019). Autoantibody against arrestin-1 as a potential biomarker of renal cell carcinoma. Biochimie.

[12] Kitamura H, Honma I, Torigoe T, Hariu H, Asanuma H, Hirohashi Y, Sato E (2007). Expression of livin in renal cell carcinoma and detection of anti-livin autoantibody in patients. Urology.

[13] Scanlan MJ, Gordan JD, Williamson B, Stockert E, Bander NH, Jongeneel V, Gure AO (1999). Antigens recognized by autologous antibody in patients with renal-cell carcinoma. Int J Cancer.

[14] Zhang S, Liu Y, Chen J, Shu H, Shen S, Li Y, Lu X (2020). Autoantibody signature in hepatocellular carcinoma using seromics. J Hematol Oncol.

[15] Ling HZ, Xu SZ, Leng RX, Wu J, Pan HF, Fan YG, Wang B (2020). Discovery of new serum biomarker panels for systemic lupus erythematosus diagnosis. Rheumatology.

[16] Pan J, Song G, Chen D, Li Y, Liu S, Hu S, Rosa C (2017). Identification of serological biomarkers for early diagnosis of lung cancer using a protein array-based approach. Mol Cell Proteomics.

[17] Yang L, Wang J, Li J, Zhang H, Guo S, Yan M, Zhu Z (2016). Identification of serum biomarkers for gastric cancer diagnosis using a human proteome microarray. Mol Cell Proteomics.

[18] Qi H, Ma M, Lai D, Li Y, Zhang F, Tao S (2021). Assessment and comparison of recombinant proteins from different sources for the detection of SARS-CoV-2 infection by using protein microarray. Acta Biochim Biophys Sin.

[19] Xu YW, Peng YH, Chen B, Wu ZY, Wu JY, Shen JH, Zheng CP (2014). Autoantibodies as potential biomarkers for the early detection of esophageal squamous cell carcinoma. Am J Gastroenterol.

[20] Liu C, Wu F, Jiang H, He X, Guo S, Tao S (2014). Global identification of CobB interactors by an
*Escherichia coli* proteome microarray. Acta Biochim Biophys Sin.

[21] Fitzgerald S, O′Reilly JA, Wilson E, Joyce A, Farrell R, Kenny D, Kay EW (2019). Measurement of the IgM and IgG autoantibody immune responses in human serum has high predictive value for the presence of colorectal cancer. Clin Colorectal Cancer.

[22] Nuzzo PV, Berchuck JE, Korthauer K, Spisak S, Nassar AH, Abou Alaiwi S, Chakravarthy A (2020). Detection of renal cell carcinoma using plasma and urine cell-free DNA methylomes. Nat Med.

[23] Zhang Y, Cai Y, Yu H, Li H (2015). iTRAQ-based quantitative proteomic analysis identified HSC71 as a novel serum biomarker for renal cell carcinoma. Biomed Res Int.

[24] Giribaldi G, Barbero G, Mandili G, Daniele L, Khadjavi A, Notarpietro A, Ulliers D (2013). Proteomic identification of Reticulocalbin 1 as potential tumor marker in renal cell carcinoma. J Proteomics.

[25] Guo C, Liu S, Sun MZ (2013). Novel insight into the role of GAPDH playing in tumor. Clin Transl Oncol.

[26] Freund A, Zhong FL, Venteicher AS, Meng Z, Veenstra TD, Frydman J, Artandi SE (2014). Proteostatic control of telomerase function through TRiC-mediated folding of TCAB1. Cell.

[27] Creighton CJ, Morgan M, Gunaratne PH, Wheeler DA, Gordon Robertson A, Beroukhim R, Vandin Hsin-Ta Wu F (2013). Comprehensive molecular characterization of clear cell renal cell carcinoma. Nature.

[28] Geyer FC, Li A, Papanastasiou AD, Smith A, Selenica P, Burke KA, Edelweiss M (2018). Abstract PD4-13: Estrogen receptor-negative breast adenomyoepitheliomas are driven by co-occurring
*HRAS* hotspot and PI3K pathway gene mutations: A genetic and functional analysis. Cancer Res.

[29] Sugita S, Enokida H, Yoshino H, Miyamoto K, Yonemori M, Sakaguchi T, Osako Y (2018). HRAS as a potential therapeutic target of salirasib RAS inhibitor in bladder cancer. Int J Oncol.

[30] Arnault JP, Mateus C, Escudier B, Tomasic G, Wechsler J, Hollville E, Soria JC (2012). Skin Tumors Induced by Sorafenib; Paradoxic RAS–RAF Pathway Activation and Oncogenic Mutations of
*HRAS* ,
*TP53* , and
*TGFBR1*. Clin Cancer Res.

[31] Topf MC, Wang ZX, Tuluc M, Pribitkin EA (2018). *TERT* ,
*HRAS* , and
*EIF1AX* Mutations in a Patient with Follicular Adenoma. Thyroid.

[32] Zhao T, Bao Y, Gan X, Wang J, Chen Q, Dai Z, Liu B (2019). DNA methylation-regulated QPCT promotes sunitinib resistance by increasing HRAS stability in renal cell carcinoma. Theranostics.

[33] Qi H, Ma M, Lai D, Tao S (2021). Phage display: an ideal platform for coupling protein to nucleic acid. Acta Biochim Biophys Sin.

