# Supplementary material for: Screening of autoantibodies as biomarkers in the serum of renal cancer patients based on human proteome microarray: Screening of autoantibodies in renal cancer
Source: Acta Biochim Biophys Sin (Shanghai). 2022 Dec 23;54(12):1909–16. doi: 10.3724/abbs.2022189 (PMC10157637; doi:10.3724/abbs.2022189)
Supplement: Supplementary_Table_S1 [file Supplementary_Table_S1.pdf]

Fold change (FC), the ratio of the mean value of RC to HC.
The optimal cutoff value for each candidate biomarker was evaluated with two criteria1) at least 90% specificity
The screening criteria of Up: RC positive ratio > 30%, HC positive ratio < 10%, FC ≥ 1.5 and the p-value of T-te
The screening criteria of Down: HC positive ratio > 30%, RC positive ratio < 10%, FC < 2/3 and the p-value of T

ID	Name	Type	Result	RC1	RC2	RC3	RC4	RC5	RC6
JHU07151	KCNAB2	IgG	Up	11.52394	15.6181	59.32107	37.73978	24.2915	32.44069
JHU02029	CALR	IgG	Up	4.30884	2.204564	1.799299	2.194486	2.611047	1.556103
JHU07995	IFI27L2	IgG	Up	1.790412	7.369577	6.010726	2.521709	5.84546	1.368067
JHU18162	PRR27	IgG	Up	3.415	1.946053	30.41156	4.405166	2.720847	1.918519
JHU11118	GEMIN2	IgG	Up	1.158912	2.247689	6.015384	2.187635	2.329971	1.67525
JHU00409	CIRBP	IgG	Up	1.969882	2.434757	8.625781	5.024563	3.121327	4.52566
JHU15238	KHDRBS1	IgG	Up	3.301506	2.448228	3.68772	5.345986	3.252588	5.293337
JHU25652	CSPG4	IgG	Up	1.108268	0.709239	4.890204	1.368108	1.550985	0.792108
JHU10807	RAB33B	IgG	Up	1.205053	0.757395	4.305593	1.115314	2.251839	0.792851
JHU03006	CYB5R3	IgG	Up	1.376017	5.186158	3.347658	3.183361	3.206902	73.29671
JHU10093	BLOC1S4	IgG	Up	22.90131	9.896577	6.123696	4.34696	6.427825	60.74941
JHU13578	TOR1AIP2	IgG	Up	1.191939	0.719377	6.728684	2.17769	2.156129	1.132521
JHU25143	WIPF1	IgG	Up	5.461924	2.51737	16.06542	6.405727	4.85934	6.868448
JHU17076	OCM	IgG	Up	0.93006	0.502753	10.68723	1.375802	1.422923	0.851048
JHU17126	C1orf127	IgG	Up	2.737749	1.658135	54.89618	7.914126	5.743923	2.181333
JHU03930	TMEM44	IgG	Up	5.797554	13.80653	9.059358	7.194028	71.59441	18.23588
JHU07466	PRRC1	IgG	Up	1.345535	2.872973	63.79708	15.88271	24.85313	5.091032
JHU15484	BCAS4	IgG	Up	1.344269	1.237463	34.7173	6.658507	7.52347	0.917239
JHU07150	KCNAB2	IgG	Up	5.012278	7.991495	16.11778	20.55891	10.43966	18.99618
JHU17247	TBC1D15	IgG	Up	1.813216	2.803132	4.845512	1.855814	2.816777	1.939909
JHU11568	MYOD1	IgG	Up	2.412696	6.954343	3.496113	9.64819	6.39118	16.29405
JHU16988	RBM47	IgG	Up	1.316055	1.906932	9.510623	4.772441	3.463304	3.152774
JHU00973	BNIP3L	IgG	Up	2.640203	4.285546	3.047255	34.71529	2.284061	2.232725
JHU00787	CAAP1	IgG	Up	3.329507	20.19883	12.38656	2.823001	7.69354	2.079988
JHU05130	LECT1	IgG	Up	2.734997	1.65702	5.704905	2.324581	1.714241	1.148381
JHU11855	NAP1L5	IgG	Up	1.946089	1.50577	7.59192	5.353991	3.957693	7.012445
JHU13661	RPS6KA1	IgG	Up	1.769624	3.084849	2.931834	2.725332	6.569435	10.18148
JHU15073	RBPMS2	IgG	Up	4.39924	5.284553	6.082425	3.815998	3.980844	4.232481
JHU05252	PSMA1	IgG	Up	26.74282	6.236375	4.263875	2.11147	3.651231	3.847191
JHU02752	PCNA	IgG	Up	2.795031	2.294975	4.162102	3.27717	2.539286	2.553242
JHU10781	ND	IgG	Up	0.717428	0.86839	8.179828	2.58209	3.432447	0.860714
JHU02626	GSTO1	IgG	Up	3.209654	3.626862	12.77805	2.98243	3.010002	8.312523
JHU11235	ANKRD45	IgG	Up	1.504252	4.565516	12.35137	67.31952	2.786231	11.23249
JHU28292	SP3	IgG	Up	0.749011	1.024607	8.905169	1.570638	1.612775	1.102268
JHU17128	CASK	IgG	Up	6.000658	1.093505	18.85827	1.741391	5.135149	1.216043
JHU00420	GAPDH	IgG	Up	3.124849	4.130257	14.96591	5.112153	2.849274	4.241518
JHU17493	SPANXN2	IgG	Up	3.661125	26.41697	33.70817	7.897557	20.63531	7.405698
JHU07989	YBX3	IgG	Up	4.664908	5.972637	5.609535	6.644016	4.182306	9.95266
JHU19985	Supt6h	IgG	Up	63.65036	9.505249	86.01646	2.695905	26.6744	5.598941
JHU17245	TACC1	IgG	Up	0.860435	0.716098	8.249016	2.254874	3.900691	0.646006
JHU28073	HECTD3	IgG	Up	1.312296	9.155138	4.102757	1.344618	2.796155	1.569348
JHU09466	OBFC1	IgG	Up	2.992909	1.560701	7.988834	3.584074	4.041714	2.649196
JHU13223	CIB1	IgG	Up	1.512011	0.683238	12.46528	4.665206	4.479293	0.77771
JHU18375	KCNK1	IgG	Up	6.16502	1.628456	28.44864	1.295811	1.680788	5.718932
JHU13200	P3H4	IgG	Up	2.340217	2.636779	4.954869	1.914796	7.114065	2.646829
JHU04186	GAR1	IgG	Up	2.04184	2.564115	3.646955	5.435477	4.317691	4.295624
JHU02341	FTL	IgG	Up	2.846471	4.775586	12.12534	6.151896	3.910288	2.840349

JHU09086	PCMTD1	IgG	Up	3.794524	13.10873	28.43541	14.30997	18.77188	33.11211
JHU11130	NG_03030	IgG	Up	1.408408	2.345787	10.67348	8.001056	5.075118	6.57021
JHU26730	CFHR3	IgG	Up	0.890046	3.315762	5.777121	1.763243	2.178244	1.382391
JHU02864	TSN	IgG	Up	1.79399	3.136817	8.613552	6.347146	2.187431	1.823454
JHU17403	TXLNA	IgG	Up	1.803488	2.837967	24.25081	3.145655	2.545319	8.302452
JHU08331	RCN1	IgG	Up	4.733945	2.534509	4.574278	1.379686	18.44423	1.404437
JHU27170	HSD17B14	IgG	Up	1.133333	4.524188	25.70806	2.907554	4.633844	1.464053
JHU00738	RBMS1	IgG	Up	1.444761	2.582764	3.382848	5.610023	3.664758	6.311125
JHU12551	BORA	IgG	Up	3.731979	4.754587	4.747293	2.483408	3.487677	1.869475
JHU11010	PSMA1	IgG	Up	8.111689	7.76387	3.926079	1.604812	5.049452	3.17139
JHU04143	CEP112	IgG	Up	2.3814	7.189588	2.56471	3.64936	3.981569	4.588925
JHU14259	YBX2	IgG	Up	2.029883	3.590371	5.684817	6.056506	4.584866	6.303447
JHU15425	LETM2	IgG	Up	5.128462	2.789193	9.325625	3.110519	5.904802	7.611352
JHU00996	FAM131C	IgG	Up	35.56096	8.000044	22.56128	7.492043	70.40351	31.85531
JHU16911	SULT1A3	IgG	Up	1.253687	1.041951	6.96589	1.32608	1.9665	0.825492
JHU00667	USP15	IgG	Up	0.816009	2.608617	37.84183	11.82703	11.32906	1.490672
JHU12430	TOM1L2	IgG	Up	24.16508	9.278505	20.62214	4.624117	78.3224	3.151306
JHU09069	RALYL	IgG	Up	2.830094	7.260395	15.68732	9.643985	16.31109	7.634929
JHU25614	NES	IgG	Up	6.065244	24.59081	6.883353	6.684518	6.444142	4.638854
JHU16919	VAT1	IgG	Up	1.83499	1.721543	61.65146	6.824901	10.21978	1.85932
JHU02987	FAM212B	IgG	Up	2.082033	1.725409	9.467842	2.64014	3.190348	1.363513
JHU04297	RFC2	IgG	Up	1.015498	0.745831	4.479425	1.430116	1.713256	0.93375
JHU07166	PTPN5	IgG	Up	3.090431	2.424987	16.17559	2.448027	20.57519	6.88295
JHU07839	RBMS1	IgG	Up	1.674137	4.313619	3.682069	6.492446	4.559844	5.277653
JHU06542	EEF2K	IgG	Up	7.177393	7.543334	33.56599	33.63258	27.43942	96.92613
JHU11111	RBPJ	IgG	Up	9.233323	17.87582	100.2878	117.4003	72.78744	85.06664
JHU27744	COA1	IgG	Up	1.500445	1.947962	5.982298	1.873927	1.736225	1.309565
JHU01319	PSMA1	IgG	Up	23.2333	8.214768	5.280601	2.513102	5.238937	8.283225
JHU11739	ECE1	IgG	Up	21.41678	8.224603	3.244255	2.723547	23.62525	7.421852
JHU13192	QDPR	IgG	Up	1.210644	1.670217	3.845112	3.743886	1.978145	2.208174
JHU16908	RSPH1	IgG	Up	1.220683	1.553615	75.75478	11.06992	17.93998	1.018786
JHU10885	CARD18	IgG	Up	1.772319	10.45506	7.237993	3.404762	8.246429	2.883462
JHU01606	PSMC4	IgG	Up	1.366684	0.91881	11.74113	2.536625	3.552602	1.312328
JHU10767	EIF2C3	IgG	Up	0.567228	0.693126	14.99098	3.830488	7.539637	0.786239
JHU03792	LGALS2	IgG	Up	2.602264	7.845048	7.711593	2.061948	4.669675	3.501273
JHU13413	CAAP1	IgG	Up	1.776616	9.890809	4.519212	1.833963	4.84335	1.263308
JHU16261	CCDC6	IgG	Up	2.930944	5.703746	5.037875	3.519872	10.82	86.25276
JHU16024	PRB3_frag	IgG	Up	12.13238	3.93836	3.568319	3.843288	46.67001	4.097034
JHU03468	BAG6	IgG	Up	15.73	4.520428	9.164463	5.475359	17.16886	12.54118
JHU10201	CYB5D1	IgG	Up	1.324251	2.144268	4.724469	1.559001	7.505732	3.575301
JHU17742	OCM2	IgG	Up	0.965711	0.581873	43.85682	1.543477	1.696762	0.981489
JHU00581	ARL2BP	IgG	Up	1.345619	1.65249	10.20104	3.006887	2.364029	1.852226
JHU17128	CASK	IgG	Up	3.364435	1.177114	32.20714	1.802019	6.595414	1.011583
JHU27398	SHC1	IgG	Up	3.349975	12.93903	13.87146	4.17063	10.65771	3.705325
JHU03607	NME5	IgG	Up	16.39842	3.668106	2.830747	2.064988	3.037139	1.709092
JHU10755	ASTE1	IgG	Up	0.613514	0.654312	7.41819	2.025526	4.507986	0.69271
JHU14076	ADPGK	IgG	Up	1.79522	2.588275	4.498536	1.498102	7.228826	52.28886
JHU09151	FTL	IgG	Up	2.764277	6.215504	21.18846	12.07162	5.905756	4.142389
JHU03691	HAAO	IgG	Up	2.265539	1.901944	2.840851	1.697231	2.439111	11.18572
JHU01009	KPNA6	IgG	Up	0.858588	0.783132	6.127088	1.292131	2.530699	0.869049
JHU09087	PRKCSH_	IgG	Up	2.378304	4.551615	18.53409	9.133209	31.00841	6.074565
JHU16866	HSPB3	IgG	Up	3.956089	4.318893	9.832957	2.800576	5.362537	2.816101
JHU17327	ARHGEF2	IgG	Up	1.203233	1.179224	5.129266	1.917457	1.236489	1.467471
JHU04079	HSPA1L	IgG	Up	1.750622	3.46631	5.355486	3.544354	4.37263	5.719597

JHU04186	GAR1	IgM	Up	6.617545	3.444963	6.507644	3.215746	6.339423	4.837277
JHU05510	KCNAB1	IgM	Up	14.1029	12.05735	27.24217	19.06447	9.843922	27.29445
JHU07150	KCNAB2	IgM	Up	37.90021	26.88532	73.18368	38.2257	50.38975	75.82319
JHU11182	KCNAB1	IgM	Up	6.282228	5.609051	11.73251	3.706585	7.677857	10.06704
JHU04457	MPG	IgM	Up	8.498174	4.233532	10.64009	9.236386	5.556386	7.279475
JHU07064	MRPS31	IgM	Up	10.61442	5.561975	14.6803	37.84911	15.74665	8.057038
JHU07902	FFAR2	IgM	Up	5.099294	6.26463	11.38613	10.95145	13.16196	5.425058
JHU08331	RCN1	IgM	Up	3.936196	9.202403	9.596546	5.008097	26.0816	6.03277
JHU08486	LUC7L2	IgM	Up	28.17945	4.595291	11.03345	5.560088	16.45819	7.432076
JHU13665	SCCPDH	IgM	Up	8.717937	3.328256	5.840069	4.67609	6.855574	7.222179
JHU13729	F2	IgM	Up	18.17022	11.05239	26.03278	20.8136	22.13028	22.66699
JHU04449	HNRNPC	IgM	Up	13.28846	6.532346	6.165104	7.618716	37.50947	8.564896
JHU07151	KCNAB2	IgM	Up	59.42785	46.03865	169.6301	72.13549	108.1576	93.79116
JHU13646	MTERF4	IgM	Up	29.75989	22.6847	92.49101	13.35786	37.50952	53.14009
JHU13741	KCNAB1	IgM	Up	39.64098	25.79158	85.00468	39.29576	34.33841	56.47011
JHU14956	MYLK	IgM	Up	10.17198	1.08233	48.73955	2.162718	18.11586	19.57137
JHU15238	KHDRBS1	IgM	Up	7.915052	3.622385	6.305214	4.150072	2.903769	4.310462
JHU16355	KCTD9	IgM	Up	4.819799	6.223866	14.40873	16.5684	19.3371	9.658896
JHU18741	B3GNT7	IgM	Up	12.78483	4.232504	8.35069	17.69874	4.341072	5.960526
JHU19216	MMP21	IgM	Up	5.954311	2.80783	9.831346	7.52256	14.66629	5.539117
JHU08252	ZADH2	IgM	Up	7.785345	5.041861	23.90286	6.999125	9.953779	15.89509
JHU11011	R3HDM2_	IgM	Up	3.158053	3.116624	3.364924	10.30153	5.828499	2.720229
JHU01715	SRP19	IgM	Up	5.517973	6.79782	8.849866	42.22788	10.91488	4.763414
JHU15555	R3HDM2_	IgM	Up	3.715503	3.711554	5.732173	19.11486	14.29618	3.483435
JHU16428	BC073767	IgM	Up	28.85603	18.99707	141.8725	48.23735	45.27421	64.97878
JHU00030	GAP43	IgG	Down	1.424606	2.284463	2.812433	2.281019	2.749567	2.185713
JHU00319	HN1	IgG	Down	0.898108	0.763687	1.478572	0.915346	6.939062	3.852817
JHU00323	IFIT3	IgG	Down	3.691761	5.609295	3.059962	4.113145	4.320485	6.277706
JHU01550	IFT46	IgG	Down	1.534078	1.943658	0.938297	1.682119	2.788201	1.199699
JHU01742	CCER1	IgG	Down	0.922476	2.737388	2.208564	1.431925	2.175219	1.386399
JHU03511	LSM6	IgG	Down	1.483741	5.52905	10.11358	3.342904	6.268181	3.298751
JHU03795	CROCCP2	IgG	Down	1.329041	4.271646	2.043359	2.225479	2.939965	1.709043
JHU06009	CROCCP2	IgG	Down	2.330557	3.356685	1.63703	3.573015	2.38549	1.596683
JHU06371	GABRE	IgG	Down	1.47538	6.544554	2.063385	0.808841	5.467308	1.441524
JHU07181	SNAP91	IgG	Down	1.270553	2.36221	2.271067	2.028807	6.401368	1.553876
JHU07511	EME1	IgG	Down	0.979636	1.336709	9.428552	1.50868	1.163178	1.211914
JHU07975	TEX29	IgG	Down	1.891169	3.48021	2.925549	2.044121	2.565961	1.620648
JHU08407	MGRN1	IgG	Down	0.895237	2.654932	1.421761	1.509474	2.887494	1.214902
JHU08611	RBM12	IgG	Down	9.827583	19.43672	6.400047	5.132098	2.641123	6.092338
JHU09533	FCRL2	IgG	Down	1.12433	3.525144	1.260077	1.344842	1.195806	17.83277
JHU11977	TFAP2D	IgG	Down	2.08408	5.149834	11.74998	3.282526	4.983223	3.577485
JHU13532	HEMGN	IgG	Down	0.734678	3.364373	8.411487	1.457052	11.85918	3.876782
JHU13781	USP48	IgG	Down	1.311015	6.07758	3.964589	4.157786	3.787449	6.343663
JHU16239	SRPK2	IgG	Down	1.664934	2.830461	3.280769	2.11584	2.68023	2.992105
JHU16417	HDAC6	IgG	Down	1.53462	3.959429	2.431321	1.97153	2.632198	1.627577
JHU16600	AC145212	IgG	Down	1.65057	5.327624	2.250106	3.363607	3.075543	2.928946
JHU00022	COL4A3BI	IgM	Down	20.01001	5.6673	30.22361	28.2094	42.62749	20.82404
JHU00023	CRK	IgM	Down	12.08974	13.9793	5.342487	11.81516	11.12615	11.07815
JHU00030	GAP43	IgM	Down	3.406623	14.31952	5.409969	9.264926	1.729768	8.934178
JHU00117	CMPK1	IgM	Down	2.457045	3.123969	1.33635	3.119786	1.643356	1.926531
JHU00249	PRPSAP1	IgM	Down	6.763313	6.203126	6.827622	5.490966	8.382838	3.7312
JHU00307	CIAO1	IgM	Down	4.610137	3.781693	2.498671	4.101636	5.838304	2.289446
JHU00323	IFIT3	IgM	Down	8.15138	8.424522	19.66535	14.74584	17.36393	8.422601
JHU00656	SELENBP	IgM	Down	6.749363	7.621252	98.68467	7.528347	11.55722	4.23236

JHU01173	EMILIN1	IgM	Down	4.649818	4.081209	3.983113	7.139481	7.165966	11.03418
JHU01215	RUNDC3A	IgM	Down	4.757731	6.911891	14.26014	6.211768	8.614464	7.724517
JHU01218	RWDD1	IgM	Down	8.657293	26.19202	14.61544	18.73156	20.05399	16.46706
JHU01290	FXYD5	IgM	Down	3.680095	6.291998	5.802765	6.467835	11.10542	3.747017
JHU01391	IQWD1	IgM	Down	2.756337	4.058323	6.179577	2.764127	3.856294	9.094825
JHU01577	FDPS	IgM	Down	5.483365	7.039974	6.895673	7.769678	8.660607	4.67944
JHU01720	TGOLN2	IgM	Down	5.071389	3.38436	1.627383	6.421083	3.063783	2.201448
JHU01787	MARC2	IgM	Down	5.739904	6.327186	2.650668	5.838179	10.78333	4.870786
JHU01858	FGL2	IgM	Down	12.75556	4.877405	3.568751	4.458858	20.12687	8.704749
JHU02111	XRCC4	IgM	Down	14.31626	17.59832	16.2304	5.620123	13.65589	11.91412
JHU02124	CCT3	IgM	Down	10.92761	9.761256	11.05899	11.73164	19.63774	9.832589
JHU02290	TEX264	IgM	Down	3.397535	9.528397	7.868627	4.972161	6.497103	3.272629
JHU02469	RBBP4	IgM	Down	4.350397	7.771559	3.887257	2.951709	3.933884	1.910926
JHU02553	NASP	IgM	Down	12.6512	9.796439	14.11522	4.370541	6.958066	5.344474
JHU02594	AHSA1	IgM	Down	10.52935	8.930582	9.296679	6.08602	14.21511	10.58817
JHU02616	DCTN3	IgM	Down	7.563069	6.108369	4.92087	5.123914	11.74507	4.097761
JHU02653	PEX19	IgM	Down	5.089954	7.61097	5.893318	3.801251	3.498648	3.781283
JHU02685	DCTPP1	IgM	Down	17.55045	13.46475	21.37603	26.86818	24.0643	13.29765
JHU02742	MAGEA8	IgM	Down	19.26939	10.12338	20.69005	11.4247	16.95791	13.62112
JHU02763	RNH1	IgM	Down	5.629085	6.265874	2.496722	6.890536	14.48791	5.127738
JHU02812	GADD45G	IgM	Down	15.36395	18.04905	11.73328	14.39978	23.00512	14.20928
JHU02818	HSPBP1	IgM	Down	6.627375	14.2121	8.088011	11.47518	12.85933	5.667685
JHU02843	PSMD4	IgM	Down	9.024921	6.615981	11.13954	10.73113	7.079653	6.134429
JHU02929	MRFAP1L	IgM	Down	26.62532	14.26603	13.75851	12.78928	25.44124	14.86518
JHU02965	TRMT12	IgM	Down	6.491566	3.439031	3.388102	7.073262	6.333754	3.860257
JHU03451	XAGE2	IgM	Down	41.25605	29.99196	18.81084	11.34918	24.32652	20.7334
JHU04112	SHCBP1	IgM	Down	35.72408	22.81757	24.76412	14.72857	21.59261	10.20033
JHU04152	CTTN	IgM	Down	6.520649	4.625374	9.83025	4.336346	1.827493	2.939601
JHU04439	EIF2S1	IgM	Down	3.459761	4.250569	6.170369	9.259433	7.321345	2.924001
JHU04592	MED21	IgM	Down	9.645133	9.849127	8.435759	7.886889	16.59461	11.52423
JHU04604	TXNDC17	IgM	Down	8.9456	7.514654	6.098382	10.58592	16.0101	2.95815
JHU04765	RAD23B	IgM	Down	9.873328	11.54356	16.58677	11.1961	17.57287	8.452318
JHU04773	SERPINB2	IgM	Down	16.44193	18.54955	8.002015	12.75731	30.5307	11.01418
JHU04794	URM1	IgM	Down	18.49649	14.09491	6.10305	17.55336	24.55804	22.44207
JHU04918	DBNDD2	IgM	Down	5.182391	4.307428	4.007554	3.061891	6.949002	8.127317
JHU04946	MEA1	IgM	Down	13.99907	5.064772	7.881817	4.448322	1.578739	1.795937
JHU05195	CALML5	IgM	Down	6.211392	11.94118	2.352274	3.223243	7.836772	14.69692
JHU05224	HDGF	IgM	Down	2.210359	2.86414	4.025761	2.874673	1.597173	3.171477
JHU05289	RNF219	IgM	Down	2.088445	4.631462	4.500713	2.797472	4.457515	3.240963
JHU05301	CDKN2AIF	IgM	Down	2.548626	2.403291	4.856807	2.980211	1.946255	3.155492
JHU05316	GUCA1A	IgM	Down	3.629695	3.792122	2.197572	2.742012	7.170479	2.143963
JHU05526	PEPD	IgM	Down	9.825961	7.312463	6.931043	7.369234	7.754736	8.071493
JHU05577	C4orf19	IgM	Down	20.31883	23.49593	27.43037	8.798193	15.72321	17.45949
JHU05581	CCDC89	IgM	Down	11.37206	23.12195	19.07477	11.48265	16.57586	13.87341
JHU05677	EAF1	IgM	Down	19.80322	10.37508	22.4048	50.65635	18.14912	24.27221
JHU06319	RGS19	IgM	Down	5.759076	5.941456	1.821899	3.27111	2.728417	2.802269
JHU06468	TLDC1	IgM	Down	3.574593	5.326142	2.404672	1.95464	3.08559	2.813367
JHU06529	BID	IgM	Down	11.8696	13.2085	6.843227	12.12743	27.92524	19.64257
JHU06558	MAPK9	IgM	Down	23.00477	42.00033	23.30347	10.48285	16.80747	12.53535
JHU06686	RMI1_frag	IgM	Down	3.245168	6.855657	25.4147	10.43335	3.116265	8.373758
JHU06687	SERPINB1	IgM	Down	10.38815	8.235251	10.5615	8.330724	17.65661	23.89121
JHU06709	ZCCHC12	IgM	Down	3.766807	6.272966	3.831176	3.787108	2.58257	2.866983
JHU06779	PAGE2	IgM	Down	4.971232	8.710473	3.692781	5.488404	3.099094	4.53308
JHU06895	SGIP1	IgM	Down	4.940386	17.2422	17.85537	41.94674	12.42652	9.881779

JHU06895	SGIP1	IgM	Down	4.246825	8.88871	16.11557	31.67941	8.848066	7.483326
JHU06931	CSNK1A1l	IgM	Down	4.057542	3.149105	3.742725	3.068	3.176221	2.452285
JHU07020	ACAP2	IgM	Down	6.766422	7.375054	3.807725	11.26556	13.99755	5.945664
JHU07049	IRS1	IgM	Down	3.146101	5.506111	8.812829	4.92783	13.78677	8.82938
JHU07061	MAPK8	IgM	Down	8.640384	7.988662	11.86249	5.477632	12.24269	21.75042
JHU07156	MAPK10	IgM	Down	10.56536	27.11683	9.475419	9.86717	20.62031	18.7937
JHU07170	RPUSD2	IgM	Down	17.24436	42.29422	11.11836	42.45212	11.1627	40.53633
JHU07181	SNAP91	IgM	Down	3.129376	2.880971	1.351808	2.311813	3.179404	1.174826
JHU07181	SNAP91	IgM	Down	5.915771	7.94111	3.927595	10.43478	1.43095	2.337135
JHU07233	GSTA1	IgM	Down	37.78265	19.62844	15.52158	25.08463	26.43984	16.57197
JHU07237	HP	IgM	Down	6.088496	5.657558	4.962593	4.798874	6.842317	3.779603
JHU07250	FO680690	IgM	Down	5.179407	4.078397	5.95538	3.479799	6.420297	2.914938
JHU07273	RAB27A	IgM	Down	19.10978	12.47247	27.40092	10.39956	34.50278	12.28486
JHU07330	DDIAS	IgM	Down	16.66546	11.86244	15.73017	26.02707	18.54956	27.24475
JHU07452	NDRG1	IgM	Down	16.2087	24.73722	95.57239	21.5339	29.39758	11.08068
JHU07469	RCSD1	IgM	Down	14.30367	6.567437	24.40441	13.00214	13.65393	8.785274
JHU07612	FUT9	IgM	Down	8.288429	4.726458	5.06478	5.515061	19.13081	4.331527
JHU07799	DIABLO	IgM	Down	12.07567	16.53865	20.22122	26.34253	19.67584	12.43574
JHU07838	RAD51D	IgM	Down	7.592247	2.469989	2.142415	2.78764	4.267845	2.150034
JHU07975	TEX29	IgM	Down	2.803456	4.875818	7.308795	8.854179	4.676472	2.474013
JHU07981	CCNG2	IgM	Down	1.401638	2.281332	5.646612	4.581643	2.675335	1.554096
JHU08036	KJ901215	IgM	Down	18.29231	20.40788	14.00367	11.69602	49.11681	12.89148
JHU08154	DCAF8	IgM	Down	2.505221	2.518369	30.86806	2.55767	3.649844	6.348631
JHU08244	TRAP1	IgM	Down	4.079515	6.637472	4.018437	5.268696	8.478009	4.573127
JHU08317	OGFR	IgM	Down	19.07787	15.14856	19.92696	9.968997	7.293753	4.669356
JHU08337	SERPINB4	IgM	Down	3.859638	4.308314	3.585442	3.443834	4.946067	2.304515
JHU08569	DDB1	IgM	Down	3.585349	5.268622	0.350442	8.027474	1.349638	0.246828
JHU08658	COL14A1	IgM	Down	8.504845	7.43442	6.604855	6.553544	6.686047	6.157403
JHU08835	ATP1A3	IgM	Down	7.200778	25.7374	14.56988	15.65827	2.543161	6.513712
JHU08890	NASP	IgM	Down	7.836312	21.00423	16.43823	16.87201	3.394858	7.179736
JHU08894	OGFOD1	IgM	Down	7.517368	12.11307	3.748753	3.877312	1.927282	3.04409
JHU08970	MAGEA9	IgM	Down	10.02264	10.34721	13.16828	6.08304	29.8937	6.829898
JHU09320	LBHD1	IgM	Down	2.607891	3.103351	3.482125	2.745557	3.951603	3.725884
JHU09332	COL23A1	IgM	Down	1.959648	2.379395	2.767998	1.369088	2.788917	3.43238
JHU09535	GAB1	IgM	Down	10.61204	14.12234	15.72209	8.127572	4.64849	17.99478
JHU09535	GAB1	IgM	Down	8.422553	7.546295	10.7588	4.780783	2.405556	9.014049
JHU09618	CCT8	IgM	Down	6.837162	19.18399	10.12021	8.953615	10.05346	12.40498
JHU09621	DAB1	IgM	Down	17.13182	20.09043	22.5826	17.03911	18.75833	28.09489
JHU09712	COQ9	IgM	Down	2.518972	5.388401	1.639998	3.024932	3.607362	1.499166
JHU10086	C1orf174	IgM	Down	4.88782	5.779593	9.112764	8.854802	7.278778	4.508912
JHU10095	DAB1	IgM	Down	5.864099	8.370393	12.4884	10.59629	4.595445	14.20499
JHU10332	PDXDC1	IgM	Down	3.979433	3.775895	4.032504	2.803208	4.470523	3.937041
JHU10582	CNNM1	IgM	Down	5.505746	18.45804	6.151254	23.59736	11.99642	13.55255
JHU10585	DIABLO	IgM	Down	6.06534	6.631955	26.26995	18.7963	12.92401	7.130492
JHU10643	TSNAXIP1	IgM	Down	6.513052	12.00371	11.22538	9.050239	11.8274	9.923293
JHU10895	ERICH2	IgM	Down	8.298272	12.48496	30.60462	9.112726	4.414909	20.91701
JHU10995	NACA	IgM	Down	3.452037	3.09522	15.5588	2.820448	1.873444	2.389239
JHU11495	PUF60	IgM	Down	7.857303	8.489194	11.07982	7.213943	11.5199	8.060145
JHU11534	CCT8	IgM	Down	2.282801	8.854905	3.925232	4.059901	5.339238	4.866564
JHU11834	GRPEL2	IgM	Down	4.566288	4.439956	2.490422	3.08107	6.444148	3.572
JHU12041	SMOX	IgM	Down	4.129915	7.336662	5.250005	8.355148	8.920017	11.32859
JHU12202	HNMT	IgM	Down	7.748521	18.25541	9.294041	16.66224	41.36719	18.25955
JHU12356	C12orf10	IgM	Down	3.764946	5.063744	6.028357	4.294918	5.885892	4.490322
JHU12474	CNIH1	IgM	Down	5.301425	6.532096	4.401168	4.198505	12.21877	6.144472

JHU12596	NCLN	IgM	Down	2.618138	2.919667	3.86666	6.385617	2.025211	2.659534
JHU12662	GSN	IgM	Down	4.687613	13.8901	17.29918	10.85936	14.3181	6.261753
JHU12776	MRI1	IgM	Down	5.414693	9.941981	6.794624	8.420107	10.76211	4.814404
JHU12811	SQSTM1	IgM	Down	2.835152	4.247074	2.051438	3.822229	5.965046	1.83752
JHU12875	SSC5D_fra	IgM	Down	5.048725	12.13019	9.423434	3.389905	61.47154	7.793419
JHU12982	OPTN	IgM	Down	9.393919	7.423699	8.764739	6.174584	5.969711	6.270972
JHU13065	MBLAC1	IgM	Down	8.161953	3.469526	10.05962	4.492404	4.022915	9.473108
JHU13091	RNH1	IgM	Down	7.462585	5.99992	3.316401	8.750616	10.23453	4.22158
JHU13116	ASNA1	IgM	Down	20.67651	11.62904	24.37336	15.71287	17.6178	23.1893
JHU13287	SH3GL1	IgM	Down	5.620336	11.06919	11.60354	11.82797	6.235429	12.56161
JHU13549	PDE9A	IgM	Down	19.73916	15.53691	9.134649	13.73842	33.56743	19.78357
JHU13650	OPHN1	IgM	Down	5.544982	3.172393	3.71184	4.033165	6.664024	5.154437
JHU13702	CASP7	IgM	Down	3.311784	3.239643	1.150523	1.757775	5.603177	1.206359
JHU13719	DNAJC6	IgM	Down	6.16436	4.030406	8.270215	2.301981	5.519974	1.843895
JHU13793	MIEN1	IgM	Down	21.29421	17.06769	25.82004	25.92519	23.44833	18.41568
JHU13919	GAMT	IgM	Down	17.39541	10.22038	11.92104	9.869224	19.72742	8.849206
JHU14029	OPTN	IgM	Down	22.30374	15.2566	19.12653	5.688757	7.484058	8.3793
JHU14099	CRYM	IgM	Down	8.886251	7.873996	7.740478	7.583971	38.41726	8.362797
JHU14110	FSCN1	IgM	Down	2.92434	2.821371	1.85997	1.985579	4.127186	1.594546
JHU14154	PUF60	IgM	Down	7.516361	9.17532	11.21926	8.154882	14.41429	8.765077
JHU14157	SLC30A9	IgM	Down	4.199977	4.063783	2.839731	2.409087	5.73181	1.841162
JHU14520	AMTN	IgM	Down	2.146974	5.912131	10.65104	4.327431	1.940055	3.374803
JHU14578	PDZK1IP1	IgM	Down	7.712346	5.73854	3.421902	5.754772	6.051904	4.921743
JHU14730	CAST	IgM	Down	2.791662	3.530368	4.643238	2.645521	1.233704	2.794926
JHU14747	GAB1	IgM	Down	6.964564	6.185751	9.172871	2.741589	7.359592	9.485636
JHU14884	SNAP91	IgM	Down	4.405262	12.50296	9.169159	5.931492	3.42897	3.814075
JHU14891	TPD52	IgM	Down	8.216181	7.313464	4.750666	5.749226	9.651362	6.625612
JHU15089	DCAF8	IgM	Down	1.335261	1.42024	25.58025	1.880193	1.093153	7.566897
JHU15438	MUM1L1	IgM	Down	3.488482	4.894483	3.492342	4.441369	6.841245	3.694043
JHU15488	PLEKHS1	IgM	Down	3.615062	2.218466	2.824947	3.933275	4.383469	2.245428
JHU15549	PCDH17	IgM	Down	12.9435	10.7074	45.19574	10.85849	18.08715	7.519811
JHU15603	GBP5	IgM	Down	4.274509	6.695926	9.881048	4.439158	5.18212	3.459607
JHU15856	DCAF8	IgM	Down	6.279567	6.240169	43.36638	3.767383	5.960533	11.69352
JHU15973	CTBP1	IgM	Down	32.61671	33.98078	8.637387	16.62577	13.84152	11.17311
JHU15976	DGKG	IgM	Down	3.317028	6.079159	4.695522	9.599636	2.564066	2.61397
JHU15994	IGHG1	IgM	Down	6.967869	6.678096	6.975978	12.02615	4.620661	4.576886
JHU16309	MBP	IgM	Down	9.254452	4.463748	5.482314	3.986334	6.973478	10.79473
JHU16344	CNST	IgM	Down	6.470684	20.50627	10.88255	29.4272	10.57115	6.608448
JHU16347	DDX6	IgM	Down	11.27361	3.11612	6.548388	3.514092	11.10155	3.185669
JHU16371	PPP2R3C	IgM	Down	13.35807	38.38646	14.75826	24.91896	22.70257	17.37662
JHU16460	RCC1	IgM	Down	4.884114	9.974943	4.860651	3.004831	10.24671	15.36668
JHU16463	KJ901215	IgM	Down	23.02531	11.74903	17.53309	13.23628	17.76005	11.9119
JHU16463	KJ901215	IgM	Down	17.70539	11.78862	15.00731	10.24652	27.01523	8.075104
JHU16465	PPP6R2	IgM	Down	4.947496	6.524223	4.433482	13.52431	4.325055	1.947918
JHU16935	CCDC96	IgM	Down	2.695343	1.205953	4.592045	4.24437	1.005428	5.020448
JHU18292	MAGEA9	IgM	Down	9.872719	9.449464	10.16888	6.095677	8.667501	9.566488
JHU18315	SH3BP1	IgM	Down	25.74278	49.25491	115.1188	25.19506	48.2848	7.932218
JHU18445	COMP	IgM	Down	25.86572	21.99298	21.62199	44.99684	42.15085	10.91607
JHU19007	FSTL1	IgM	Down	3.962382	2.17055	6.289591	4.890002	1.536087	3.301467
JHU19682	PCGF2	IgM	Down	4.959516	4.041714	12.13978	5.587521	7.599617	4.274201
JHU19743	KLHL41	IgM	Down	2.595381	4.208774	2.285026	1.622825	0.574738	6.520567
JHU25614	NES	IgM	Down	6.133888	3.049681	4.465983	6.054783	2.319078	4.189076
JHU29124	VWA5B2	IgM	Down	16.91402	4.464079	13.26855	41.60974	30.86021	35.55161

and 2) the highest discriminant ability.
est < 0.05.
T-test < 0.05.

Table S1. All th

RC7	RC8	RC9	RC10	RC11	RC12	RC13	RC14	RC15	RC16
88.49736	33.93819	13.90037	31.54584	35.71152	53.88997	23.90891	29.38804	12.77783	22.88895
3.603554	8.276489	4.305689	2.35029	3.132166	6.172536	3.782088	2.132727	4.452703	2.219635
18.52164	43.16841	2.24487	2.415378	9.621935	1.720008	2.871081	6.529656	2.462011	6.152761
21.91384	3.299974	2.088494	28.50603	21.26705	36.28029	7.546342	7.868047	18.00952	4.332998
3.693092	1.776576	1.44383	1.684959	4.447595	15.46455	4.971563	5.162183	6.038464	1.659585
8.610973	2.557603	1.821833	10.99114	10.51855	15.36812	3.861639	5.176753	7.150006	2.566805
3.539511	2.478645	1.550993	3.022776	4.711898	15.02744	1.773301	7.201289	1.672519	2.088328
4.149074	0.843129	0.805431	5.50237	4.061106	10.18774	4.088874	4.292389	3.036264	1.211852
4.962191	3.693663	1.091053	4.697795	4.145633	12.95856	5.026267	7.920136	9.304157	2.110101
7.444199	2.093637	1.560517	2.724476	2.964063	2.926666	14.87299	2.207078	3.164428	4.009156
7.986453	6.618464	5.24344	5.078207	6.943136	20.66389	15.47582	13.07899	13.80202	6.125532
6.9826	1.461221	2.122395	1.729069	6.797784	13.4829	5.643065	7.06427	6.194491	2.845292
11.88973	4.87405	4.549847	3.564332	2.252793	3.034889	7.899524	9.029188	9.936553	8.42606
6.828125	0.602093	0.736974	10.677	5.17586	19.91346	6.366255	4.898527	6.166285	1.434182
16.04496	2.089393	2.153645	54.81767	38.83813	54.41627	14.40315	11.8767	17.1157	9.191748
73.16932	18.87538	36.9048	23.37945	49.42375	12.42244	31.64405	49.36882	33.97663	16.14448
49.80366	2.84988	1.650822	23.24587	39.41685	125.8518	50.8758	34.76803	45.66055	14.82489
35.60182	10.20073	1.786971	7.209204	37.21555	79.1812	22.4321	21.82911	47.83924	9.731047
33.77386	14.64085	4.768758	11.17058	10.52901	14.71356	6.011907	9.392144	4.837369	8.137954
5.434765	1.41184	1.752723	5.06823	4.653978	9.005285	2.177529	3.189725	8.018089	2.056799
28.64452	3.32406	14.72215	2.266053	14.93962	82.99629	4.674148	2.219886	2.559316	2.159912
8.717531	3.005719	1.832262	11.92573	3.239315	16.38571	5.248891	3.961917	4.983975	2.386842
1.841038	6.91514	11.36836	3.009466	3.013538	3.289406	3.020419	4.418409	5.825557	3.110269
4.007164	11.90784	25.01312	3.169539	19.30077	19.74723	1.597106	20.81583	15.26339	25.90307
5.780765	3.022462	1.6591	3.301492	6.208381	12.46386	3.568204	4.090513	9.96072	2.670752
2.796495	1.762003	5.910047	8.596361	16.47776	13.79002	8.857127	14.799	4.882484	14.01456
8.359994	2.539574	4.517161	1.77196	19.30197	15.29021	9.935487	11.90315	2.03307	1.821788
3.156992	2.05705	2.203111	15.23976	2.02093	4.968585	6.821062	4.230301	2.22191	11.59629
1.649659	1.921172	3.026998	21.36093	1.236392	28.38904	1.8166	1.460386	35.07795	1.554284
4.844711	5.224412	2.084352	3.856034	3.603063	10.50202	12.08784	8.179582	8.261143	3.19708
14.26329	1.112194	0.912289	1.878006	8.279577	26.29099	11.49502	10.57	15.10119	3.118291
4.487703	5.502416	3.650779	2.903986	10.24611	20.4876	15.8969	23.63208	5.10799	4.349339
31.22375	6.17896	3.042024	12.18074	16.25284	33.02747	44.75007	99.64302	11.8923	59.69987
5.872858	1.295965	1.520616	8.270439	9.491827	16.60564	5.438301	3.643559	4.647349	2.874699
11.52272	2.010029	2.308305	15.8652	6.815485	35.44521	9.127971	12.51717	10.04034	2.718079
6.157784	5.638936	2.362076	4.42813	3.302655	14.14745	16.5247	17.96376	36.93123	12.20449
23.40306	4.121169	3.967863	60.94104	55.36036	108.2267	25.95999	29.66051	45.66395	7.146156
7.092119	4.751642	1.928284	4.561371	3.228736	4.763877	2.335772	3.272533	2.084656	2.875437
7.111561	14.36608	27.29821	60.55599	8.048074	131.3099	15.16836	49.47641	6.670307	1.888377
4.213809	0.717252	0.669097	6.523167	3.919537	13.37678	4.734866	2.637514	2.368066	1.333317
3.583123	1.433292	1.253593	3.984544	3.380294	9.517854	3.866975	3.15468	6.187071	9.069554
10.42035	2.610189	2.207223	5.576025	9.940132	27.29508	11.21688	11.41838	28.60978	4.049287
18.22861	0.822545	1.460565	2.141706	12.98442	52.53546	14.7911	13.39831	30.68138	3.071794
2.955611	3.270492	4.851436	4.537833	2.359688	6.001601	12.27932	3.918132	6.328452	11.0819
7.316262	3.786518	16.4237	3.285772	3.724935	2.545676	10.53305	3.113598	9.962521	2.892659
4.519019	4.933273	1.467316	2.118216	1.825739	2.784475	1.380404	2.223409	1.096523	1.7605
2.966739	4.287634	2.49915	4.800021	1.81048	13.28522	11.76021	21.57667	4.759816	5.583745

36.56499	8.067124	4.510993	30.61411	40.19053	86.82779	17.89253	27.3003	41.33461	17.81183
5.301254	4.939182	1.851931	3.319641	6.799642	2.399025	4.704875	5.144695	2.470263	1.081839
5.252025	0.826865	0.905846	5.646523	3.937156	9.027554	3.150703	3.388437	4.753123	1.37448
3.074007	2.462829	2.771003	16.48163	2.184918	12.41568	16.85641	14.64865	3.485953	4.40473
11.48982	0.977651	1.067375	32.54586	31.05351	40.95679	7.387329	31.99974	6.556419	3.813357
3.00404	1.80405	1.772897	7.841771	2.601599	2.930826	2.561628	3.491321	2.462192	4.31694
17.36658	1.134954	1.029714	19.83148	14.82693	31.98969	10.23321	7.81575	18.00998	2.534853
5.984911	2.828286	2.668759	6.19619	18.70054	4.132397	4.575083	3.247478	1.97859	5.417706
5.199109	2.030735	3.844686	2.151924	7.558838	14.2329	8.210917	7.604704	11.62254	3.012859
2.166079	1.776298	2.361261	10.08223	1.458202	13.85947	1.93301	1.456924	13.13319	1.594818
3.667818	12.33464	4.032854	5.000416	4.827213	9.075146	3.39048	7.142518	4.543746	65.00646
34.40462	3.789062	2.140646	5.378014	3.166704	7.191939	2.518811	3.492318	3.177981	2.760382
3.467508	6.494738	4.015383	9.410596	2.594792	3.79117	4.552025	23.14491	9.501088	3.635601
55.0162	7.550779	4.657404	41.50294	26.99849	20.40264	10.06256	11.19351	6.631021	53.09463
6.975885	0.891023	1.811837	7.899668	5.132501	12.25319	5.487816	4.158218	9.057397	1.728942
72.75329	2.768896	8.259701	10.29351	35.80098	90.68147	37.84752	35.91242	43.49778	9.409012
5.223905	10.10648	19.05446	6.318009	8.059829	139.972	27.44608	35.5004	12.16886	8.068068
13.28747	6.488043	3.147846	15.6504	10.1022	17.12317	7.522749	17.06216	8.946592	21.07687
2.24802	9.176985	6.75723	13.67707	2.694349	86.26774	34.70661	3.747698	2.829923	5.877487
49.86864	1.830317	2.118799	63.13514	38.57877	93.18736	29.79521	31.42336	43.45563	9.273153
13.00592	2.285607	2.834165	4.201635	11.11454	21.59025	7.109719	9.223179	19.4882	4.320296
6.697482	1.019191	1.398874	1.414806	4.481578	11.46508	4.75627	5.083298	8.86774	2.030162
12.93779	6.14845	4.209487	3.746391	8.295574	99.00632	6.616625	4.860864	4.798477	8.567888
6.763283	3.505044	2.446492	5.743601	23.51121	5.68111	7.716249	2.990647	2.232399	4.954966
42.69921	61.01246	18.28371	38.35945	23.30741	85.44232	49.533	34.75918	62.96547	66.82393
121.4879	77.11743	14.08722	57.59613	83.26705	27.9161	56.32079	66.64214	27.23126	5.306357
4.698276	1.488372	1.555412	5.260921	4.993707	7.784585	3.622385	2.755522	6.438334	1.75696
3.326234	2.150728	5.910085	19.8032	2.651849	33.62818	2.607943	2.37322	26.84382	3.023841
12.31651	45.55738	22.16868	24.5073	15.59386	28.30553	16.16789	20.47443	24.41926	12.13437
4.486797	6.213356	1.177461	2.279213	1.556933	4.937677	4.697689	5.619429	2.092092	2.238708
49.76452	30.8046	1.814927	63.7334	53.58975	101.2714	57.05228	27.4794	38.20756	13.00137
6.977117	1.874381	1.334554	2.902358	6.793397	14.89705	3.862398	8.547772	10.22383	2.925831
15.1948	1.299408	1.855908	2.071641	7.844876	24.67091	8.31851	10.84525	13.7934	3.266553
26.08283	1.127994	0.923943	3.879723	23.85558	44.04345	20.09977	20.18011	33.67594	4.821003
2.900312	2.993565	1.916724	2.092063	20.49878	23.71692	5.571541	7.741196	2.671071	1.937384
1.228137	6.079542	10.86245	1.264581	6.627099	7.392251	0.841369	14.40333	8.037777	17.3595
20.03527	2.129667	3.682841	4.066284	15.18258	42.74196	19.00346	10.21223	21.48734	9.303658
7.344073	53.97337	4.419822	3.869131	12.36284	25.65734	5.224944	2.810061	3.106191	3.04913
5.007504	3.52883	9.542459	18.12563	3.392162	57.70013	15.99526	37.53946	3.598502	42.35293
6.166943	2.167987	1.295841	2.35521	6.952622	16.48286	5.591863	5.755106	12.3862	3.622719
10.27117	0.672706	0.722925	50.83183	30.43869	79.73736	19.03205	21.07605	17.97864	2.676468
9.349228	15.76641	3.297433	52.05739	6.077409	8.997985	15.00242	19.91771	4.410434	10.13111
21.64091	1.801056	3.050735	31.0687	14.77516	64.13524	15.12648	15.12595	17.76614	3.798329
13.47531	1.768376	3.327378	16.41909	9.691301	28.77085	15.35687	8.291298	17.05285	2.844794
16.21446	2.006257	1.809083	7.994003	7.150583	5.612895	8.345595	7.825017	1.855597	2.167473
15.304	0.893293	0.810335	2.276113	13.44488	23.27192	14.38338	11.60609	18.10579	3.302996
4.207012	5.056788	1.656779	1.824863	1.641524	4.082142	2.178874	1.357387	1.805302	2.198521
6.712321	6.463366	3.012104	7.244323	3.904843	24.75792	18.0898	23.74987	5.523442	8.528095
318.4116	1.373549	2.879692	1.465624	8.333523	16.25139	2.261684	4.663609	1.060718	1.960145
8.876884	0.917295	1.151508	1.364677	3.650917	9.856949	5.96277	5.667709	8.451403	2.591122
2.830817	3.182474	5.044533	9.170485	2.712581	32.56103	4.069605	7.903998	16.0576	2.058123
10.12037	5.276529	3.487104	9.625729	9.251761	18.89283	6.456302	7.539768	10.65487	3.491022
3.335263	1.053076	1.517092	13.04921	11.20296	15.45963	4.50628	2.486562	5.96242	1.655644
5.124553	31.36206	6.651671	3.061591	9.80903	12.8505	4.60753	10.31573	3.256171	9.143572

6.221599	8.469364	2.482948	2.899404	6.140265	3.931245	8.581247	4.650123	1.450775	4.827909
21.27845	17.15647	14.23632	17.57306	21.95324	14.04689	29.69218	17.37469	11.42162	15.27771
48.27376	42.77965	34.72562	39.66926	56.46193	25.62574	81.86271	47.53666	17.72565	41.87772
7.804086	9.04484	4.887704	5.572012	12.58631	4.997546	14.93487	11.93778	2.598509	9.495051
5.913047	5.746315	3.585132	5.311295	12.56458	4.164377	10.37019	9.975773	1.886594	5.947819
16.40169	11.01248	6.077944	8.640032	18.9119	28.4123	11.19037	12.18672	7.285588	12.87916
5.652323	5.108423	3.295434	3.632985	5.891803	3.439896	6.394866	4.764436	2.799216	6.584721
10.48284	3.066471	3.330739	3.098827	5.374688	3.702476	3.197712	7.466912	2.331158	12.33666
19.13721	20.65816	4.417344	4.080437	14.81321	4.307129	8.615758	16.43072	3.009769	9.167769
5.001858	5.165917	4.372295	4.349016	7.484405	3.267698	10.10488	4.176581	2.090514	7.334775
27.42591	14.85597	13.01217	21.25642	23.45579	13.76822	37.56536	28.73605	6.701708	24.61148
44.99906	7.057131	3.118464	6.245638	7.032943	30.70144	15.31157	34.4325	3.103685	24.53857
131.0309	89.36972	111.5151	117.0694	188.2272	62.97045	218.9564	104.6308	36.07073	119.9177
39.35769	43.8482	14.76082	15.91722	7.077199	12.56276	20.63134	18.31821	9.012907	12.25298
47.37603	33.42379	22.54975	52.18359	81.82987	27.1143	79.23157	54.0973	11.64939	45.17738
5.221132	30.57942	5.2546	1.079959	2.023553	1.418234	6.549697	11.81611	2.251864	7.078627
5.34934	6.016485	1.72393	2.902539	4.477182	2.30989	6.161136	4.105431	1.36885	3.479035
16.60154	23.15393	2.692051	22.52945	40.2676	9.528629	12.18913	28.03712	8.125072	10.28926
4.33193	6.149023	3.279523	6.411463	4.158817	4.737681	7.598012	8.781592	2.527818	6.304098
7.644047	4.85746	4.33686	5.964406	12.07881	4.016402	13.96833	6.042681	3.261449	8.580138
9.06714	7.627893	8.437836	13.43421	21.67179	6.399191	22.99291	18.44306	4.039086	14.34333
6.822631	4.489582	2.204353	1.679327	5.101876	3.138337	5.10208	4.888235	1.595838	7.01519
13.06982	6.593332	4.632193	10.68512	11.83957	5.206482	5.884156	4.469486	2.301559	38.17258
6.397182	6.226658	5.468099	3.08579	7.144553	3.916644	6.684543	6.471086	1.372996	5.71947
40.90812	17.38404	34.58872	38.75224	53.74208	21.45882	82.29306	47.2844	12.02141	51.51297
4.98133	1.690198	1.440892	1.702025	26.57164	3.983079	4.634955	7.198752	4.820986	1.598331
1.592378	1.508622	1.031337	3.252978	2.162468	3.324792	3.532037	2.242792	0.773729	3.205801
7.857723	2.019262	4.719403	3.563745	6.597274	7.289441	5.783433	4.047131	2.33715	7.478616
1.358964	1.393715	2.208804	1.534705	2.270003	11.02564	1.92767	1.858563	1.884605	1.977685
1.810331	1.04334	1.130989	1.032552	1.930991	1.659037	0.961906	1.429797	1.071887	1.212498
3.38605	2.294293	1.884742	2.824966	2.10145	5.19037	1.589877	2.880588	1.791484	2.304586
5.25755	4.519451	1.747641	2.072168	1.534878	4.89711	2.384503	8.429595	1.909819	4.996611
10.11142	5.761742	1.676953	3.080373	2.767622	4.802258	1.972929	4.322114	2.902851	6.106973
1.111897	1.731894	1.640243	1.104048	1.296301	1.137005	0.88341	0.941337	1.210601	0.990311
2.243347	2.782819	3.070819	1.729385	1.574858	1.929033	2.615425	8.886315	4.567905	4.061101
1.519852	1.15703	1.57528	1.316774	2.386109	1.652007	1.472762	1.33552	1.428343	1.845637
2.640386	3.60228	4.136417	1.51714	3.124245	1.754546	1.844692	2.09327	2.862783	2.465318
2.354537	0.915119	1.968867	1.163696	1.456888	1.08333	1.295155	1.031733	1.064104	1.70011
6.183913	17.84355	5.815807	13.37998	2.932778	11.14412	10.65971	8.4697	54.34405	9.534066
1.275111	1.916409	1.508467	1.507282	1.425557	3.011108	1.140457	0.970603	1.501952	1.361802
3.279303	2.348667	2.047337	3.013877	2.621573	7.90379	12.35896	2.971923	2.998102	4.600912
11.47312	3.728953	1.135214	1.419247	2.179856	1.159421	0.759551	1.468742	30.07651	2.332761
2.680582	2.369808	1.595167	2.167056	2.72403	5.069827	2.087128	3.821878	1.995357	13.15045
19.26664	2.881859	2.478729	2.136889	2.085723	4.692159	11.36165	5.149115	2.594298	2.311559
1.450932	1.620669	1.525464	12.18865	4.555261	2.843676	1.765948	3.772627	1.556439	7.729917
1.92903	1.053188	1.314634	1.503091	1.396005	2.091596	1.550083	1.447964	1.621967	1.206502
40.90403	13.18423	43.92484	29.58599	16.0735	14.6947	40.85	9.224512	5.991819	13.81881
15.10015	11.97828	3.076816	33.76016	7.856043	6.639505	24.8682	10.75719	2.385123	14.10344
10.43713	12.29737	2.138773	3.962348	6.343209	2.753251	2.558423	19.51765	3.12879	6.176341
3.683941	6.713283	3.662331	1.3436	2.494032	3.695486	1.748145	2.480643	0.91584	2.783964
7.861325	5.939893	3.868219	5.426317	4.97197	7.635628	4.763971	4.401596	1.679362	4.88921
3.2813	3.267642	1.923883	1.895453	4.084659	3.939676	2.67857	3.933699	0.993465	3.538591
12.76806	11.07839	2.65377	9.366273	23.96381	9.809764	72.21046	7.625581	4.022673	16.15836
8.447323	6.067336	1.961621	4.226064	14.16784	9.482973	9.446252	8.378945	2.050364	7.528801

5.166528	3.079437	4.903243	3.045093	5.92811	4.072748	3.539022	4.915074	0.869963	4.607198
13.16434	5.046054	6.689341	6.996117	5.681885	6.534447	11.21249	4.413191	2.140047	5.675573
14.64679	18.10352	10.80983	12.41507	6.282884	13.8823	22.09224	10.0392	3.468947	8.31164
5.935811	4.916122	3.501908	4.346694	4.632747	6.441279	5.791647	5.837834	2.883625	4.485421
2.1835	3.035961	2.750858	2.461237	3.889299	3.594269	6.495667	2.528929	1.746271	3.792426
10.11143	6.916511	4.338392	7.657422	5.700115	9.255626	7.345547	7.892789	3.293115	4.702289
3.066673	2.495095	2.906099	1.701079	3.439961	2.928731	2.42397	3.571122	1.094783	2.780138
4.850547	4.922745	2.802759	3.215627	6.791064	6.50778	3.504081	4.890086	1.144157	5.814501
7.361141	3.777887	2.955567	3.76136	8.633655	8.413249	5.271523	6.677641	1.112995	5.764596
4.697983	7.179177	4.78064	2.832367	3.644635	6.443617	8.716957	4.044472	1.646818	4.285557
8.67368	7.662441	3.852259	11.03765	5.96991	11.87467	13.55233	9.77179	2.535084	8.890913
10.95862	8.543746	6.560268	7.521905	5.830915	4.15453	3.393964	4.041737	1.300258	10.86601
2.598609	3.833958	5.100186	1.512137	2.031637	2.719186	1.83396	2.149717	1.107683	3.698247
5.257367	7.466852	1.964207	3.169128	5.177296	3.358169	8.693446	13.85053	3.792673	7.761175
7.958371	5.865785	9.984042	4.663209	6.535342	6.21597	4.538258	8.213568	10.07919	5.4999
6.072388	4.669824	4.73839	5.209734	4.620303	5.964248	3.756288	4.141762	1.779228	4.039698
4.87395	7.280007	3.180371	2.630905	2.163747	3.150741	5.456014	3.702677	1.69502	13.17327
11.76724	30.135	3.993182	10.75629	11.26838	14.79029	5.976426	22.7697	4.115215	10.80908
11.84921	7.061967	7.697878	4.733015	12.52818	8.494844	8.067301	14.25062	6.291512	9.683265
11.23013	4.145911	1.995397	4.558557	2.60405	5.445912	5.918716	2.759606	4.83945	3.435578
18.02103	9.053499	3.566064	16.43119	14.88853	11.30329	10.55821	10.77041	5.091178	18.26648
10.63053	6.22455	1.995603	2.365759	78.96114	2.962512	4.272544	5.060267	1.491325	4.830819
16.87087	7.131313	2.657556	4.375792	14.91289	4.291826	8.505487	12.73397	5.032817	18.04577
23.62682	16.80622	5.394137	18.36067	21.70247	15.92138	40.73138	17.71407	2.769905	14.90519
5.008344	4.212452	3.741287	2.515385	2.066058	5.397333	4.836406	2.293013	1.117508	7.044872
37.34544	19.13685	3.603937	9.822717	13.99398	21.80629	22.78327	46.84532	4.407646	89.67151
24.74868	24.30229	7.112985	9.977584	14.19391	18.77341	21.57129	13.88186	4.200051	17.74829
2.455999	5.725001	1.788354	1.912218	1.628706	3.434408	2.811794	2.683937	1.445517	2.085906
40.13477	8.125125	7.644022	4.304174	4.079368	6.071571	2.523918	3.517435	2.176545	7.207678
11.61823	10.91896	4.307015	8.706425	14.42103	15.45431	11.37293	10.07179	2.739544	7.852125
15.90387	4.441472	2.717861	7.030974	6.667532	7.620879	3.678697	37.40859	1.196309	4.4568
8.446892	8.528035	5.17088	10.42379	4.267155	5.22121	8.443969	24.42554	7.398866	11.93922
20.08008	8.373525	5.519161	13.18348	11.89196	16.35581	7.592172	8.146852	4.619694	9.189979
14.1983	17.05217	5.025101	16.14078	9.362798	21.71012	10.59599	9.684526	4.220413	8.579317
3.893971	4.402403	11.75208	2.280621	4.825138	5.053793	7.539679	3.495391	2.01261	6.099085
10.30288	2.004573	0.695912	1.988523	3.16498	1.604468	3.402263	6.596961	2.032222	1.318209
8.069022	10.25663	1.268574	2.289152	4.747426	6.55989	2.940656	4.102432	1.824698	15.81135
1.653899	2.680371	0.948504	2.16374	6.755805	2.286165	3.989787	1.846924	1.078232	3.219268
3.420048	3.997594	1.743835	3.830081	4.564951	3.01775	5.055667	2.503592	0.904931	4.049523
2.979534	2.887604	2.26927	1.646492	2.015283	3.566532	3.680444	1.611261	2.457449	3.63647
4.311776	4.14545	2.763936	4.106644	2.47536	11.50782	10.68797	3.652108	0.73682	3.265514
8.630469	7.863369	3.760413	9.939204	5.878708	6.861755	18.15598	8.465762	4.393073	5.658422
16.228	31.1839	4.699879	33.71866	9.05802	10.41402	11.54275	59.13829	3.285719	15.62168
14.0497	17.64743	5.458046	5.430108	13.14452	20.30999	22.34597	11.52854	4.464732	16.30501
30.51594	27.14314	4.716114	14.43304	14.22407	12.40549	31.48414	23.85393	3.264852	35.27533
4.366713	3.617964	1.366796	2.390876	6.246743	3.887296	2.709569	3.316306	0.969144	3.826634
5.437475	2.980632	6.414013	5.286149	1.923618	4.574259	1.580824	3.476148	0.525252	2.453295
18.97049	10.64376	5.748158	14.72448	9.674427	11.86367	18.86895	6.09347	5.822733	15.35331
9.460302	10.6973	5.563816	6.398889	9.743652	8.259089	4.829517	7.315118	6.35125	21.69153
9.08372	10.37935	1.407708	1.886161	4.369135	2.95492	11.16723	6.176527	0.931281	8.850729
11.2411	7.16581	3.85475	6.614156	6.730876	8.870274	4.251527	4.744333	2.253348	6.842057
3.17492	4.298905	2.740536	8.876896	3.119113	5.342459	6.378089	3.32133	2.425347	5.256757
20.13695	6.056425	3.347336	5.769855	3.892821	2.267364	5.901553	4.945092	1.895513	3.053695
25.11598	17.51909	4.746336	11.69315	10.7428	5.942526	9.280398	11.58933	2.542305	19.24381

12.47041	15.84266	3.500343	12.45102	11.81833	4.546388	6.646924	12.87282	1.83057	12.77755
3.884496	3.162518	1.954238	2.465419	2.472091	2.429537	2.109905	2.205696	1.184866	4.445358
6.714722	3.968083	7.199267	3.265313	5.0377	5.116105	4.568312	6.640984	1.670469	9.55535
4.989706	4.278617	3.308072	3.965144	4.472518	5.577864	4.421034	2.656372	2.976133	6.852845
12.11487	12.27975	2.661162	5.582977	6.340005	8.520427	13.30608	8.332477	2.843256	6.201403
18.81239	12.52265	10.18529	13.23724	10.22636	17.15307	10.70284	12.21301	4.032319	31.61817
73.00898	15.35947	6.713281	40.53455	9.689566	7.568113	12.26327	11.9999	4.970116	15.71714
2.60832	1.391154	0.588081	2.604162	2.177797	2.120188	1.579549	2.229855	1.30134	2.504338
6.652635	2.678213	1.251796	5.459039	5.223022	2.528099	4.509932	9.239564	3.469805	8.388633
24.42313	16.40772	6.814422	16.16094	10.93584	27.23449	15.72667	19.47303	7.607025	19.32009
7.236735	4.873073	3.273254	3.327469	6.013371	5.125536	4.112481	3.401512	2.043844	6.141341
3.039273	3.234953	1.711091	2.790041	3.385094	3.508041	1.881101	2.71835	1.011108	4.353628
13.892	11.95345	5.05906	26.85628	20.61752	19.20533	22.58823	5.626163	7.521027	11.59799
37.0061	27.23072	4.421199	18.55459	10.9801	18.45608	14.05123	62.23597	5.433666	14.56885
15.65966	9.053254	3.193392	11.84515	5.346116	9.035666	11.10124	7.709121	1.787363	8.609358
14.50282	14.59002	4.150467	4.776831	3.263615	5.868075	37.92562	16.0286	1.926711	30.30303
4.591708	4.773045	2.308874	5.111407	5.567325	6.970823	10.03494	9.560603	1.877942	6.502795
30.91458	10.38314	12.40536	18.57661	9.212326	15.89334	9.357533	21.11859	3.414139	16.23613
2.000913	3.180266	3.571741	1.904705	2.754522	2.931984	4.443047	2.418603	1.674683	2.491743
2.951525	1.619101	3.327808	2.746254	2.976415	2.973966	2.457878	2.547153	1.157887	4.531098
1.766586	1.255959	2.329358	1.797287	1.518014	1.414354	2.343185	1.676289	0.914472	2.547078
20.77288	18.53311	19.814	13.36796	15.35777	19.91929	24.63809	12.82116	2.966347	12.63053
2.955842	2.699284	1.346013	1.418356	2.371574	2.796982	2.222837	2.492742	0.867713	6.38975
6.041292	3.059839	2.534155	2.88066	3.133204	4.581833	3.733011	7.379795	2.328339	8.079027
11.27563	5.810629	3.009081	6.790358	5.581166	16.77354	8.723121	64.51925	2.829773	8.540855
7.575485	2.02847	1.6608	1.603487	2.469252	5.970084	5.815937	2.607586	1.845844	2.926616
5.7771	0.268552	2.466457	1.925814	7.516841	2.944501	28.5685	2.03756	1.596365	2.502697
9.529473	5.098033	2.612313	3.174219	4.456945	11.59506	4.632882	14.00475	2.467372	8.989851
10.24534	10.4353	12.51012	9.069668	3.295109	3.895428	6.951818	10.00514	1.784189	5.171466
9.854355	11.32196	10.99568	8.686961	7.408599	3.104261	5.5894	10.25687	1.650081	4.931037
9.965759	5.593694	1.760839	11.2995	4.191037	5.185818	3.628955	5.294354	1.397297	7.694476
9.906996	6.770588	17.18206	4.695049	7.219247	7.625866	10.35089	8.179314	4.314797	8.478366
6.613055	14.45744	1.261624	4.336059	3.389846	2.803051	3.499048	2.87814	1.136009	8.202518
4.249008	7.923116	1.629748	2.600305	2.070329	2.006127	2.177146	2.147043	0.993564	4.065441
15.09241	8.70384	6.219581	6.896896	7.195416	4.248235	12.93155	7.461404	12.25976	13.09719
10.06191	8.548477	4.127465	3.873631	9.121745	3.178182	8.65992	5.219124	6.655907	11.56443
14.53379	5.462548	8.881481	5.712168	5.559294	4.880062	9.339124	10.59104	1.598212	10.11829
22.77766	26.95511	4.448335	14.26691	18.92734	14.3665	22.93782	70.79438	4.439912	16.80833
4.458258	1.693345	1.035315	4.071428	6.565173	2.77477	2.439338	3.08512	1.016392	1.498892
8.764126	3.411845	2.86854	4.995222	4.530889	5.137723	6.456589	13.53776	1.548837	4.517633
10.07286	18.19182	2.1141	6.71199	7.070019	5.710386	14.40035	28.54168	1.487528	7.491142
10.95078	6.229966	5.990412	2.296299	2.454117	2.704105	4.37384	3.265885	1.430453	3.379304
8.744131	5.561106	4.532535	3.902708	8.061417	6.348179	5.418224	5.863472	3.348319	8.656037
23.36317	7.503458	8.555781	15.82306	4.569723	7.096911	5.598989	16.45661	3.20141	12.25038
10.13958	6.761587	4.333645	8.245191	13.02211	12.31718	26.51683	9.964102	2.731064	9.835428
5.337404	5.008763	8.187016	32.7075	7.072276	5.401393	5.56609	9.22325	2.877231	14.81548
2.589773	3.769965	2.191913	1.956249	2.875653	14.43195	1.682682	2.983473	0.801213	3.204937
5.410864	7.943013	3.894855	6.451697	5.23086	6.09093	5.520404	9.127004	2.788847	7.390741
4.397811	3.042744	5.497342	2.962489	2.870615	3.006805	4.690881	7.049418	1.120213	7.06472
2.840325	2.023406	2.996267	2.584667	2.698208	3.310839	2.771626	1.648446	1.216218	2.976236
108.5808	7.698846	12.5277	5.277108	3.140547	4.788353	9.707579	8.103866	1.731825	4.479534
17.89517	10.79122	2.357494	15.16815	10.19918	11.90479	20.62773	11.28429	9.764181	15.76526
3.804435	4.100716	6.18736	3.527681	5.614206	3.69342	2.35951	10.12053	2.454733	3.446058
8.157529	5.909941	7.395583	7.655069	8.39524	8.887271	4.311389	7.214113	2.647986	7.172999

3.031121	2.541149	4.37122	2.673598	2.329715	3.479417	2.014573	2.813674	1.815657	3.982056
13.34351	12.58146	11.72839	5.450193	5.428859	5.660012	8.938732	14.11737	4.920894	9.855267
19.32668	6.148623	4.169483	3.59706	3.903712	6.374852	6.526225	3.283931	1.943332	6.566669
3.976966	2.438949	2.30442	2.723499	2.633221	3.527608	2.669678	2.750271	0.711031	2.572312
7.295067	6.614197	2.869181	15.60917	4.84414	5.60665	3.397468	4.752291	2.277921	6.230789
11.81947	3.627192	2.362823	3.393297	8.288357	3.876603	9.131527	4.45386	2.945832	6.477957
6.917819	6.799537	11.84635	3.235609	8.446877	5.589401	7.610919	5.156312	1.06725	6.791177
11.06111	3.492681	2.589131	4.332042	2.414172	5.347662	8.631511	2.673257	11.81888	4.653597
17.46068	8.450125	2.683996	19.26748	10.85874	27.53821	27.70834	13.08496	4.281877	10.13544
9.599573	8.020924	5.624315	3.795074	12.84078	8.424788	4.301514	8.080668	4.664873	6.831554
37.41179	17.83888	6.444834	23.28093	25.49937	28.61521	15.41193	21.50045	5.765714	15.62671
5.937626	3.160381	2.158519	6.293538	2.826002	5.400275	4.780152	4.220985	0.967532	4.103154
2.897252	1.635738	1.883933	4.748458	2.346986	2.759849	0.942923	1.749401	0.985219	2.953358
4.026843	3.960338	5.107219	2.328977	6.061499	2.788625	5.4685	4.894366	1.384839	3.075947
12.93431	11.28567	7.533463	9.617048	11.45719	8.347689	11.38419	9.925785	2.501935	9.143307
19.12382	9.76805	4.869067	23.46596	10.88242	9.743918	9.310788	12.76333	3.025184	12.32873
23.88909	5.786397	6.076399	6.536465	16.38087	6.653966	12.738	8.894417	4.066008	12.13128
8.684448	7.281033	1.829515	4.720365	5.194759	5.939468	3.538681	6.685014	1.109865	5.527766
3.928441	3.40521	0.702063	4.570794	1.905645	3.541206	3.194321	2.0932	1.3215	3.923675
6.182598	9.651228	1.697728	7.15309	5.936606	10.76298	6.023735	15.63783	2.273172	10.04674
5.344096	3.521196	0.886003	6.913209	2.983891	6.514624	3.258673	2.737672	1.462199	6.514949
3.550542	3.989273	2.674497	1.974586	2.54333	4.501827	4.546661	9.011148	1.166528	4.503736
5.655803	3.88249	6.798783	3.799645	4.791469	3.679253	3.66975	2.991378	2.234706	4.049704
6.436435	8.211845	2.672227	5.868173	1.405388	1.511643	4.986835	7.702639	0.481382	3.702245
8.012896	7.093444	1.973243	3.411178	6.157927	3.404896	7.688433	6.024738	5.73024	11.92857
4.361892	3.528593	2.593218	3.816836	4.725851	2.240787	4.434576	9.162226	1.434985	11.19209
3.113008	12.11435	1.972168	2.265829	1.791889	3.319094	9.33496	2.895268	0.904527	18.19333
1.713049	1.5548	1.446136	1.278005	1.424013	1.844231	1.500474	1.508726	1.364762	3.057723
7.575119	4.869019	1.706381	2.588109	4.782794	5.373078	2.616995	4.590645	1.102576	5.863224
3.133996	3.21968	1.850211	2.502475	2.611014	3.528477	3.081897	3.526974	1.45115	6.340736
18.87834	11.5498	5.787062	19.75463	8.095094	8.452355	7.014169	7.10883	2.591739	17.01843
5.764976	5.999989	7.973682	3.009085	3.201344	6.973272	3.247834	6.051505	3.079161	6.1807
6.653471	6.476148	2.10285	3.685513	6.441681	5.210296	6.386304	6.75871	1.767198	17.25214
22.05773	9.053781	4.884397	8.492557	31.3181	12.27367	28.43448	20.35363	3.825343	11.52149
3.523	2.925301	4.930166	2.978118	3.151257	3.005675	3.04945	2.452479	1.909906	3.380822
5.516303	3.381777	5.769691	4.045218	6.428723	4.377665	3.717851	5.075505	2.183532	6.591718
10.61374	13.26238	2.556364	4.276377	3.174826	2.822	3.142726	5.009927	3.375909	3.95438
14.18141	14.99587	6.042891	8.979407	6.419588	9.010915	10.24895	19.18067	4.989277	9.559389
4.934874	4.061781	2.081478	2.695822	3.762221	3.09508	3.956916	3.712771	1.248335	11.24285
12.70186	14.53506	6.803615	19.11294	14.19469	10.69022	7.667985	7.76016	7.148477	14.00458
3.728072	25.94371	2.550799	6.079395	9.918307	6.292409	11.68634	3.939201	2.039476	5.106761
17.50289	21.94765	11.6148	6.604057	10.51621	11.81588	20.73184	10.79131	3.34183	10.84228
10.43331	13.08704	17.88827	6.124448	9.359036	10.36116	16.81094	7.318753	2.36906	8.81169
4.720834	3.355808	3.368203	2.754304	6.708176	2.249844	3.961489	7.95965	1.379207	3.03511
1.899216	1.970101	5.126573	1.338723	7.85186	0.814443	1.73155	4.94288	1.005021	2.650445
7.398272	3.82998	17.95548	3.031499	4.480983	5.188552	7.121112	4.807303	2.045961	5.668343
15.10459	15.12546	3.108865	9.259596	23.75453	15.99888	15.12419	51.45557	6.720032	22.20864
31.95009	8.724398	7.947841	20.70902	27.38379	28.18952	21.08423	30.33277	12.20004	35.00112
4.414783	3.897695	7.204968	3.004307	5.364274	3.394941	3.877259	3.40378	2.378027	3.914716
4.1487	2.694536	3.792738	2.008428	6.466365	6.629231	3.334756	5.586051	1.436721	7.93292
1.752145	1.784293	1.445276	1.914397	1.2325	4.413544	1.204129	1.406495	0.672262	2.810587
4.828175	8.194098	1.887489	3.527101	4.337228	3.37003	6.178076	2.590774	2.377866	5.066881
16.42967	17.98013	10.93973	31.65451	16.87907	21.45032	15.11925	14.80441	7.082276	12.20431

ıe significantly different target proteins

HC1	HC2	HC3	HC4	HC5	HC6	HC7	HC8	HC9	HC10
11.54161	12.87318	14.52667	11.97293	12.17903	16.94395	12.21262	13.05815	18.46999	26.2835
1.77258	1.378883	2.101715	2.014488	1.432096	1.089153	2.695757	3.003997	0.99115	0.990797
12.32789	2.569929	2.371697	2.312513	1.32698	1.40302	1.67338	1.689707	2.20857	1.596118
1.577595	4.231935	1.472267	1.937519	1.254139	6.714274	4.468585	6.260458	1.177429	1.701456
2.583838	2.079942	1.244997	1.387153	1.339159	1.614379	2.548685	1.354247	1.499989	1.284272
1.932919	2.264888	2.223073	3.904687	1.825209	2.320207	1.9736	2.165996	2.677009	2.821647
1.699957	1.654134	1.534262	1.504332	4.46612	3.086569	2.228094	1.418768	2.228514	2.418494
0.602846	0.942532	0.642823	0.853585	0.679153	1.371025	1.468982	1.132452	0.700825	0.763831
0.414446	1.599496	0.750976	2.179018	0.828544	2.145335	2.61212	0.874523	2.195591	0.730561
5.995469	1.808806	2.176616	2.386715	1.41988	2.303359	2.125901	2.9087	1.50276	2.922896
9.653455	3.757379	5.045342	4.400522	4.018149	4.867315	8.895014	4.186989	3.8967	53.89453
0.566473	1.239255	1.020804	1.34184	1.439718	3.568119	3.051463	1.332682	1.084797	1.099061
4.155106	1.64296	7.875477	4.195029	2.091305	2.189878	15.05045	2.568873	3.428492	3.787754
0.25594	0.843104	0.565907	0.588172	0.682217	2.364411	1.692786	1.197542	0.759061	0.513514
1.451167	5.598591	1.71506	5.404735	1.565735	15.7059	7.721168	7.289743	1.300441	2.13368
30.96393	7.62301	10.12767	8.047303	14.62359	4.612705	14.76453	5.674375	18.80173	7.460141
3.184432	4.506574	1.252776	15.68281	4.137331	15.83501	22.29785	1.494892	1.511592	3.340782
0.767572	1.892782	1.019132	2.884936	2.464423	12.75037	11.43937	1.228266	1.166161	3.223549
6.319354	6.188741	6.901631	5.785932	6.175999	6.609905	4.453943	5.451568	8.480339	12.32673
4.926951	1.775099	2.225744	2.07992	1.341345	2.137364	3.012056	1.773597	1.256531	1.211894
9.597195	2.82557	3.338716	48.41233	1.419911	5.815822	1.642447	2.425424	1.581517	2.239256
1.83408	3.158265	2.050103	1.566578	1.84308	3.274893	3.163581	2.577886	2.878838	4.545318
2.968463	1.984356	2.678225	1.728152	2.315251	2.388276	5.283771	3.756469	1.937465	1.679905
19.65422	9.586032	3.421994	3.336575	1.889217	3.392281	1.77501	3.437697	1.939603	3.699003
0.800117	1.34111	2.292108	2.457984	2.547856	3.451917	3.384327	1.602524	1.132157	1.69992
1.390991	2.120103	1.392454	3.892536	1.068561	4.539162	2.863012	6.019861	2.909031	3.149819
2.466587	4.550601	3.006406	1.360131	1.650036	2.281546	2.654014	2.402129	1.772205	2.664806
5.177914	2.223615	2.550117	2.276232	1.624824	2.700326	2.500542	4.382896	3.540201	3.230148
10.06742	2.430955	2.986379	2.630936	1.081756	1.092446	1.009214	1.165236	1.487453	1.429344
2.194336	1.738219	1.722465	2.516439	1.620573	2.7154	3.686816	4.497742	2.978748	4.082455
0.636628	0.990276	0.764834	1.269196	1.106856	4.11962	6.603467	0.716197	0.867166	1.136457
4.004477	3.108739	3.504494	3.020856	2.119238	3.793521	4.465958	3.653756	3.572212	4.236914
3.252853	7.30515	2.819291	2.824684	0.996251	5.524657	14.13231	8.622652	3.111676	12.05267
1.159118	1.554878	0.755673	0.980315	0.673513	3.531809	2.514902	4.068725	1.100375	0.857891
1.345485	1.425277	1.284504	1.426054	1.581892	11.59543	5.010405	2.559774	1.437331	2.333403
2.867058	2.042807	4.572876	2.857676	1.722093	3.494221	6.217412	2.734823	4.667235	6.611892
48.41504	14.01948	8.345695	7.558657	4.053661	4.037929	11.84735	21.52768	4.29421	3.689526
2.547476	3.49019	2.294094	2.435979	2.730015	2.510548	1.668694	1.962279	4.207789	3.321237
1.203729	5.929918	30.66781	9.424574	1.559129	1.91599	3.998832	11.16172	7.706877	4.686625
0.515413	1.742628	0.673966	0.968889	0.579396	1.591467	2.476471	1.133701	1.868788	1.03104
4.021965	1.690062	2.019501	1.621576	1.130734	1.890512	3.050965	1.510522	1.351479	1.667916
1.479179	5.702548	1.792049	4.419025	5.139401	5.615065	7.221607	1.831217	2.455273	2.211033
0.55439	1.380828	0.81055	1.592759	1.401958	6.229888	9.019146	1.36382	4.369124	2.19908
1.586348	2.533985	2.641891	3.075929	1.883359	2.741281	3.356121	2.902311	1.726394	1.496643
2.116739	2.440363	2.209615	2.63636	1.604453	2.92736	4.602651	3.537575	2.476156	3.195924
1.574137	1.781293	1.543988	1.457502	1.674767	1.970671	1.36711	1.971604	1.924005	2.976516
5.28683	2.378722	2.866273	3.426825	1.88523	4.256291	4.39562	1.785516	2.615847	5.288023

1.706151	23.81789	7.921392	12.6396	5.870192	49.84612	30.12722	8.183872	6.90991	6.130564
2.83853	1.860282	1.644675	1.602156	5.280918	1.681732	3.576533	1.940178	4.688627	3.445885
4.490897	1.47706	1.43148	1.68173	0.769968	2.522242	1.987132	1.758444	1.875903	1.100377
2.234784	1.725905	1.969756	1.928655	1.535061	3.669967	5.721365	2.369911	1.914358	3.60476
0.965692	1.449633	1.145233	2.989005	0.833508	1.7923	3.771963	24.42465	3.011278	1.216245
3.615704	1.879767	2.26525	1.586602	1.180287	1.865572	2.255098	1.731766	11.30936	2.095496
7.413702	2.447224	2.264468	2.644461	1.049605	5.185311	6.147921	2.789147	1.206985	1.582811
3.514732	2.114685	2.924161	2.910229	2.408197	4.353291	2.089982	2.569807	2.65619	4.228874
5.86026	3.875398	2.6145	2.649901	1.39153	3.376735	3.347383	3.366545	1.790831	1.787642
12.60665	2.754932	3.871457	3.343519	1.030916	0.986104	1.720654	1.208543	1.791257	1.215057
6.729541	3.275053	5.021795	3.20018	4.199675	6.247991	3.528052	2.99558	1.661394	3.608305
1.823599	3.089137	7.512858	2.072821	2.218568	4.359139	2.356451	1.693249	3.678184	5.106294
3.17175	2.259241	14.6145	2.155292	7.070428	2.956716	3.905491	2.993692	3.841716	1.932166
18.86849	6.068124	6.392355	18.26985	7.942519	5.75791	9.865487	11.18782	10.73609	12.04736
1.183913	1.171913	1.076406	1.100473	0.972798	2.416503	2.428431	1.800144	0.970279	0.958224
1.101742	6.457963	2.126	5.095508	2.07756	19.10498	19.62764	6.395926	3.77779	8.284739
8.997346	7.081379	5.024366	4.280662	2.418303	19.06897	8.136936	5.391275	17.50871	15.3732
9.080958	9.63312	5.152477	5.208393	3.872551	11.19052	19.06123	3.967101	5.609934	7.65022
19.96605	5.255686	6.254441	5.041886	2.287578	5.087839	8.153121	2.352658	2.949386	7.75711
1.609121	3.797495	1.57695	3.075938	1.681371	14.16863	13.98435	11.4247	4.369908	2.500828
2.61926	2.060231	2.588734	2.040369	2.092531	5.755034	5.547105	3.731728	1.902317	3.060239
0.551853	1.006497	0.927195	1.108279	1.164966	3.440303	3.022994	1.07912	0.848512	1.169981
1.685161	2.728283	2.458695	3.847825	3.668825	4.21471	7.171932	4.518203	5.858139	5.103284
4.499165	3.369029	3.607151	2.343922	2.904385	4.862099	2.793613	2.398184	3.926763	3.682615
6.65038	8.95995	14.1068	22.91241	15.83441	38.43932	47.86143	33.13963	19.01602	13.10521
30.51823	24.74128	14.96957	13.32147	74.96889	27.00659	49.17339	26.3814	60.42476	53.00587
1.558065	1.37695	1.614504	1.604876	1.091402	2.042329	3.632585	2.188592	1.642923	1.872188
14.72617	3.056503	4.548841	3.425892	2.226482	1.869496	2.92772	3.082638	2.105654	2.995686
6.12574	2.832067	8.51426	17.66751	13.06438	6.404486	14.84871	14.12805	5.62627	3.653699
2.27249	1.751191	1.975502	1.796351	1.592292	2.493354	1.287088	1.362197	1.713066	2.460516
1.30289	6.114486	1.06554	2.508632	61.49578	23.87679	18.4665	16.1685	1.335632	4.71461
17.00825	3.777797	4.262892	4.19323	1.955154	3.221664	2.808984	1.456182	1.835022	3.388201
0.978604	1.890783	1.615336	1.350847	1.601185	3.92005	4.939784	2.901693	1.073601	2.950817
0.476903	1.394726	0.695706	2.914643	1.519993	11.15919	15.3699	0.826765	0.840725	1.649741
5.291864	3.496735	2.538296	3.593347	1.665104	2.384966	3.543619	2.340695	1.826629	4.315658
10.47486	5.003334	3.328989	3.322124	1.134702	2.548805	1.048933	1.615743	1.406146	1.577147
8.137301	9.001099	3.638428	5.124606	2.136869	10.90559	24.23381	14.72865	3.171721	4.061805
4.935196	2.986661	2.885675	4.049043	1.675745	2.148529	3.121993	4.362622	7.035065	25.49235
6.384732	5.182577	3.540846	5.929805	2.021723	9.585733	11.00426	7.195812	14.42407	8.505007
3.020089	1.956523	2.519473	1.649693	1.806763	5.405246	4.940051	2.705192	1.620059	2.381833
0.283605	1.380436	0.634084	1.251812	1.143421	1.054821	11.57022	7.445115	0.862106	0.960361
1.915398	5.232285	3.354992	2.299027	1.782764	3.962717	7.161481	2.045743	1.963819	4.169932
1.428407	2.015394	1.401697	1.503976	1.294641	20.01512	8.65477	3.231516	1.482348	3.845709
16.38359	5.569616	4.336827	4.591595	1.903855	4.559734	5.820748	4.177933	2.547085	2.331632
4.903938	1.862295	2.21091	2.279783	5.108335	1.541215	3.662015	2.599979	1.794753	3.014473
0.517651	1.057441	0.70636	1.646665	1.216047	5.629335	7.890217	0.761612	0.884191	1.137344
3.001803	1.357853	1.728839	4.966825	1.375292	1.557885	1.992903	1.997218	1.354413	1.277794
7.416802	3.756784	4.148299	5.142878	2.567826	6.934574	7.081721	2.521429	4.006333	9.510595
2.867308	1.700733	1.313358	3.914968	1.504037	1.466538	1.660187	1.841569	1.513134	3.89832
1.006307	1.147029	0.995682	0.928901	0.892938	2.449147	2.706011	1.185183	0.986521	1.221641
7.142772	5.208162	3.497065	3.655864	1.300981	3.584483	5.646443	2.058923	2.127396	4.485903
8.691321	4.01997	6.124381	4.027174	1.925775	3.098526	6.988532	3.765372	5.744467	3.412473
0.960182	1.308983	1.169248	0.959862	1.348228	3.837916	4.31104	3.994383	1.490674	1.234309
5.770255	3.095822	2.455948	3.585651	2.202428	4.276393	17.53865	3.323995	7.473403	3.6509

2.104011	1.714984	2.788465	1.617294	1.822171	4.836864	6.497001	3.190642	3.768389	3.318883
12.99741	9.679603	13.23082	5.642082	10.35841	14.6083	15.49542	7.799576	14.86979	6.645666
25.03807	13.03488	38.26923	10.75113	21.50433	42.56052	36.33709	19.41328	40.85751	16.23359
2.820965	3.354792	6.77116	2.8238	5.039475	7.288011	7.623972	4.508554	8.558667	4.511384
3.28812	2.874372	5.257769	4.638596	2.66485	5.543799	7.131598	3.927327	5.597696	3.350347
7.451541	7.173261	9.603557	6.754521	10.65284	9.325898	10.77869	7.351809	15.89787	8.895648
3.47521	3.059365	3.988727	3.23807	2.95708	3.254925	4.472481	2.955028	6.157267	3.734885
2.861377	2.943408	8.50167	4.524889	1.802708	2.46707	3.91231	3.298448	4.006254	2.9666
12.77349	9.714795	10.44714	5.580303	8.397724	5.514358	10.68925	6.642418	3.64274	3.592929
4.378474	2.536252	6.327191	2.387232	2.931691	7.045988	4.772741	2.903511	5.117231	3.500514
9.081471	9.754004	23.76505	11.04744	14.93121	20.85911	16.53818	10.80544	20.96021	11.4329
3.582108	10.79945	3.489886	29.52846	6.474163	7.842287	13.86521	13.11407	3.919526	5.350604
61.03887	30.70774	80.29391	27.31896	73.6809	106.3946	80.84248	36.56715	118.6942	32.35024
44.84212	14.87178	9.497466	5.778579	9.906953	33.44184	11.28553	26.96239	4.15106	5.632912
21.87689	17.81363	44.80793	15.34282	33.0627	43.02522	34.27352	20.24215	54.07662	17.43252
9.932426	3.281255	0.691346	4.25385	1.820371	7.954884	2.067745	5.896218	3.062439	1.445195
4.507514	1.945164	2.03318	4.758076	2.257428	5.278246	3.674654	2.404305	2.117896	1.976239
3.562683	7.641857	15.84665	8.416292	11.25027	11.02005	8.908441	11.86206	16.90749	8.73509
7.623503	3.099859	5.417915	1.928442	3.253709	3.372299	4.003281	3.78711	6.845807	3.87277
5.088402	2.789978	7.132136	3.651748	4.613086	6.532097	6.233221	3.142845	8.049186	3.629571
7.493295	3.5705	13.42702	3.721872	5.477256	10.73575	15.25727	6.15274	14.60454	4.862371
1.972474	1.280093	3.029594	2.753538	3.283335	2.680351	4.02845	6.537488	3.171239	2.141382
4.720988	3.891921	17.28038	4.473566	6.926285	7.980436	5.43753	5.751802	5.2726	4.41898
2.878465	1.881887	4.499071	5.693624	5.109723	3.991978	3.678956	7.816286	4.159579	3.3864
26.87707	18.74908	41.02209	16.69864	27.20721	40.15135	64.23255	19.47474	48.07007	11.18259
60.56749	5.026881	5.787163	46.66711	2.224879	13.1239	33.72214	19.31674	88.4563	3.219114
0.994428	1.082616	2.139647	11.72373	7.320098	14.18155	3.329844	4.246134	11.85519	1.076797
9.911446	6.441322	19.08397	4.688859	6.872121	39.32761	6.068792	4.340792	4.01688	12.78647
2.041958	2.547654	11.30661	1.819298	1.854623	12.58656	6.508896	2.2167	1.840848	3.67466
4.2638	1.58524	1.943422	9.713735	2.025579	9.103064	1.950299	5.028359	1.342277	2.280868
25.50253	7.903959	3.912873	6.544645	1.667911	2.00079	11.62106	2.438778	7.489322	3.993839
7.406298	37.72028	2.202413	9.200228	1.278814	5.664602	19.14572	2.95958	6.732451	4.73582
3.507601	57.62389	1.467731	11.09322	1.052043	6.375199	29.54129	3.137675	8.644354	11.68876
7.089119	11.93339	7.101243	3.716115	1.36274	8.76086	2.12572	4.252725	1.833952	1.133011
4.176024	2.778736	2.678196	7.527279	8.365623	31.10095	4.163448	3.137595	1.633114	1.825943
8.20524	2.15813	1.407687	5.713035	2.145078	2.255816	8.118159	2.138491	6.85185	2.022258
6.425471	5.222502	3.298331	2.866482	2.183282	3.185875	6.640654	3.296529	14.62922	3.452243
5.608311	2.040268	2.07166	2.114054	1.15531	5.149389	15.67914	6.968959	1.883463	2.20517
9.749157	52.61908	13.1286	6.142671	23.23047	14.83671	30.38534	50.58253	16.11959	15.9129
3.442439	1.325536	1.562611	2.490424	2.136946	12.95792	1.630393	4.391983	16.86186	1.204588
20.97184	25.96513	3.732783	4.451285	1.869845	88.85747	8.172981	57.93316	2.654811	29.03606
13.79692	3.568498	4.338782	5.402753	25.17449	2.748777	8.066609	4.767504	17.56888	3.348242
10.83648	3.483457	12.90231	3.199522	3.510733	5.415373	5.808966	4.325785	132.0124	2.729152
4.386391	8.234566	2.957474	3.183023	7.24887	12.27559	21.40984	11.73204	3.025656	3.730936
7.947413	3.659592	3.252176	2.680317	1.790539	2.341188	10.84915	3.558753	5.093627	16.56626
9.284369	2.444151	2.770909	2.355936	1.572688	7.697223	2.095708	4.241557	1.447863	12.2379
19.63463	26.71029	23.36426	35.40133	38.62021	114.064	45.66457	57.9697	22.62405	23.04696
14.01816	11.90908	71.61521	30.54751	26.60056	17.62224	28.65888	13.51356	8.566498	17.6439
37.21575	15.65321	5.148515	70.95045	24.46308	10.34208	23.70853	86.18872	12.05844	15.42025
4.776213	3.542782	3.858418	10.81245	2.838594	3.537157	3.946821	9.346688	4.061842	6.305213
5.613113	7.230125	6.63272	10.74253	6.999892	7.417598	20.46027	11.74417	6.005635	6.308698
2.208399	8.470023	6.453734	12.94657	4.387285	3.239109	5.622263	3.60997	3.681442	4.648975
8.2605	22.15066	19.97871	23.92656	13.01091	24.34389	41.12063	70.97608	12.43668	8.822877
5.60873	10.58599	37.7999	35.34876	8.687192	71.97623	10.11379	25.36366	30.23756	8.234646

4.944123	18.37067	5.239608	6.430427	3.118846	4.857185	7.287646	22.5855	14.83896	4.44312
13.76365	11.64555	25.78915	15.9482	24.46442	15.12758	6.4484	13.60458	12.81477	8.021327
52.27961	12.83227	53.95361	14.27692	31.67563	27.39256	44.46132	20.71941	20.92817	13.23289
9.283715	4.393115	5.425002	10.58615	7.479852	39.40665	5.889147	10.76175	6.054341	8.757193
5.54717	3.686188	19.51031	2.944237	4.692557	7.131751	4.087384	10.90054	8.066475	5.992421
10.59737	5.720145	10.51173	10.86658	7.917981	29.59229	10.79427	10.42037	8.218703	8.316567
4.755073	4.323656	5.349351	8.403094	2.474837	8.673941	2.269501	7.10403	3.018837	5.826784
3.629688	7.017484	15.94581	3.787253	5.478761	5.422235	7.352867	8.104581	5.913382	8.270097
14.1478	14.51064	16.58683	7.616887	9.366406	33.92952	13.75207	7.916254	10.65864	7.931241
4.995759	10.14852	17.17737	8.161382	18.42941	38.27971	9.168195	36.25949	13.19337	6.458341
38.12684	12.98044	12.9661	9.372784	10.35287	15.22962	17.10251	21.40255	5.631295	6.583397
13.88482	11.37545	10.38386	16.38284	8.732641	3.260522	18.60187	8.552654	5.367961	6.018787
8.308765	2.940117	2.799837	3.327048	2.733623	2.284308	13.20472	6.068009	12.52143	4.300963
24.61881	12.58007	4.121021	12.12218	11.62429	12.42951	12.66509	30.25229	13.87118	5.891147
5.722852	27.9259	11.04095	6.72193	12.19673	7.165185	13.51047	14.64413	13.94974	7.545206
3.83414	5.894295	11.96139	5.183901	6.744555	6.438788	9.510095	4.80861	9.186648	5.9703
3.746053	7.80132	4.453054	6.31075	4.476303	5.46486	9.626611	8.049576	6.62714	5.249732
10.90916	20.42772	14.17141	55.43059	16.46912	17.61144	41.58155	33.10398	14.4387	13.73521
19.90674	14.4354	24.76396	53.73598	18.27304	9.348199	15.13235	30.94589	18.6138	8.569364
3.046032	8.652797	4.287419	14.8694	16.24741	11.92065	8.418858	13.02063	3.005165	4.919902
18.84434	16.34468	9.480439	9.540046	16.56342	17.76253	34.50955	23.19458	31.60621	13.87127
4.106175	25.52329	6.264038	6.282031	17.80427	19.49874	8.104134	15.57588	7.63417	9.206339
14.74021	41.802	16.7339	13.19187	29.05935	17.3343	37.83562	78.90905	19.18703	9.771489
15.10811	69.3702	46.35464	38.36207	13.95602	22.55032	81.43388	21.45559	50.08075	15.3658
3.1361	4.621689	6.461545	3.670053	3.638662	5.581005	5.766367	11.07632	12.10573	10.05174
38.94929	74.73215	9.916832	43.41614	31.42287	48.27118	52.22454	83.12383	35.83574	16.1347
23.01504	24.37037	23.28053	26.88675	19.21471	129.3814	31.82978	38.18625	24.49333	16.48197
3.554364	3.197927	1.32799	8.143245	3.236435	3.119637	2.808448	13.41105	4.609761	3.690261
7.782911	9.532385	4.21922	7.793817	7.230581	32.66258	9.563071	17.06406	11.55862	10.19485
3.72719	20.17746	9.383495	53.1646	10.4968	11.37005	32.97904	40.84642	18.21921	7.397121
7.235144	24.09006	229.3994	5.56104	7.014809	44.67887	19.44051	7.184196	9.332138	19.36967
24.4087	31.70429	39.53354	63.32092	13.1637	108.5666	12.36759	108.2067	18.8577	8.732802
13.80389	18.92501	22.91593	19.05788	25.25323	25.28758	34.43297	14.60389	17.34053	11.84302
56.50568	13.27008	28.82919	17.37354	23.58523	11.93584	53.71359	20.84178	11.19177	11.22901
2.574458	14.11737	45.20056	22.55539	12.06457	4.495503	3.489504	4.622007	8.42845	8.196513
3.234176	7.123436	2.152814	5.459941	20.11809	13.38149	1.622035	10.54338	11.27077	11.1161
4.357109	6.142012	22.28529	4.356595	15.53331	21.99362	9.873299	7.495643	9.90482	4.553716
1.441076	1.927909	4.237624	3.643154	1.984285	2.136197	2.559732	62.51571	2.207541	4.190541
3.62212	3.449384	25.61019	4.737609	6.568352	2.985514	9.922698	4.899516	3.288026	3.737528
3.190029	5.065603	3.115512	2.871778	2.663348	2.376131	2.859107	18.40307	2.205479	2.506504
5.868456	4.251155	4.918545	1.826311	3.295581	12.85098	9.053048	103.257	11.19512	2.996654
12.95612	8.289844	7.257577	6.573176	17.19111	13.69928	12.53872	41.58774	8.253275	4.32869
28.35627	36.61053	27.82773	67.84872	50.52502	78.23074	53.76415	34.60313	25.93893	18.30655
19.28779	22.73254	17.71985	14.32385	27.26058	17.04324	56.16215	34.32542	19.44976	28.44389
42.912	55.38954	35.74993	43.91244	35.17187	45.17907	34.63182	44.08656	38.51514	16.59606
3.514949	8.331262	5.004961	5.499043	4.731867	5.754188	2.84524	7.261798	3.306578	4.860032
18.15199	8.408916	95.66441	15.65731	6.763519	1.512725	3.266295	4.817281	1.374629	2.828519
27.8239	56.07674	11.63308	17.7942	23.42564	9.37094	69.20608	100.2421	13.8174	10.20589
57.35416	26.20734	14.63849	22.96933	23.74212	14.31934	22.37579	61.90789	10.7321	17.34901
20.27338	10.87901	25.34394	13.45081	11.15135	4.283533	7.047684	19.14796	7.447016	8.32187
12.75735	12.58979	116.5703	5.187068	19.5082	10.537	13.3096	24.89221	9.000836	5.864763
8.353707	6.061409	3.316677	8.247333	10.62837	7.534628	8.239674	4.482482	7.426638	2.242066
5.384962	2.943061	4.880207	20.74406	19.41495	6.80309	10.20912	8.342174	6.39449	2.81951
34.33559	20.94264	54.34823	35.76349	16.57859	9.499516	15.70955	35.44752	32.13234	13.51536

31.37877	13.22418	31.08891	23.10601	14.54649	6.803116	13.96978	22.82495	18.6388	11.16663
2.040515	2.07271	3.318921	3.200462	1.775522	5.363592	3.363676	5.129921	3.680586	4.039964
65.39784	11.49292	7.986799	5.628262	12.47021	12.16011	26.69061	15.17096	12.03283	9.517866
4.898608	6.860497	27.19613	5.677753	12.99361	4.414426	5.349467	5.357353	6.246805	3.959336
14.7124	26.30111	12.43281	20.80903	9.00186	7.788023	7.898423	37.00078	33.29282	8.473475
33.84947	17.99151	25.09538	12.43355	34.46825	31.52055	78.72801	24.56472	14.31636	13.50912
21.68695	30.54305	10.35172	42.22417	37.18201	27.47238	25.33773	117.652	61.28767	9.117072
1.628234	3.019036	6.285532	4.141772	2.137367	2.501086	2.793883	6.59964	2.545906	3.280616
5.646433	7.947273	16.82495	14.88907	4.198752	6.233345	8.294262	17.89298	6.391358	7.743115
24.09088	19.46557	28.48306	19.61127	42.73794	26.33433	44.98669	21.71089	45.50518	16.70557
4.985063	6.49362	6.707636	32.15047	6.409626	3.333356	12.14326	6.090881	4.412999	4.599961
2.330798	4.27559	6.341596	5.750272	6.30509	10.33019	3.925328	77.47629	3.566188	3.136306
32.64701	19.39761	15.37354	13.80067	45.07658	15.00545	34.95897	17.10018	21.18754	11.62599
129.3602	15.33738	31.74917	8.661531	19.05105	78.73813	58.4905	61.65658	22.16018	16.43139
37.99452	36.47562	41.04451	36.62817	18.27047	17.76684	20.68317	27.80379	24.2977	15.6057
12.1189	35.20001	13.66997	24.35212	30.32998	10.13125	37.69397	61.59003	9.404644	7.922125
10.92744	7.888694	12.67107	17.8817	11.68606	6.221573	10.6444	8.623748	8.140244	5.420659
11.2432	29.61597	27.35698	33.50105	22.21012	58.93233	21.41193	24.76189	16.62153	13.80307
9.100129	2.016446	11.4656	2.810776	2.708825	3.448179	6.329297	2.892687	9.252058	3.210291
3.072605	17.67262	3.137505	10.16487	7.187257	12.68773	5.897709	14.4106	7.524224	3.794687
2.290007	10.41784	1.790218	3.136157	6.404406	6.053976	3.441568	6.096882	7.334132	1.844945
19.2309	19.58222	30.36469	45.1952	29.24672	20.95443	22.77926	24.47209	40.028	14.53407
2.646101	17.80133	26.66667	7.120953	3.463798	6.039664	3.952233	16.07119	3.912893	2.420997
11.14025	6.918226	17.32317	4.370523	4.430371	8.111016	11.6028	11.67829	4.630123	3.531462
18.20139	26.04173	19.57913	55.38895	13.76053	14.8915	4.352363	57.05403	7.309828	6.773931
5.587552	2.302675	3.325436	1.588849	4.365791	33.54072	5.190928	3.108819	8.328773	3.679049
16.33066	2.459967	8.668791	7.705741	52.86838	4.033776	8.044801	31.77373	0.270839	2.58344
7.608929	10.32738	7.653217	22.81715	2.673074	7.220442	8.390081	23.45188	8.704767	5.240437
33.45573	19.64099	8.949343	38.58141	19.39673	7.467737	13.31482	41.67477	20.63845	2.829317
36.32521	20.52756	7.472252	34.71904	12.9872	6.048775	14.15179	41.56761	22.06912	2.565382
30.38927	5.91059	2.335734	7.452201	6.844783	11.88713	29.67511	31.37879	2.918826	3.219751
7.253358	12.92409	30.35751	20.57397	33.42047	16.36754	13.13246	29.54736	13.21887	7.142519
10.25676	2.783226	3.688233	27.0285	4.110834	7.774713	10.0834	4.328434	18.93028	4.208708
6.44395	1.986862	1.983642	20.30032	3.619457	5.451905	6.716999	3.327878	12.47501	2.842881
15.68833	29.0387	39.58645	11.45871	16.91642	8.454512	33.9062	35.98243	28.68217	10.75782
9.247915	10.38333	63.59158	4.809906	9.37728	4.40749	20.50502	18.45589	20.26359	6.549375
11.59411	10.12646	76.92851	35.87535	18.05809	87.74713	51.89281	25.30645	56.73371	8.523269
53.54111	23.22703	52.62774	66.55327	19.32027	24.147	30.39374	56.8841	22.90214	30.48114
5.176276	3.926192	2.725339	11.2074	3.302585	4.529471	3.798017	8.984041	2.291308	2.52394
8.179026	6.678087	3.776455	24.27826	13.55803	5.336107	13.5877	10.26118	5.329055	6.996463
30.50579	10.86354	40.43654	43.14917	14.45319	9.172207	11.47727	25.63762	20.81652	14.19836
3.81066	3.570931	6.160037	4.86712	3.146314	5.404862	5.405665	11.83723	8.941841	4.503573
20.83345	19.78048	10.32759	9.960379	10.66273	8.013947	15.38745	19.70403	20.50233	9.278446
15.85517	30.36638	29.31563	17.57948	25.50117	59.18214	12.261	23.57351	13.67514	9.954696
13.96861	10.96632	10.27852	57.65347	11.58721	13.61299	18.06197	69.215	7.163961	4.95673
43.05682	11.09958	46.4739	48.01679	9.990242	30.46812	9.931543	32.20525	8.531063	4.334996
5.093974	16.64455	2.46751	4.434314	11.01116	28.93142	2.097018	15.74379	5.100792	1.668767
25.51119	16.18991	10.16573	10.33036	9.152929	8.785861	15.43503	37.07728	14.62517	7.152915
3.938878	4.829837	24.68244	21.52353	9.610738	42.33438	17.40506	13.54542	31.85375	4.606175
4.043454	10.89061	9.33905	2.61869	6.928068	23.33827	9.481202	2.455681	2.388769	5.307755
15.06724	6.721964	121.0562	5.986416	15.69711	16.2588	12.36486	54.17781	4.833259	4.445078
16.7051	26.74749	17.11061	38.79133	32.09717	18.81775	29.18223	31.71213	7.971386	9.815463
5.946502	6.842062	5.860249	21.92716	11.96585	5.339105	12.36287	8.521098	3.428215	4.545285
7.796229	12.54603	12.41511	5.527956	12.27525	4.425086	13.03894	5.998815	12.27764	6.380187

5.506643	5.377178	3.943362	5.532848	3.933386	2.501023	5.384317	6.041381	2.9221	2.396194
17.02647	63.91708	18.30803	7.768141	7.274699	16.77412	28.98116	32.20485	11.71535	5.765495
27.49883	9.087028	6.098515	8.18369	6.990762	37.91549	27.19741	17.34381	14.44981	6.249521
6.668763	4.11129	6.172257	4.446816	5.580681	2.426346	4.649669	5.729511	4.558559	3.56229
14.94679	22.21655	17.85805	17.92439	14.52437	5.867822	31.29452	36.12833	6.438399	7.381247
7.246783	6.174492	11.92469	8.459745	18.18282	12.63677	15.29862	90.49779	4.842767	6.440083
3.721578	3.236379	13.33183	30.18629	10.19707	19.00776	4.672362	12.76031	17.24407	8.306155
5.328467	9.611924	5.814117	9.083556	17.09779	10.27315	6.031017	12.67436	3.893531	4.529505
26.7087	24.29608	27.62292	15.8486	31.12735	48.79045	16.7744	37.98652	18.85766	9.319463
7.19956	8.878621	12.99827	24.00868	18.89747	5.911688	29.97945	39.21226	7.610712	5.466003
14.8998	29.2223	35.60098	58.83136	77.06096	21.50383	48.52148	38.03209	25.91359	14.13319
3.777046	5.844591	4.233403	6.8258	16.03433	19.7702	6.642213	6.316455	4.575318	3.554112
84.19059	2.018054	1.357193	1.058543	2.206503	1.588561	34.41477	1.83002	7.846502	3.056813
7.905012	36.86864	9.725819	12.05922	30.81649	5.127472	8.004582	11.84157	12.80546	5.269795
32.77667	23.11338	22.8387	19.35696	13.15216	16.51506	34.53055	19.87216	26.72605	13.47266
10.22282	16.84163	15.09921	7.704663	18.33618	11.90385	34.64532	14.17735	27.70614	7.767019
22.79615	11.15081	31.72887	12.55204	29.35966	22.45282	44.18557	152.8325	8.632856	12.64812
11.3196	11.25994	9.372675	10.09072	17.06522	16.37163	17.69179	18.88903	13.97201	8.43087
4.703746	3.270474	2.67936	8.00921	12.19877	1.70718	3.550062	2.052051	5.561358	3.552201
29.77009	17.20367	11.70614	15.14359	16.71859	8.708129	20.34074	41.74667	13.24167	9.152299
5.941823	4.192566	3.205224	12.9151	15.54787	2.995562	7.009166	2.918151	7.922825	4.913355
4.3617	5.333409	13.40962	17.95524	3.479806	5.280687	21.37644	9.165426	4.667067	3.383596
4.763728	7.928323	5.30053	3.310211	5.97899	2.889043	6.08741	4.418212	11.82555	5.405015
1.41167	11.14765	2.128053	13.25229	19.06263	3.9725	2.370688	9.197137	18.59716	4.932836
6.896902	8.223245	59.51003	5.527128	8.723537	4.638266	19.82837	17.53718	17.88881	5.845996
14.70934	31.16574	14.1273	29.48987	7.913789	3.606799	4.510756	55.28995	22.9051	5.015918
3.732518	3.81737	3.976837	17.74929	18.01103	13.1558	9.538259	23.77027	4.577409	5.467522
3.056607	17.52126	23.23963	4.162352	1.771618	4.014245	2.514689	11.42054	2.597121	1.231156
2.932556	4.880293	17.21385	6.863682	11.82988	8.108784	4.515111	8.189566	5.808402	5.235748
2.863561	4.45742	13.08676	6.744758	5.673027	2.71394	3.930386	20.95736	3.324041	3.548477
61.86029	12.21267	39.82247	12.03966	17.93816	29.98018	20.83609	44.02331	10.06732	9.812216
10.70275	5.8653	56.10949	11.23927	6.172978	7.232318	7.940969	12.94384	5.142513	6.829426
7.886536	25.20233	62.5	26.50858	6.938647	10.16006	13.92665	38.5319	9.701794	3.893958
132.8659	31.66305	17.84232	15.21393	20.68616	39.63152	19.55481	34.88352	28.21634	7.525437
3.31414	3.590377	2.178218	5.2565	7.804974	2.760625	9.624043	4.712453	3.48477	3.651069
4.631665	4.541862	7.175075	8.220778	15.25946	4.658028	15.15435	6.334672	6.828443	4.392358
18.34483	11.28643	2.106492	6.816135	2.84209	29.66996	8.751553	16.38327	5.875404	5.241846
10.7361	21.9243	5.477425	22.126	25.56849	11.08508	32.46328	66.6013	5.499256	10.54845
4.319839	2.670059	14.05517	5.650945	2.098983	3.934345	14.14463	4.010835	9.122468	5.84256
22.7837	15.73194	21.72667	27.94906	25.18189	72.74402	28.65213	16.00845	12.23516	10.09242
6.405079	5.619596	29.42564	21.16733	14.02772	8.683284	11.79493	14.65512	2.532162	3.995246
11.91545	17.71082	37.21383	48.92982	23.22085	12.84341	23.42632	20.69544	41.87312	11.65344
20.48485	14.59574	27.17242	43.59143	23.72621	10.21338	17.83234	18.8674	26.45029	10.11076
5.423822	3.60987	9.489576	5.518446	15.6715	3.998854	9.429692	12.32535	4.689816	4.476224
3.396904	4.604488	3.691969	9.309769	4.920058	1.720746	3.255945	8.154698	14.88465	1.293463
3.546946	10.46131	26.16242	32.72705	16.18174	10.35935	7.035706	19.34463	7.103519	4.193798
77.46842	35.66899	36.7531	49.34134	24.70516	39.52332	78.39505	80.78277	50.46277	39.34927
68.67471	40.63843	24.92794	50.50035	25.7005	47.74135	41.78416	43.41641	16.25163	21.72
5.759055	4.057076	3.972326	7.673206	7.134436	3.151447	3.913776	5.064616	7.791901	3.327821
15.85495	10.09673	8.928241	11.5675	4.133183	5.423228	2.465597	103.7738	7.913028	3.221812
47.02158	5.232996	1.397234	34.41214	2.790074	5.026104	6.30908	7.043163	2.169595	1.814436
8.170742	5.324976	10.41254	9.566039	5.710057	3.765763	12.61529	23.19192	4.729781	3.972045
15.21221	20.17247	21.60331	13.31954	25.50781	17.66177	35.75599	109.9662	34.46229	12.5967

HC11	HC12	HC13	HC14	HC15	HC16	fold chang	p-value	cutoff	positiveRC
19.6099	20.24902	13.03653	8.717585	22.83842	5.823081	2.194352	0.000277	26.2835	56.25%
3.295525	3.199558	2.125336	1.669353	1.56537	1.895806	1.764877	0.000619	4	31.25%
1.699717	1.562724	1.599016	1.168377	1.793452	2.184029	3.054507	0.000619	4	50.00%
2.101756	4.743835	1.350123	1.379214	2.874357	2.687279	4.265627	0.001124	6.714274	50.00%
1.466661	2.256769	1.53014	1.249131	1.661137	1.217563	2.354172	0.001296	4	37.50%
3.16144	3.0105	2.065691	1.689748	4.102484	1.981832	2.351027	0.001492	4.102484	56.25%
1.647965	4.26975	1.701776	1.599459	2.866638	1.172529	1.870451	0.002242	4.46612	31.25%
0.542868	1.884678	1.085668	0.744303	1.873736	1.281941	2.932618	0.002557	4	43.75%
1.079259	2.004014	1.41708	0.771719	2.337803	1.05545	2.884753	0.002909	4	50.00%
2.970598	2.635344	1.318265	1.850124	1.638326	1.481975	3.566868	0.003303	4	31.25%
7.782192	4.645167	5.673615	3.272287	5.015628	7.550289	1.548551	0.003303	9.653455	43.75%
1.435643	2.535146	1.44953	1.060468	2.717425	1.34776	2.602851	0.004232	4	43.75%
2.131934	7.918354	2.372779	2.081409	3.864885	2.377781	1.589123	0.004232	7.918354	31.25%
0.682516	1.76392	0.844543	0.705168	3.720994	1.132239	4.290544	0.004775	4	50.00%
1.903924	5.149462	3.742849	1.667609	11.7023	3.179149	3.833653	0.004775	15.7059	37.50%
38.8115	16.41067	7.310572	7.404636	10.0926	6.55274	2.25054	0.005378	30.96393	43.75%
4.184979	12.29077	4.416946	1.834062	23.85751	2.393559	4.113753	0.006045	23.85751	50.00%
3.617133	6.84614	2.207448	1.333161	8.550727	1.60099	5.166123	0.006045	12.75037	43.75%
7.811908	6.351317	5.590078	4.649347	11.07862	2.67164	1.844621	0.006045	12.32673	37.50%
1.587028	1.727868	1.960462	1.38235	2.112832	2.018524	1.808918	0.006045	4	37.50%
5.026143	3.103025	1.35944	1.74249	2.026272	1.722559	2.160655	0.006781	9.597195	37.50%
2.111183	2.87985	2.01608	1.440153	4.530236	1.26291	2.086158	0.007592	4.545318	43.75%
3.7148	2.124418	2.76201	2.172953	1.704758	4.199809	2.189371	0.007592	4.199809	37.50%
12.20758	2.396471	2.197363	1.438972	3.356737	2.122709	2.573932	0.008485	19.65422	31.25%
1.782548	2.205445	2.312898	2.332475	3.218665	1.676182	1.986387	0.008485	4	37.50%
6.788564	13.13456	2.32131	4.757989	4.545423	1.348183	1.915983	0.008485	6.788564	50.00%
1.886998	3.255012	1.434078	3.349094	1.890446	2.12375	2.703039	0.009466	4.550601	43.75%
3.689052	2.914729	1.541249	2.061038	3.314915	1.510134	1.819525	0.009466	5.177914	31.25%
1.817114	2.468897	1.174624	1.887714	2.936429	2.026888	3.829547	0.009466	4	37.50%
9.425377	4.777126	1.576424	2.226966	2.458369	1.378987	1.602172	0.009466	4.777126	37.50%
1.001858	4.046755	1.123919	0.805579	3.482935	0.627691	3.742794	0.01054	6.603467	43.75%
11.8325	6.745377	2.818661	1.969157	2.394433	2.073349	2.056183	0.01054	6.745377	37.50%
38.55248	20.68213	1.138574	4.051718	3.729826	1.328261	3.209605	0.011716	20.68213	37.50%
1.073485	5.011603	1.262174	1.172328	2.145101	1.927121	2.505143	0.012999	5.011603	37.50%
3.700952	3.674619	2.006536	1.209487	5.16692	4.685848	2.823245	0.012999	5.16692	56.25%
9.513483	8.112477	2.448477	2.529973	3.473865	1.784828	2.347028	0.012999	9.513483	37.50%
5.646814	12.56903	2.347478	4.23108	5.424461	6.632702	2.819323	0.012999	21.52768	56.25%
2.686744	7.107985	2.386019	2.456766	5.435199	1.105764	1.528964	0.012999	5.435199	31.25%
17.42617	49.74709	1.346774	2.044373	29.00812	1.164847	2.882994	0.014398	30.66781	31.25%
0.976916	2.138344	0.864005	0.708528	1.943303	0.831648	2.849685	0.015921	4	31.25%
1.665237	3.183759	1.092467	1.169417	1.701293	1.792232	2.150264	0.015921	4.021965	31.25%
3.162897	3.003174	1.873308	1.832627	5.778599	1.710864	2.465436	0.015921	7.221607	43.75%
1.22301	4.1056	1.306979	1.449432	4.580502	0.618055	4.139275	0.015921	9.019146	43.75%
4.995263	3.295289	4.348006	2.435619	2.004775	4.673194	2.243549	0.017575	4.995263	43.75%
3.136426	3.962973	2.397896	2.733113	3.009629	2.351699	1.879009	0.017575	4.602651	37.50%
2.006287	3.163221	1.678552	1.708201	2.96337	0.809603	1.518133	0.017575	4	31.25%
9.509257	5.066441	2.177911	1.548	2.485276	2.626375	1.839974	0.017575	5.288023	37.50%

9.752186	29.71423	5.977457	4.172928	26.68461	4.498952	1.806546	0.017575	30.12722	37.50%
3.537553	1.378926	3.885296	1.892333	2.122785	1.68542	1.674021	0.019368	5.280918	31.25%
1.003018	1.315459	1.060504	0.768007	2.002519	1.395179	2.010874	0.019368	4.490897	31.25%
10.16469	5.799725	1.755593	1.599271	2.290165	2.754524	2.011976	0.019368	5.799725	37.50%
7.75877	7.670033	12.63883	0.897597	3.377428	1.667252	2.787134	0.019368	12.63883	31.25%
2.285546	1.303909	2.337763	1.236154	3.846964	1.907335	1.542257	0.019368	4	31.25%
1.93784	2.511371	1.778798	0.990604	6.336579	2.307628	3.398416	0.02131	7.413702	50.00%
3.262853	4.171476	1.794646	1.986665	4.898283	1.078461	1.676362	0.02131	4.898283	37.50%
4.677863	5.918755	1.907399	1.678463	4.40152	2.801345	1.68222	0.02131	5.918755	31.25%
1.795291	1.568716	1.302311	1.5738	2.468467	2.703661	1.894283	0.02131	4	37.50%
4.2106	8.267757	1.790684	2.199397	3.084338	2.143815	2.306423	0.02131	6.729541	31.25%
3.424704	3.297072	1.939399	1.58782	5.45146	0.887856	1.906396	0.02131	5.45146	31.25%
6.700053	3.999496	3.285331	3.715275	2.985611	3.014379	1.522974	0.02131	7.070428	31.25%
21.24789	8.072481	8.580675	13.69959	8.497793	4.275636	2.407925	0.023407	21.24789	50.00%
1.437529	1.657272	1.938635	0.988	2.953973	2.275128	2.715243	0.023407	4	50.00%
13.74373	13.64025	1.95562	1.704281	12.34628	0.690377	3.497303	0.023407	19.62764	43.75%
4.646097	17.17731	5.294288	13.48883	3.910799	3.244621	2.921672	0.023407	19.06897	37.50%
9.054043	7.07669	3.190525	5.029613	9.247611	1.962207	1.549958	0.023407	11.19052	43.75%
4.072833	5.712812	1.904685	2.048704	2.50903	2.955112	2.648474	0.023407	8.153121	31.25%
3.861831	18.62763	4.897921	1.830245	16.12928	4.59768	4.131715	0.025671	18.62763	50.00%
2.873637	3.12105	2.262821	1.71875	4.765067	2.101836	2.397203	0.025671	5.755034	43.75%
1.446233	2.399925	1.2312	0.980727	2.406083	1.539299	2.365332	0.025671	4	43.75%
12.41814	6.091109	3.712534	2.87736	3.389718	3.92173	2.861375	0.025671	7.171932	37.50%
3.917752	4.160901	2.406604	2.831541	6.498694	1.54496	1.642136	0.025671	4.862099	50.00%
48.32394	73.76656	14.51035	11.87322	18.51115	33.94396	1.637874	0.025671	48.32394	37.50%
49.57819	14.45985	47.45234	13.40338	24.22663	16.39621	1.739953	0.028108	74.96889	37.50%
1.562343	1.751369	1.832504	1.363265	2.594675	1.572479	1.866994	0.028108	4	37.50%
3.264644	2.885708	2.133242	2.58397	3.454256	4.593085	2.589897	0.028108	4.593085	56.25%
15.21382	27.75956	13.68742	4.688667	9.553348	14.65065	1.615871	0.028108	17.66751	50.00%
3.973555	2.977582	1.423017	1.183137	1.836654	1.486124	1.581666	0.030729	4	31.25%
2.736948	12.4534	7.046807	3.417941	24.33991	7.497841	2.802872	0.030729	24.33991	56.25%
2.730658	2.901376	1.729577	1.933249	3.403527	1.655371	1.619239	0.030729	4.262892	50.00%
1.033729	3.775508	1.596478	1.20993	4.294773	1.892553	2.986831	0.033542	4.939784	43.75%
1.074325	5.602639	1.535678	1.034222	7.660039	0.720102	3.801687	0.033542	15.3699	37.50%
6.918627	4.605442	1.51855	1.998688	1.569983	1.856564	2.030361	0.033542	6.918627	31.25%
5.795464	1.386695	1.016908	1.28336	1.804779	1.33436	2.228178	0.033542	5.795464	50.00%
8.73385	3.624515	1.769425	4.051259	2.560787	1.576368	2.439234	0.033542	14.72865	37.50%
3.598368	7.174786	1.951691	2.398996	7.200186	3.328042	2.324577	0.033542	7.200186	37.50%
12.77127	14.02805	2.091286	2.46203	7.484567	2.297924	2.274683	0.036558	14.42407	43.75%
2.08929	6.384851	1.438457	1.278186	2.820429	1.621096	1.916033	0.036558	5.405246	43.75%
2.809167	5.074073	0.845945	0.782076	6.648419	1.894267	6.341049	0.039784	11.57022	43.75%
6.651337	10.32736	2.54284	1.546654	4.228293	1.881633	2.709021	0.039784	7.161481	50.00%
6.154901	4.981765	2.704967	1.109914	7.784219	12.18866	2.937999	0.039784	12.18866	50.00%
4.81917	4.277045	6.462913	1.867826	6.130036	3.475323	2.090624	0.039784	6.462913	62.50%
6.414369	3.357693	1.222119	3.261471	1.665662	1.307695	1.96269	0.039784	6.414369	37.50%
0.811047	3.909778	1.137261	1.004441	4.108799	0.798481	3.591903	0.04323	7.890217	37.50%
3.556172	2.241629	1.348842	1.984107	2.003653	1.730949	2.864933	0.04323	4	37.50%
13.25933	7.910219	3.164416	2.308181	4.492961	3.13617	1.83467	0.04323	9.510595	31.25%
5.793098	3.140927	0.880542	1.548977	1.525859	1.023512	10.70411	0.04323	4	31.25%
3.570228	2.808343	1.10403	1.188643	2.114618	0.669535	2.440541	0.046906	4	37.50%
5.809251	8.185222	1.715849	4.683185	3.590879	3.364595	2.380845	0.046906	8.185222	37.50%
5.456173	2.74364	3.490449	3.97815	8.076179	3.782895	1.511885	0.046906	8.691321	37.50%
1.890819	1.919103	1.088368	1.112201	2.267307	4.550427	2.163716	0.046906	4.550427	31.25%
7.85308	6.446867	2.370345	2.473183	2.551906	2.931981	1.543464	0.046906	7.85308	31.25%

2.163901	5.280047	2.331385	2.566654	1.562535	1.83465	1.700867	0.001962	5.280047	43.75%
6.171334	15.77772	9.601	6.824599	8.151823	9.357432	1.732039	7.86E-05	15.77772	56.25%
14.12509	24.91725	27.90942	18.01557	12.88377	13.63053	1.967998	7.86E-05	42.56052	50.00%
3.220766	7.580754	6.509197	4.30405	3.34704	4.108889	1.565275	0.005378	8.558667	43.75%
4.556809	10.0495	3.107233	2.825606	3.293278	2.357215	1.573981	0.002557	7.131598	43.75%
7.889264	11.7672	9.822761	9.717465	6.764452	10.40948	1.50082	0.028108	11.7672	50.00%
3.184936	7.040709	3.046383	3.816465	2.67587	2.772221	1.668949	0.001124	6.157267	37.50%
2.529563	3.787256	3.016538	3.917193	2.222834	2.674841	2.060978	0.002242	8.50167	31.25%
6.116113	4.894481	2.117845	2.686232	5.223207	2.821439	1.763889	0.033542	12.77349	37.50%
2.054866	4.589164	2.918335	2.29027	1.965238	2.46227	1.546692	0.003743	7.045988	31.25%
7.736741	15.95291	14.68469	11.61968	11.92458	9.994408	1.502819	0.002909	23.76505	31.25%
5.814753	5.441809	3.814524	8.530245	2.750998	8.315159	1.931793	0.04323	13.86521	37.50%
20.41039	91.23001	72.34438	45.71581	29.38938	29.35787	1.846493	0.001296	118.6942	31.25%
5.952184	17.56437	8.505648	6.196237	10.99155	6.673872	1.991782	0.003303	33.44184	31.25%
12.60029	37.3214	40.76842	21.93429	24.05504	19.92449	1.603231	0.004775	54.07662	31.25%
1.581691	1.724534	1.097634	1.711502	13.03545	1.586966	2.831323	0.04323	9.932426	37.50%
2.16571	2.532095	2.38843	2.32452	2.020726	1.922113	1.514475	0.011716	5.278246	31.25%
5.516339	14.68372	7.035793	7.841952	4.645563	5.694904	1.634231	0.028108	16.90749	31.25%
3.152914	3.202816	3.576518	3.185918	3.129315	4.389984	1.686164	0.001713	6.845807	31.25%
2.575055	6.735517	3.906396	5.038184	3.850991	2.8013	1.545103	0.014398	8.049186	31.25%
3.650087	11.21011	9.595367	5.217046	3.797732	3.203599	1.607149	0.011716	15.25727	31.25%
1.964894	4.413546	2.34223	2.84518	2.614125	1.396192	1.518214	0.014398	5	37.50%
7.081703	9.75808	4.040972	4.148322	3.991098	4.067068	1.833161	0.030729	9.75808	37.50%
2.976782	5.81132	3.555489	4.083701	3.238037	3.205204	1.554436	0.023407	5.81132	43.75%
10.8164	38.46029	49.44302	20.54119	18.7594	14.95637	1.603288	0.023407	49.44302	31.25%
29.56091	5.045043	2.782689	2.101858	9.040085	0.773093	0.221004	0.004232	26.57164	0.00%
11.14678	32.13305	1.085947	1.148235	8.143751	0.809944	0.333351	0.04323	6.939062	0.00%
10.9515	10.40101	8.503664	2.445382	5.101291	9.451535	0.491079	0.004775	7.857723	0.00%
5.079214	14.74334	1.175811	1.924226	1.634627	2.149215	0.513327	0.023407	4	6.25%
1.422267	9.159654	4.946452	1.198205	1.088087	1.459644	0.412663	0.004775	4	0.00%
6.045094	4.215726	1.378385	4.080067	3.537867	4.443933	0.581592	0.036558	6.268181	6.25%
39.09644	12.75923	1.707521	1.703912	17.55037	5.424902	0.298182	0.01054	8.429595	0.00%
55.94082	21.82499	2.060747	1.667605	53.29442	7.890987	0.210919	0.02131	10.11142	0.00%
6.051105	1.026266	1.08002	1.449366	1.072639	1.189486	0.48789	0.030729	5.467308	6.25%
4.925149	6.659051	6.488769	11.11969	4.373188	1.698345	0.480744	0.012999	6.401368	6.25%
5.611514	3.950579	1.030705	1.449092	6.614976	4.813274	0.485656	0.001296	4	6.25%
2.671039	6.063304	2.123409	2.86739	3.808602	2.623151	0.568528	0.002242	4.136417	0.00%
2.000789	1.789151	2.01449	4.076089	2.390107	2.137257	0.415247	0.000385	4	0.00%
48.47814	15.07945	5.241087	12.7787	8.931377	2.531371	0.582776	0.036558	19.43672	6.25%
1.356645	1.346325	1.271032	4.582424	28.53002	1.551627	0.483615	0.023407	4	6.25%
11.82872	9.130767	2.058816	3.323557	73.33371	2.012598	0.217099	0.046906	12.35896	0.00%
27.81736	3.384783	14.5848	2.129866	2.491655	1.799434	0.605981	0.017575	11.85918	6.25%
3.91369	9.410308	1.619424	11.88862	6.1232	4.920219	0.285022	0.017575	6.343663	6.25%
14.00827	11.49809	4.549159	2.082045	7.107456	3.577969	0.582799	0.003303	11.36165	6.25%
38.52539	15.38109	1.600804	4.78418	2.153661	2.526917	0.433264	0.015921	7.729917	6.25%
4.834478	4.886925	3.138579	1.142464	8.326802	1.840276	0.479401	0.008485	4	6.25%
137.1428	56.01339	18.25633	18.06553	12.42077	26.23535	0.556569	0.046906	43.92484	0.00%
13.34095	16.64535	4.678582	30.37901	12.38623	14.66917	0.588819	0.007592	24.8682	6.25%
11.06337	43.65853	3.6297	3.615173	34.58191	5.762389	0.278536	0.001124	19.51765	0.00%
24.99825	5.317009	1.659373	2.850883	2.273223	2.876543	0.463738	0.002242	5	6.25%
7.83602	14.63043	3.170025	4.161396	8.286742	8.526728	0.654335	0.006781	8.382838	0.00%
8.405963	4.686912	2.545385	4.305485	9.522715	3.730642	0.595229	0.006045	5.838304	0.00%
34.92402	10.15195	10.00223	27.56716	15.3843	26.61369	0.666622	0.011716	23.96381	6.25%
9.795858	21.24692	5.147879	8.17651	8.496274	60.13667	0.58307	0.007592	14.16784	6.25%

6.433262	7.61648	2.926402	8.17526	8.770405	4.293752	0.599856	0.017575	7.165966	6.25%
6.621406	14.57079	8.176712	4.811214	7.901857	5.519519	0.594348	0.004775	14.26014	0.00%
21.88335	125.1638	10.19807	22.32005	15.67666	18.37317	0.444765	0.002242	26.19202	0.00%
6.879492	8.151705	16.93102	6.017184	4.694837	6.109355	0.547557	0.001713	6.467835	6.25%
3.411705	8.331235	20.98443	5.074053	3.751367	4.261355	0.516907	0.001713	6.495667	6.25%
12.75232	11.52556	15.62594	8.356235	5.02975	4.972077	0.629268	0.000721	10.11143	0.00%
3.735925	9.606273	2.647978	3.698311	3.260732	5.122901	0.600179	0.002909	5.071389	6.25%
8.950324	16.26952	4.865014	9.501572	5.497826	6.587695	0.65789	0.008485	6.791064	6.25%
13.84483	13.62162	5.483436	24.3243	7.278684	6.033274	0.522805	0.000721	12.75556	6.25%
13.02007	26.11041	4.356416	23.42285	12.54174	6.15818	0.514792	0.007592	17.59832	0.00%
11.04417	55.62906	11.20047	15.42206	5.686452	11.83183	0.601662	0.028108	13.55233	6.25%
8.146296	8.552732	10.90232	5.166911	10.5325	5.275163	0.653104	0.011716	10.86601	6.25%
22.7482	11.70129	17.70176	2.580392	11.14791	6.57953	0.392454	0.002909	7.771559	0.00%
13.34747	19.20608	7.199164	20.33636	19.10191	8.164811	0.499829	0.001124	14.11522	0.00%
14.63835	28.01135	7.298051	10.94922	19.76662	8.165598	0.617434	0.006045	14.21511	0.00%
14.3405	19.63658	4.868713	8.269405	7.420105	7.030872	0.64494	0.002909	7.563069	6.25%
6.290751	24.29405	2.29664	7.184866	11.72999	10.32454	0.621193	0.006781	7.61097	6.25%
19.60429	44.3585	7.586879	35.01672	22.7765	19.18943	0.628869	0.02131	30.135	0.00%
21.07062	20.12019	9.125896	30.64753	15.96413	22.17296	0.549069	0.000619	20.69005	0.00%
15.94171	9.769361	5.074119	22.0024	7.976057	10.88164	0.54883	0.006045	11.23013	6.25%
26.5254	33.47353	9.862483	55.54098	9.726864	17.88402	0.622836	0.033542	23.00512	0.00%
8.714961	122.6896	3.539217	5.458897	12.63777	11.3951	0.624835	0.033542	14.2121	6.25%
13.44414	48.84223	19.33022	16.02727	17.00084	7.267277	0.362777	3.53E-05	18.04577	0.00%
50.92815	26.87824	12.29731	62.095	18.82482	17.15212	0.508131	0.01054	40.73138	0.00%
9.200892	10.05025	4.30714	5.776564	4.263119	6.458715	0.648218	0.015921	7.073262	0.00%
99.62209	45.597	19.6174	20.38795	43.1734	24.96257	0.605022	0.011716	46.84532	6.25%
48.90491	97.77904	8.504619	16.08097	8.47064	20.48479	0.513734	0.025671	24.76412	6.25%
4.095707	24.02611	3.319022	2.231444	6.758824	6.890673	0.593635	0.036558	6.520649	6.25%
3.873676	4.0186	11.83289	27.9053	8.5448	8.51412	0.653734	0.002557	9.259433	6.25%
14.01097	16.30673	13.56373	21.08707	15.73738	8.747848	0.543035	0.014398	16.59461	0.00%
11.11054	13.92044	4.855954	9.355038	6.695153	6.976254	0.336061	0.023407	16.0101	6.25%
27.25883	59.6441	4.708111	13.15627	16.47673	9.945169	0.302634	0.00053	24.42554	0.00%
13.01167	18.59246	8.600746	54.16216	9.97023	18.53874	0.619748	0.003743	20.08008	6.25%
19.41212	42.85315	11.27441	43.53202	20.36247	24.86184	0.535133	0.004775	24.55804	0.00%
28.7559	57.53907	2.715813	3.638771	9.174944	5.598091	0.355927	0.028108	11.75208	0.00%
3.850049	5.675618	1.277748	2.53993	11.17602	3.083088	0.597402	0.036558	10.30288	6.25%
19.77661	10.64853	4.371355	4.70512	2.499341	17.07619	0.628918	0.046906	14.69692	6.25%
11.27539	5.451431	18.14175	11.10563	2.01957	4.055194	0.312229	0.046906	5	6.25%
6.561536	6.136323	2.873896	2.759535	2.516381	6.727892	0.568532	0.039784	5.055667	0.00%
9.157729	6.066057	6.715836	1.931725	8.131607	4.201934	0.548002	0.036558	5	0.00%
3.730099	7.869592	3.127782	3.467162	40.12865	10.9311	0.303056	0.025671	10.68797	6.25%
8.665033	9.417465	8.950775	15.44145	7.848501	13.76846	0.644783	0.012999	9.939204	6.25%
55.01748	48.98151	8.394182	25.87453	19.38234	30.26234	0.505173	0.001124	33.71866	6.25%
23.44895	18.31591	20.03632	14.70841	11.50805	26.75616	0.608804	0.000972	23.12195	0.00%
14.08709	44.12272	8.977578	53.70836	14.9696	42.03888	0.601662	0.003743	35.27533	6.25%
16.18492	7.752747	4.081121	10.03253	3.594911	3.95305	0.568946	0.002242	6.246743	0.00%
30.30395	3.130226	3.249028	9.133805	1.459838	6.561584	0.253484	0.033542	6.414013	0.00%
21.31406	46.20123	12.19289	37.82031	17.46339	12.50527	0.429856	0.007592	27.92524	0.00%
40.55245	19.88665	15.08574	15.48694	23.0519	32.19833	0.522773	0.000721	23.30347	6.25%
14.71779	19.58857	4.615809	7.006085	3.100948	8.454095	0.620277	0.023407	11.16723	6.25%
13.6344	32.90367	7.018201	21.96563	15.55007	19.77405	0.415265	0.002557	17.65661	6.25%
3.633976	7.969207	2.491436	4.200801	14.40665	14.07399	0.600499	0.014398	6.378089	6.25%
17.0953	10.28099	2.698017	9.66607	3.280597	16.00015	0.597194	0.036558	8.710473	6.25%
26.89002	21.48775	15.56034	29.72932	41.88716	13.88093	0.533168	0.001713	25.11598	6.25%

17.04731	14.71282	9.877355	15.82305	19.53559	6.162093	0.637329	0.005378	16.11557	6.25%
5.725514	3.665032	15.63527	7.237903	2.483677	4.374234	0.628663	0.02131	5	0.00%
19.89141	15.42255	4.641451	11.48169	6.625606	9.889844	0.417419	0.000327	13.99755	0.00%
7.582489	8.500768	3.158627	10.51583	9.726688	10.67714	0.664891	0.025671	8.82938	6.25%
18.45601	11.47842	5.156772	11.45379	19.723	8.410458	0.579045	0.02131	13.30608	6.25%
17.18309	46.83467	10.64813	20.2089	14.2271	17.11104	0.574626	0.004775	27.11683	6.25%
53.51718	85.28909	23.2774	10.86979	52.25734	32.00729	0.566549	0.030729	42.45212	6.25%
3.409684	6.232492	1.881486	2.425516	9.127577	6.997476	0.509681	0.001492	5	0.00%
4.875074	7.359829	3.168326	5.021258	16.63648	11.52742	0.562655	0.009466	10.43478	0.00%
22.83249	55.42542	15.30809	65.79216	20.10868	30.69241	0.610521	0.004232	37.78265	0.00%
7.730837	6.941231	3.499841	6.718575	4.488725	7.080894	0.627514	0.023407	6.842317	6.25%
3.686933	8.163314	3.729356	4.117388	3.707128	4.696053	0.367307	0.006045	5.95538	6.25%
35.7901	46.57257	14.47215	44.49114	31.43739	17.25623	0.627323	0.008485	34.50278	0.00%
37.43374	22.06276	16.80319	24.77656	24.64607	16.34293	0.563675	0.036558	37.0061	6.25%
26.25858	16.32677	27.02209	66.60231	33.62753	15.69639	0.609972	0.000385	29.39758	6.25%
33.9333	26.47582	29.1851	19.2364	8.963418	16.24415	0.568607	0.014398	30.30303	6.25%
13.87091	16.41331	4.722237	7.388812	8.388159	5.949457	0.665376	0.003303	10.03494	6.25%
20.60378	21.43642	8.580898	45.34887	14.18456	13.02344	0.665911	0.019368	26.34253	6.25%
2.29671	4.328418	2.385706	2.612131	3.454982	7.987651	0.639351	0.030729	5	6.25%
7.741654	4.409633	3.199062	6.662468	3.305618	2.810902	0.512687	0.001492	7.308795	6.25%
3.822484	3.04199	1.483537	3.818251	1.908441	1.373026	0.555624	0.007592	5.646612	0.00%
41.46435	31.20589	9.32858	48.14999	21.12095	19.71352	0.656718	0.003303	24.63809	6.25%
5.616242	20.12277	5.184713	1.653637	7.798501	4.276689	0.549238	0.004232	6.38975	6.25%
7.334815	3.992227	6.705386	5.670576	10.0421	6.440793	0.619796	0.006045	8.478009	0.00%
55.8742	58.32636	14.61139	17.6581	10.63126	5.387836	0.544105	0.039784	19.92696	6.25%
5.917563	10.70939	3.428641	10.6686	5.422736	6.154865	0.50257	0.02131	5.970084	6.25%
3.43763	7.780697	2.576382	4.205982	2.645895	2.427805	0.471647	0.025671	8.027474	6.25%
16.88516	17.4707	56.10221	4.287858	12.98986	3.388964	0.504163	0.030729	14.00475	0.00%
18.21179	23.35783	4.826988	14.59002	8.027366	4.162892	0.521581	0.017575	15.65827	6.25%
24.7795	25.87903	4.703609	13.26762	7.755983	4.274422	0.525001	0.036558	21.00423	0.00%
13.84439	13.76503	2.067137	8.119476	13.62369	8.770934	0.459096	0.019368	12.11307	0.00%
42.5473	25.0644	7.283448	10.16288	17.49305	26.34536	0.514866	0.001492	17.18206	6.25%
8.255495	6.186945	2.674596	2.369294	3.999597	2.6613	0.571418	0.04323	8.202518	6.25%
6.143554	3.24282	1.588354	2.313599	3.433788	1.667315	0.533399	0.039784	5	6.25%
11.64368	39.60479	22.98974	9.884935	28.18116	20.48164	0.455141	0.000327	17.99478	0.00%
5.917322	13.13125	18.67557	7.269908	10.23274	6.04296	0.497851	0.017575	11.56443	0.00%
11.28458	3.225321	31.1755	32.82202	12.03103	23.45781	0.290327	0.000233	19.18399	0.00%
31.06295	37.8115	16.57788	26.16051	24.13139	12.88307	0.643874	0.003303	28.09489	6.25%
2.024734	5.471957	2.107895	6.476658	7.098817	11.63005	0.556194	0.008485	5.388401	6.25%
9.040492	13.71891	12.90938	4.484058	7.801736	3.156931	0.645185	0.025671	9.112764	6.25%
14.23218	13.54541	7.396783	11.3158	7.070609	5.41301	0.564607	0.011716	18.19182	6.25%
7.88194	37.54432	7.951591	4.493383	5.591001	4.554186	0.525794	0.002242	6.229966	6.25%
13.16916	44.41999	11.20089	7.817762	19.67925	9.247397	0.558823	0.000972	18.45804	6.25%
6.65315	12.29982	4.717094	22.27529	10.43393	8.072976	0.604002	0.023407	23.36317	6.25%
47.79576	6.991476	4.961455	37.41662	29.09117	13.49589	0.460252	0.019368	26.51683	0.00%
20.75425	11.75945	3.1451	8.951166	31.60078	11.04626	0.54933	0.025671	30.60462	6.25%
14.62021	8.730859	1.729856	2.521224	20.1032	2.555095	0.457827	0.04323	14.43195	6.25%
8.236207	11.20501	4.145282	5.165773	6.336831	23.68698	0.535029	0.005378	11.5199	0.00%
5.984869	2.693739	27.37485	21.57558	7.318564	11.12539	0.283669	0.000277	8.854905	0.00%
3.798335	7.975194	2.686398	3.652887	3.874143	2.992567	0.487959	0.011716	6.444148	0.00%
32.21855	5.659346	3.924615	8.481453	12.26006	27.54885	0.609621	0.036558	12.5277	6.25%
32.64651	46.22147	12.71861	25.56785	12.07846	12.75675	0.639845	0.012999	20.62773	6.25%
6.924764	19.36823	3.968484	4.616529	6.072784	6.706833	0.556838	0.001962	6.18736	6.25%
7.367138	13.69898	4.322396	30.51099	8.859037	10.21094	0.635509	0.011716	12.21877	0.00%

4.409018	15.56978	5.640193	3.722522	5.079679	4.649499	0.599534	0.001124	5	6.25%
17.87838	20.36735	4.316939	19.31684	19.65451	11.17875	0.526837	0.006781	17.29918	0.00%
10.8754	30.6347	9.114461	7.102853	12.27592	9.776435	0.448467	0.000385	10.76211	6.25%
7.395336	10.8347	2.866767	6.983435	2.57541	3.587945	0.572934	0.000385	5.965046	0.00%
7.564732	68.65355	3.996581	9.691139	20.0404	12.18704	0.535041	0.001713	15.60917	6.25%
8.808896	25.19823	3.267693	23.38581	4.602577	4.831409	0.398629	0.019368	11.81947	0.00%
13.87853	8.164516	7.781173	7.884423	6.546473	4.704092	0.600973	0.028108	11.84635	0.00%
18.94268	9.213188	5.438749	12.34313	15.63969	20.18876	0.583971	0.004232	11.81888	0.00%
10.6885	32.82256	9.103203	24.11961	11.15828	50.21494	0.644015	0.036558	27.70834	0.00%
17.4657	9.769056	4.420383	13.6627	8.942448	9.482875	0.585523	0.033542	12.84078	0.00%
32.56509	39.65983	12.05267	15.69231	24.85076	71.64539	0.551417	0.006781	37.41179	0.00%
6.60806	4.692538	17.05755	3.577091	12.24166	2.424066	0.548656	0.019368	6.664024	0.00%
5.936546	27.35189	16.0143	3.677005	2.893523	2.533081	0.197866	0.04323	5.603177	0.00%
15.83632	7.869823	2.805926	2.988646	6.055377	5.236827	0.370981	0.000452	8.270215	0.00%
25.88326	25.58812	9.447211	26.60137	25.89447	38.82689	0.603589	0.000972	25.92519	0.00%
17.71453	31.71566	12.33692	39.8733	17.07667	22.09565	0.633202	0.015921	19.72742	6.25%
31.48842	75.71901	5.125067	51.73354	10.77212	8.465483	0.341191	0.004232	23.88909	0.00%
13.98314	15.27251	9.489258	11.68184	9.006106	6.821895	0.644564	1.48E-05	8.886251	6.25%
7.098914	4.541629	2.838723	2.346076	7.73252	6.978545	0.556947	0.007592	5	0.00%
6.053499	9.72092	8.039973	4.953515	8.197713	17.26375	0.565685	0.011716	15.63783	0.00%
8.329525	5.099947	3.474885	3.947654	9.059569	8.920644	0.575429	0.006045	6.913209	0.00%
2.924646	12.13625	1.203583	5.236692	4.877089	2.430305	0.569985	0.028108	9.011148	6.25%
8.173645	8.451619	3.797538	20.88115	7.55635	6.419042	0.663986	0.025671	7.712346	0.00%
20.33041	3.677544	2.596506	8.907266	35.29679	13.62723	0.355515	0.01054	8.211845	0.00%
4.702796	16.58968	19.67022	7.411607	7.7073	6.027507	0.476797	0.036558	11.92857	0.00%
10.91125	11.16192	2.832741	7.738022	22.06525	9.95223	0.342322	0.001124	12.50296	0.00%
2.824188	30.74497	2.271551	14.0116	9.336176	7.793076	0.57508	0.046906	12.11435	6.25%
4.609739	10.60829	2.23309	0.882603	10.02422	3.16969	0.539197	0.005378	7.566897	6.25%
6.546983	4.502587	4.496349	5.296949	2.905566	4.363398	0.65503	0.025671	6.841245	6.25%
4.982168	4.032065	3.08826	4.130501	3.671379	6.196353	0.540332	0.001492	5	6.25%
19.04556	18.95157	13.76904	10.08768	7.818534	12.79716	0.620305	0.019368	19.75463	6.25%
14.26562	15.48338	10.75205	23.803	12.26811	6.057796	0.401365	7.86E-05	9.881048	0.00%
21.80452	34.55313	11.32913	3.384195	24.8829	10.76758	0.448893	0.001713	17.25214	6.25%
15.38123	53.03536	16.74804	35.27909	32.86044	44.47865	0.492961	0.003743	33.98078	0.00%
34.06082	13.32171	7.192236	7.015101	5.744644	3.425016	0.513721	0.006045	6.079159	6.25%
38.74314	19.02386	10.98857	9.125375	7.919584	4.562518	0.530758	0.008485	6.975978	6.25%
7.3503	14.26328	8.031973	2.986588	22.32511	6.407054	0.552184	0.030729	13.26238	0.00%
34.82781	32.89542	10.52624	8.28082	10.47335	12.39751	0.585117	0.039784	20.50627	6.25%
7.204951	16.79633	8.145185	13.4772	11.58212	8.531838	0.604401	0.009466	11.27361	0.00%
18.12807	49.51031	14.90603	21.94944	31.67352	21.16225	0.599658	0.003303	24.91896	6.25%
39.30947	26.35327	16.6795	1.927615	6.050554	10.04912	0.574469	0.04323	15.36668	6.25%
46.00989	23.5317	6.618924	14.11352	21.43133	24.17739	0.573286	0.001962	23.02531	0.00%
31.08884	18.1259	6.016285	13.24884	16.42629	19.68128	0.605738	0.003303	17.88827	6.25%
7.221981	17.37418	2.482585	2.2189	8.173033	4.347423	0.645722	0.028108	7.95965	6.25%
10.78817	5.884359	4.992443	1.84744	2.227751	1.24997	0.584928	0.046906	5.126573	6.25%
26.4664	7.705627	3.910565	7.204303	10.3569	19.09172	0.544475	0.019368	17.95548	0.00%
68.96394	94.40596	24.49075	79.81695	46.11567	25.50216	0.527609	0.000838	51.45557	6.25%
56.62927	46.06389	14.18283	39.26572	41.72056	23.68334	0.648643	0.009466	44.99684	0.00%
5.4034	9.203775	2.979528	3.77615	15.75069	6.318626	0.661275	0.015921	6.289591	6.25%
10.62491	15.33069	3.509468	15.73138	24.12022	11.14935	0.325526	0.006781	12.13978	0.00%
1.457154	1.419777	14.56352	2.54376	2.340893	2.345682	0.264295	0.011716	5	6.25%
19.36541	13.46524	3.017604	3.191603	8.993468	5.943402	0.484815	0.004775	8.194098	0.00%
19.53943	51.08001	17.21509	49.50671	27.48747	36.4996	0.60524	0.014398	35.55161	6.25%

positiveHC	LOG2(fold	-LOG10(p-value)
0.00%	1.133795	3.558278
0.00%	0.819568	3.208254
6.25%	1.61094	3.208254
0.00%	2.092758	2.949296
0.00%	1.235219	2.887261
0.00%	1.233291	2.826282
0.00%	0.903386	2.649347
0.00%	1.552189	2.592325
0.00%	1.528448	2.536234
6.25%	1.834658	2.481081
6.25%	0.630919	2.481081
0.00%	1.380093	2.37348
6.25%	0.66823	2.37348
0.00%	2.10116	2.321002
0.00%	1.93872	2.321002
6.25%	1.170271	2.269398
0.00%	2.040455	2.218637
0.00%	2.369082	2.218637
0.00%	0.883324	2.218637
6.25%	0.855127	2.218637
6.25%	1.111469	2.168721
0.00%	1.060849	2.119622
6.25%	1.130516	2.119622
0.00%	1.363974	2.071342
0.00%	0.990147	2.071342
6.25%	0.938085	2.071342
0.00%	1.434582	2.023855
0.00%	0.863562	2.023855
6.25%	1.937174	2.023855
6.25%	0.680029	2.023855
0.00%	1.904116	1.977161
6.25%	1.039969	1.977161
6.25%	1.682396	1.931238
0.00%	1.324893	1.886086
6.25%	1.497354	1.886086
0.00%	1.230835	1.886086
6.25%	1.495349	1.886086
6.25%	0.612554	1.886086
6.25%	1.527568	1.841683
0.00%	1.510803	1.798029
0.00%	1.104514	1.798029
0.00%	1.301843	1.798029
0.00%	2.049378	1.798029
0.00%	1.165782	1.755104
0.00%	0.909972	1.755104
0.00%	0.602298	1.755104
6.25%	0.879685	1.755104

6.25%	0.853234	1.755104
0.00%	0.743318	1.712908
0.00%	1.007823	1.712908
6.25%	1.008613	1.712908
6.25%	1.478782	1.712908
6.25%	0.625043	1.712908
0.00%	1.764862	1.671422
0.00%	0.745334	1.671422
0.00%	0.750367	1.671422
6.25%	0.921652	1.671422
6.25%	1.205657	1.671422
6.25%	0.930848	1.671422
6.25%	0.606892	1.671422
0.00%	1.26779	1.630645
0.00%	1.441081	1.630645
0.00%	1.806243	1.630645
0.00%	1.546794	1.630645
6.25%	0.632229	1.630645
6.25%	1.405161	1.630645
0.00%	2.046741	1.590561
0.00%	1.261352	1.590561
0.00%	1.242043	1.590561
6.25%	1.516709	1.590561
6.25%	0.715574	1.590561
6.25%	0.711825	1.590561
0.00%	0.799049	1.551168
0.00%	0.900718	1.551168
6.25%	1.372895	1.551168
6.25%	0.692312	1.551168
0.00%	0.661445	1.51245
6.25%	1.486906	1.51245
6.25%	0.695316	1.51245
0.00%	1.578616	1.474406
0.00%	1.92664	1.474406
0.00%	1.021736	1.474406
6.25%	1.155864	1.474406
6.25%	1.286428	1.474406
6.25%	1.216968	1.474406
0.00%	1.185666	1.437022
6.25%	0.938122	1.437022
0.00%	2.664722	1.400294
6.25%	1.437772	1.400294
6.25%	1.554834	1.400294
6.25%	1.063934	1.400294
0.00%	0.972833	1.400294
0.00%	1.844748	1.364211
6.25%	1.518501	1.364211
6.25%	0.875521	1.364211
6.25%	3.420092	1.364211
0.00%	1.287201	1.32877
0.00%	1.251474	1.32877
0.00%	0.596348	1.32877
0.00%	1.113511	1.32877
6.25%	0.626171	1.32877

6.25%	0.76627	2.707344
0.00%	0.792471	4.10477
0.00%	0.976729	4.10477
0.00%	0.646416	2.269398
6.25%	0.654418	2.592325
6.25%	0.585751	1.551168
6.25%	0.73894	2.949296
0.00%	1.043329	2.649347
0.00%	0.81876	1.474406
0.00%	0.629186	2.426828
0.00%	0.587671	2.536234
6.25%	0.949941	1.364211
0.00%	0.884788	2.887261
6.25%	0.994059	2.481081
0.00%	0.680983	2.321002
6.25%	1.501476	1.364211
0.00%	0.598818	1.931238
0.00%	0.708612	1.551168
6.25%	0.753745	2.766305
0.00%	0.627703	1.841683
0.00%	0.684504	1.931238
6.25%	0.602376	1.841683
6.25%	0.874334	1.51245
6.25%	0.636392	1.630645
6.25%	0.681034	1.630645
31.25%	-2.17786	2.37348
43.75%	-1.58489	1.364211
50.00%	-1.02597	2.321002
31.25%	-0.96205	1.630645
37.50%	-1.27696	2.321002
31.25%	-0.78192	1.437022
37.50%	-1.74574	1.977161
43.75%	-2.24524	1.671422
31.25%	-1.03537	1.51245
37.50%	-1.05666	1.886086
43.75%	-1.04199	2.887261
31.25%	-0.8147	2.649347
31.25%	-1.26796	3.414552
31.25%	-0.77899	1.437022
31.25%	-1.04807	1.630645
37.50%	-2.20357	1.32877
31.25%	-0.72265	1.755104
31.25%	-1.81085	1.755104
31.25%	-0.77893	2.481081
31.25%	-1.20668	1.798029
43.75%	-1.06069	2.071342
31.25%	-0.84537	1.32877
31.25%	-0.7641	2.119622
43.75%	-1.84406	2.949296
31.25%	-1.10862	2.649347
31.25%	-0.6119	2.168721
31.25%	-0.74848	2.218637
37.50%	-0.58506	1.931238
43.75%	-0.77826	2.119622

43.75%	-0.73731	1.755104
31.25%	-0.75062	2.321002
37.50%	-1.16888	2.649347
56.25%	-0.86892	2.766305
37.50%	-0.95202	2.766305
56.25%	-0.66825	3.141826
43.75%	-0.73654	2.536234
50.00%	-0.60408	2.071342
50.00%	-0.93566	3.141826
31.25%	-0.95794	2.119622
37.50%	-0.73297	1.551168
31.25%	-0.61462	1.931238
43.75%	-1.3494	2.536234
31.25%	-1.00049	2.949296
31.25%	-0.69564	2.218637
37.50%	-0.63276	2.536234
37.50%	-0.68689	2.168721
31.25%	-0.66917	1.671422
37.50%	-0.86494	3.208254
37.50%	-0.86557	2.218637
37.50%	-0.68308	1.474406
31.25%	-0.67845	1.474406
43.75%	-1.46284	4.451676
37.50%	-0.97673	1.977161
31.25%	-0.62545	1.798029
31.25%	-0.72494	1.931238
37.50%	-0.96091	1.590561
31.25%	-0.75235	1.437022
50.00%	-0.61322	2.592325
37.50%	-0.88088	1.841683
31.25%	-1.5732	1.630645
43.75%	-1.72436	3.275815
31.25%	-0.69025	2.426828
37.50%	-0.90203	2.321002
37.50%	-1.49035	1.551168
37.50%	-0.74323	1.437022
31.25%	-0.66906	1.32877
31.25%	-1.67933	1.32877
37.50%	-0.81469	1.400294
37.50%	-0.86775	1.437022
31.25%	-1.72234	1.590561
43.75%	-0.63312	1.886086
50.00%	-0.98515	2.949296
37.50%	-0.71595	3.012374
62.50%	-0.73297	2.426828
31.25%	-0.81364	2.649347
50.00%	-1.98003	1.474406
31.25%	-1.21807	2.119622
37.50%	-0.93574	3.141826
37.50%	-0.68902	1.630645
37.50%	-1.2679	2.592325
56.25%	-0.73577	1.841683
43.75%	-0.74373	1.437022
50.00%	-0.90734	2.766305

43.75%	-0.64989	2.269398
31.25%	-0.66964	1.671422
31.25%	-1.26043	3.485798
31.25%	-0.58881	1.590561
43.75%	-0.78825	1.671422
31.25%	-0.79931	2.321002
31.25%	-0.81973	1.51245
31.25%	-0.97233	2.826282
31.25%	-0.82968	2.023855
31.25%	-0.71189	2.37348
31.25%	-0.67228	1.630645
31.25%	-1.44494	2.218637
31.25%	-0.67272	2.071342
31.25%	-0.82706	1.437022
37.50%	-0.71318	3.414552
31.25%	-0.8145	1.841683
43.75%	-0.58776	2.481081
31.25%	-0.5866	1.712908
31.25%	-0.64532	1.51245
37.50%	-0.96385	2.826282
31.25%	-0.84782	2.119622
43.75%	-0.60665	2.481081
37.50%	-0.8645	2.37348
31.25%	-0.69014	2.218637
31.25%	-0.87804	1.400294
31.25%	-0.9926	1.671422
31.25%	-1.08422	1.590561
31.25%	-0.98804	1.51245
50.00%	-0.93904	1.755104
37.50%	-0.92961	1.437022
37.50%	-1.12313	1.712908
50.00%	-0.95773	2.826282
31.25%	-0.80738	1.364211
37.50%	-0.90671	1.400294
56.25%	-1.13561	3.485798
37.50%	-1.00621	1.755104
56.25%	-1.78425	3.632119
50.00%	-0.63515	2.481081
37.50%	-0.84634	2.071342
37.50%	-0.63222	1.590561
31.25%	-0.82468	1.931238
31.25%	-0.92743	2.649347
37.50%	-0.83954	3.012374
31.25%	-0.72737	1.630645
31.25%	-1.11951	1.712908
31.25%	-0.86426	1.590561
31.25%	-1.12713	1.364211
37.50%	-0.90231	2.269398
62.50%	-1.81772	3.558278
37.50%	-1.03517	1.931238
43.75%	-0.71402	1.437022
50.00%	-0.6442	1.886086
50.00%	-0.84467	2.707344
43.75%	-0.65402	1.931238

50.00%	-0.73809	2.949296
50.00%	-0.92457	2.168721
50.00%	-1.15693	3.414552
31.25%	-0.80356	3.414552
43.75%	-0.90228	2.766305
43.75%	-1.32688	1.712908
37.50%	-0.73463	1.551168
37.50%	-0.77603	2.37348
31.25%	-0.63483	1.437022
43.75%	-0.7722	1.474406
37.50%	-0.85878	2.168721
31.25%	-0.86603	1.712908
37.50%	-2.3374	1.364211
43.75%	-1.43058	3.344592
31.25%	-0.72836	3.012374
31.25%	-0.65926	1.798029
43.75%	-1.55135	2.37348
87.50%	-0.63361	4.82847
37.50%	-0.84439	2.119622
37.50%	-0.82193	1.931238
43.75%	-0.79729	2.218637
31.25%	-0.811	1.551168
31.25%	-0.59077	1.590561
56.25%	-1.49202	1.977161
37.50%	-1.06855	1.437022
43.75%	-1.54658	2.949296
37.50%	-0.79817	1.32877
31.25%	-0.89112	2.269398
31.25%	-0.61037	1.590561
31.25%	-0.88808	2.826282
31.25%	-0.68895	1.712908
56.25%	-1.31701	4.10477
43.75%	-1.15556	2.766305
37.50%	-1.02046	2.426828
37.50%	-0.96094	2.218637
56.25%	-0.91387	2.071342
31.25%	-0.85678	1.51245
43.75%	-0.7732	1.400294
31.25%	-0.72642	2.023855
37.50%	-0.73779	2.481081
31.25%	-0.7997	1.364211
50.00%	-0.80267	2.707344
56.25%	-0.72323	2.481081
37.50%	-0.63102	1.551168
31.25%	-0.77367	1.32877
31.25%	-0.87706	1.712908
37.50%	-0.92246	3.076559
31.25%	-0.6245	2.023855
37.50%	-0.59668	1.798029
31.25%	-1.61916	2.168721
43.75%	-1.91978	1.931238
43.75%	-1.04449	2.321002
31.25%	-0.72442	1.841683
